# Regulation and physiological function of proteins for heat tolerance in cowpea (*Vigna unguiculata*) genotypes under controlled and field conditions

**DOI:** 10.3389/fpls.2022.954527

**Published:** 2022-08-22

**Authors:** Tonny I. Selinga, Sipho T. Maseko, Hawwa Gabier, Mohammed S. Rafudeen, A. Muthama Muasya, Olivier Crespo, John B. O. Ogola, Alex J. Valentine, Carl-Otto Ottosen, Eva Rosenqvist, Samson B. M. Chimphango

**Affiliations:** ^1^Department of Biological Sciences, University of Cape Town, Rondebosch, South Africa; ^2^Department of Crop Sciences, Tshwane University of Technology, Pretoria, South Africa; ^3^Department of Molecular and Cell Biology, University of Cape Town, Rondebosch, South Africa; ^4^Climate System Analysis Group, Department of Environmental and Geographical Science, University of Cape Town, Rondebosch, South Africa; ^5^Department of Plant and Soil Sciences, University of Venda, Thohoyandou, South Africa; ^6^Department of Botany and Zoology, University of Stellenbosch, Stellenbosch, South Africa; ^7^Department of Food Science, Aarhus University, Aarslev, Denmark; ^8^Section for Crop Science, Department of Plant and Environmental Sciences, University of Copenhagen, Taastrup, Denmark

**Keywords:** cowpea, heat shock proteins, label-free protein quantification, plant proteomics, thermotolerance mechanisms

## Abstract

The expression of heat shock proteins is considered a central adaptive mechanism to heat stress. This study investigated the expression of heat shock proteins (HSPs) and other stress-protective proteins against heat stress in cowpea genotypes under field (IT-96D-610 and IT-16) and controlled (IT-96D-610) conditions. Heat stress response analysis of proteins at 72 h in the controlled environment showed 270 differentially regulated proteins identified using label-free quantitative proteomics in IT-96D-610 plants. These plants expressed HSPs and chaperones [BAG family molecular chaperone 6 (BAG6), Multiprotein bridging factor1c (MBF1C) and cold shock domain protein 1 (CSDP1) in the controlled environment]. However, IT-96D-610 plants expressed a wider variety of small HSPs and more HSPs in the field. IT-96D-610 plants also responded to heat stress by exclusively expressing chaperones [DnaJ chaperones, universal stress protein and heat shock binding protein (HSBP)] and non-HSP proteins (Deg1, EGY3, ROS protective proteins, temperature-induced lipocalin and succinic dehydrogenase). Photosynthesis recovery and induction of proteins related to photosynthesis were better in IT-96D-610 because of the concurrent induction of heat stress response proteins for chaperone functions, protein degradation for repair and ROS scavenging proteins and PSII operating efficiency (Fq’/Fm′) than IT-16. This study contributes to identification of thermotolerance mechanisms in cowpea that can be useful in knowledge-based crop improvement.

## Introduction

Rising food insecurity and malnutrition of millions of Africans have been driven by extreme climate events between 2015 and 2019 (Trisos et al., 2022). This is mainly because the global average temperatures are increasing due to climate change, causing increased heat stress, and negatively affecting crop production (Bokszczanin et al., 2013). Seasonal temperature increases of 1°C are predicted to reduce food crop production by 2.5–16% in the tropics and subtropics where cowpea is cultivated (Lobell et al., 2008; Gonçalves et al., 2016). Cowpea is a source of low-cost protein in the human diet (Gonçalves et al., 2016) and it is a relatively heat- and drought-tolerant crop, but the yields of the sensitive varieties can be reduced by heat stress (Hall, 2012). Comparison of genotypes to identify natural variation in heat tolerance has been suggested as potentially effective way to breed heat-tolerant crops (Ding et al., 2020). Major progress has been made in understanding the molecular mechanisms of heat tolerance in some species (Ding et al., 2020; Janni et al., 2020). However, molecular mechanisms of heat tolerance in Arabidopsis have comprised most of the molecular heat stress response studies and (Ding et al., 2020; Janni et al., 2020) while mechanisms of cowpea response to heat stress are still unclear.

Heat stress causes direct disturbances in protein homeostasis through protein denaturation and aggregation, leading to the inactivation of proteins in chloroplast and mitochondria (Zhao et al., 2021). Photosynthetic enzymes and proteins such as Rubisco and photosystem II (PSII) and jasmonic acid responses such as allene oxide synthase expression are inhibited by heat stress in leaves (Acosta et al., 2013; Du et al., 2013; Liu et al., 2014; Li et al., 2019). Plants respond to abiotic stress mainly through a Ca^2+^ signalling cascade that sends the signal *via* calcium calmodulin 3 (CAM3) to transcription regulators {i.e., Dehydration-Responsive Element Binding protein 2A (DREB2A), heat shock factors (HSFA1s and HSFA2s), Multiprotein Bridging Factor 1C (MBF1c); and NAC [for NAM (no apical meristem), ATAF, CUC (cup-shaped cotyledon)], WRKY (with a WRKY amino acid sequence DNA-binding domain), bZIP (basic leucine zipper), MYB (myeloblastosis) transcription factors; Ohama et al., 2017; Zhao et al., 2021}. Transcription regulators convert the signal to induce heat shock genes to be translated to heat shock proteins (HSPs) as a part of the thermotolerance response (Devireddy et al., 2021; Zhao et al., 2021). Another major signalling pathway counteracting heat stress involves reactive oxygen species (ROS) being converted to hydrogen peroxide (H_2_O_2_) signal and interacting with proteins in the Ca^2+^ signalling pathway (Devireddy et al., 2021). However, when ROS accumulate to toxic levels, the plant uses energy to induce ROS scavenging enzymes and detoxification proteins (Choudhury et al., 2017; Devireddy et al., 2021). WRKY transcription regulators play a key role in stress tolerance and are induced by abscisic acid (ABA) that is synthesized as a part of the heat stress response (Rushton et al., 2012; Devireddy et al., 2021). It is still unclear what other factors are involved in heat stress response signalling cascades (Ohama et al., 2017).

Heat shock proteins, whose main function is to properly fold proteins that are heat-denatured so that the plant can cope with heat stress, are considered the central adaptive response against heat stress in plants (Ohama et al., 2017). The HSPs are induced in both animals and plants in different tissues, and their expression is fast and universal under heat stress (Wahid et al., 2007), a trait essential for heat tolerance (Vierling, 1991). Among the HSPs, small heat shock proteins (sHSPs) are the most induced proteins with up to 200-fold changes during heat stress (Wang et al., 2004). Specific HSPs are induced under certain conditions because of plant specialized programs (Echevarría-Zomeño et al., 2016) and most HSPs are induced in plants at temperatures above 35°C with a few exceptions, that are induced at lower temperatures of 27°C (Waters et al., 1996; Tanaka et al., 2000; Jiang et al., 2017). Few studies have focused on the cowpea gene and protein responses of HSPs to heat stress under controlled conditions (Janni et al., 2020). The heat shock protein 17.7 (VuHSP17.7) gene was expressed in cowpea leaves after exposure to heat stress of 40°C for 2 h (Simoes-Araujo et al., 2008). Cowpea, being a known heat-tolerant crop is expected to fully express HSPs and other heat-responsive proteins. A previous study of proteins from suspended cowpea cells revealed that heat shock proteins 70 and 90 expressed at 38°C were a part of the thermotolerance response (Heuss-Larosa et al., 1987). Recently, proteomic analysis of imbibed cowpea seeds led to the identification of 20 unique proteins which were heat stable at 100°C (Zhang and Li, 2015). Those which were involved in the protection of seeds from abiotic stress included late-embryogenesis abundant proteins, Cu/Zn superoxide dismutase and 17.4 kDa Class I heat shock protein. Studies on HSP and other stress related proteins can unravel tolerance mechanisms and identify tolerant plants (Kosová et al., 2018).

Under controlled conditions, most proteomics studies on heat stress expose plants to heat stress from 1 h to 3 days but rarely longer than a week (Janni et al., 2020) thus not revealing the ability to recover short term under fluctuating conditions. As a result, protein expression in chickpea (*Cicer arietinum*) genotypes exposed to heat stress under controlled climate chamber conditions (Parankusam et al., 2017) differed from that of chickpeas probably due to differences in genotypes, growth conditions and levels of heat stress (Makonya et al., 2021). It is known that heat stress in controlled environments can induce proteomic responses that are different from those induced by combined stress which often occurs in the field (Mittler, 2006; Mittler and Blumwald, 2010; Kosová et al., 2018). Furthermore, the results of controlled environment studies may be difficult to translate to improved heat-tolerant genotypes capable of surviving in the field, often with multiple stresses (Passioura, 2020) and with a long period of constant heat stress. Therefore, controlled experiments can provide the context where heat stress responses are viable as not all heat stress responses found in controlled stable environments are relevant to deciphering heat tolerance mechanisms under field conditions (Feder and Hofmann, 1999). Elucidating the molecular basis for the heat stress response in cowpea grown under field conditions by identifying key genes and proteins directly involved in heat tolerance will contribute to information that can be used in molecular breeding thermotolerant cowpea cultivars.

Therefore, this study aimed to investigate heat shock and other stress-protective proteins against heat stress in cowpea genotypes IT-16 (determinate growth) and IT-96D-610 (indeterminate growth type) under field conditions using label-free quantitative proteomics. The specific objectives were two-fold, first, to determine the identity and level of expression of heat shock and other heat stress tolerance proteins in cowpea under controlled and field conditions. Second, to determine whether a drought-tolerant IT-96D-610 genotype (Belko et al., 2014) has a greater expression of heat shock and other heat stress tolerance proteins than IT-16. Indeterminate genotypes have been shown to have better tolerance to dry environments than determinate genotypes in dry bean (*Phaseolus vulgaris*) and soybean (*Glycine max* L. Merrill) because of the ability to regrow leaves and flowers after drought stress (Cuellar-Ortiz et al., 2008; da Silva et al., 2018). This is usually because indeterminate genotypes are more efficient in carbon translocation indicated by better seed yield under drought stress than determinate genotypes (Rosales-Serna et al., 2004; Cuellar-Ortiz et al., 2008). With limited plant exposure time to heat stress under controlled environment, we hypothesized that cowpea will have a lower expression of heat shock and other heat stress tolerance proteins than those in the field and that IT-96D-610 has a greater expression of heat shock and other heat stress tolerance proteins than the IT-16 genotype.

## Materials and methods

### Description of field sites

**Figure 1:**
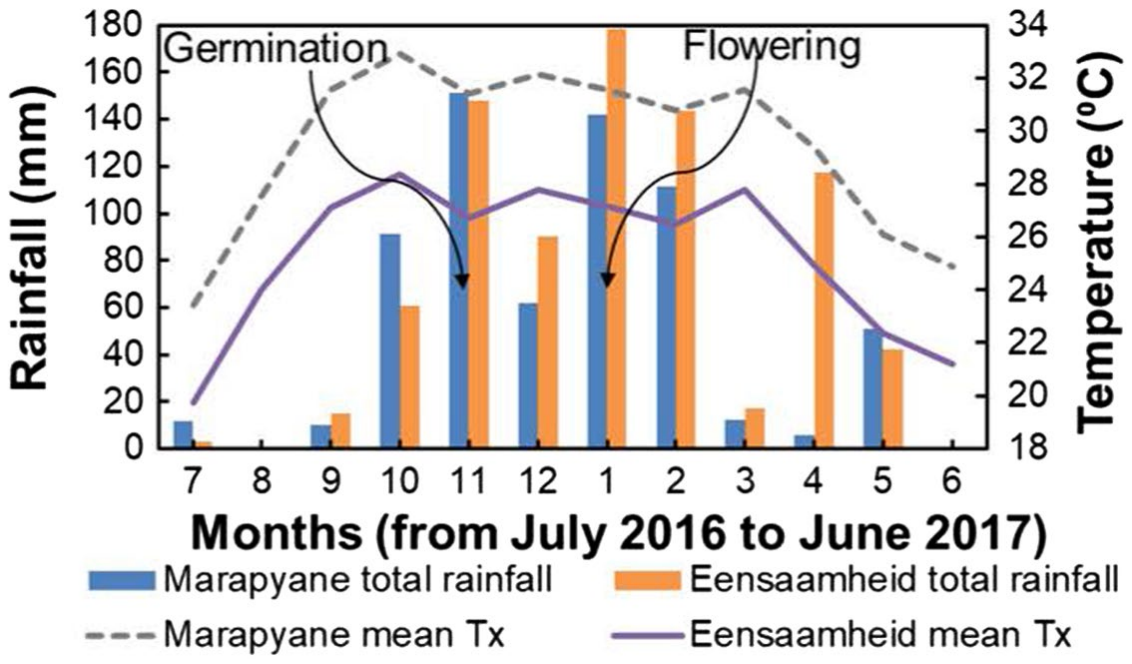
Total rainfall and maximum temperature at Marapyane and Eensaamheid during 2016 to 2017 growing season. Lines with arrows indicate the time of germination and flowering in both sites.

The field experiment was conducted in the province of Mpumalanga in north-eastern South Africa at the Marapyane Agricultural College farm (24°57′60″S; 28°45′76″E and 1,022 m a.s.l.) and Eensaamheid farm (25°53′167″S; 28°57′111″E and 1,511 m a.s.l.) during the 2016–2017 summer growing seasons from November to January. The two sites are 104 km apart (straight line distance). The sites were chosen because they are cowpea growing areas (Agricultural Research Council, 2011) and differed in temperature with mean maximum temperatures in the range of 26–28°C (warm temperatures) at Eensaamheid and 30–32°C (hot temperatures) at Marapyane from germination till flowering and (Figure 1). The total monthly rainfall at Eensaamheid ranged from 92 to 176 mm while that of Marapyane was 50–150 mm from germination to flowering (Figure 1). Generally, regions that are in the northwest of Mpumalanga, such as Marapyane have a lower annual rainfall range (400–600 mm) compared to those in the southwest regions of Mpumalanga (600–800 mm) where Eensaamheid is situated (Lynch, 2004). The rainfall predominantly falls in the summer months between October and April and rarely occurs in the winter months.

### Plant material, experimental design, and management

The source of seeds for the two cowpea genotypes (IT-16 and IT-96D-610) used in this study was the International Institute of Tropical Agriculture (IITA) in Nampula, Mozambique. Genotypes IT-16 was determinate, while IT-96D-610 was indeterminate.

#### Controlled conditions study

A pot experiment was set up in October 2018 in a glasshouse at the University of Cape Town, South Africa (33°57′20″S; 18°27′43″E and 94 m a.s.l) with an average temperature of 24.6°C [standard deviation (SD): ±5.0°C] during the growth period and mean relative humidity of 52.6% (SD: ±11.3%). Promix and commercial sand (Hortishop and Hydroponics, Cape Town, South Africa) were mixed to make up a total of 1.2 kg pot^−1^ in a 1:1 ratio. A fertilizer mixture of Multicoat (4*) 15–3-12 + Mg + micronutrients (Haifa chemicals, South Africa) at 14.5 g pot^−1^ and gypsum (1 g of CaSO_4_ ·2H_2_O pot^−1^) was also mixed with promix and commercial sand mixture in each of 24 pots for IT-96D-610 genotype. Genotype IT-96D-610 was chosen for the glasshouse experiment because it was shown that it had better growth than some genotypes under heat stress in our preliminary experiments. Four seeds of the genotype were sown into each 1 l plastic pot (11 cm in top diameter, 9 cm in depth and 7.5 cm in base diameter) under glasshouse conditions and later thinned to one plant per pot. Plants were grown for 60 days in the glasshouse and were at 50% flowering before exposing them to the heat treatment in the phytotron. A total of 12 plants were subsequently placed in each of two separate phytotron chambers set to control and elevated temperature regimes. Plants in the control phytotron chamber were exposed to temperatures of 25/20°C (day/night) while heat-stressed plants were exposed to 40/25°C. A 13 h photoperiod with supplemental light was set at 1000 μmol m^−2^ s^−1^ from high pressure sodium NAV-E 400 W Ellipsal, metal halide Osram powerstar HQI-E 400 W/DV and incandescent 150 W, 230 V ES type with screw (Osram, Licht AG, Munich, GER) lamps. The humidity was kept high using water baths (approx. 42 to 60% in the control chamber and 50 to 85% in the hot chamber). Plants in both chambers were uniformly treated and watered 300 ml per pot once a day. There were no symptoms of drought stress observed in the hot chamber during the experiment but there were no physiological measurements to confirm the lack of stress symptoms. Therefore, any drought stress that occurred was regarded as minimal at least to the point of no observable wilting of leaves. Leaf samples were taken after 2, 24 and 72 h of heat stress, with four replicate plants per time point for protein extraction.

#### Field study

For the field experiment, two genotypes (IT-16 and IT-96D-610) with four replicate plots at each of the two sites were arranged in a randomized complete block design. Each plot had four plant rows that were 0.75 m apart and 2.7 m long. Planting was done by hand on 03 November (Eensaamheid) and 05 November (Marapyane) 2016. The seeds were sown at 0.15 m intra row spacing and 0.02 m deep without any application of fertilization or irrigation. Throughout the season, the plots were kept weed-free and guarded against cattle with fencing at both sites.

### Proteomics

#### Protein extraction

The three youngest fully expanded leaf samples per plant were harvested from two adjacent plants in the middle row of each plot and combined in the field and four replicate plants from each chamber per time point in the phytotron. Samples were immediately frozen in liquid nitrogen and transported on ice to the laboratory for analysis. On the same day, a phenol extraction protocol (Isaacson et al., 2006) was used to prepare total protein extracts from four biological replicates. Pellets were recovered and impurities were removed three times in chilled methanol (4°C) by centrifuging at 50000 g for 10 min. The pellets were dried in a lamina flow and stored at −80°C and later transported to the Centre for Proteomic and Genomic Research (Cape Town, WC, South Africa) for analysis.

#### Protein solubilization and quantification

TEAB buffer [1,000 mM triethylammonium bicarbonate (TEAB, Sigma T7408; Sigma-Aldrich, St. Louis, MO, United States), 2% sodium dodecyl sulphate (SDS, Sigma 71,736; Sigma-Aldrich, St. Louis, MO, United States), 5% SDS 50 mM TEAB] was used to suspend protein pellets. After heating for 10 min at 96°C, protein samples were centrifuged at 10000 g for 10 min. Proteins were quantified using the QuantiPro BCA assay Kit (Sigma QPBCA; Sigma-Aldrich, St. Louis, MO, United States) by following the manufacturer’s protocol.

#### On-bead hydrophilic interaction liquid chromatography (HILIC) and trypsin digestion

To reduce the proteins, tris (2-carboxyethyl) phosphine (TCEP; Sigma 646,547; Sigma-Aldrich, St. Louis, MO, United States) was added to 50 μg protein on a protein LoBind plate (Merck, 0030504.100; Merck KGaA, Darmstadt, GER) to a final 10 mM TCEP and incubated for 1 h at 60°C. After cooling, samples were alkylated by incubating for 15 min at room temperature with methylmethanethiosulphonate (MMTS; Sigma 208,795; Sigma-Aldrich, St. Louis, MO, United States) which was added to a final 10 mM MMTS. Aliquots of HILIC magnetic beads (ReSyn Biosciences, HLC010; 2B Scientific, Stonesfield, Oxfordshire., United Kingdom) were prepared followed by disposing of the shipping solution. Then beads were rinsed twice with 250 μl of wash buffer (15% acetonitrile, 100 mM ammonium acetate (Sigma 14,267; Sigma-Aldrich, St. Louis, MO, United States) pH 4.5) each time for 1 min. After which a suspension of beads was prepared to 5 mg/ml with loading buffer (30% acetonitrile, 200 mM ammonium acetate pH 4.5). Equal volumes of HILIC magnetic beads to that of alkylated protein samples were added at a ratio of 5:1 (beads: total protein) and the plate was incubated at 900 revolutions per minute (RPM) on a shaker for 30 min at room temperature to allow the proteins to bind to the beads. The beads were rinsed with 500 μl of 95% acetonitrile for 1 min with four repeats. Trypsin was added to the proteins at a ratio of 1:10 {trypsin solution [Trypsin (Promega PRV5111; Promega, Madison, WI, United States) in 50 mM TEAB]: total protein} and the plates were shaken on a shaker for 4 h at 37°C to allow protein digestion. Then proteins were dried after disposal of the supernatant and suspended with loading buffer (0.1% formic acid, 2.5% acetonitrile) for liquid chromatography.

#### Liquid chromatography mass spectrometry (LC–MS) analysis

Solutions of each peptide sample were made using 0.1% formic acid (Sigma 56,302; Sigma-Aldrich, St. Louis, MO, United States), 2% acetonitrile (Burdick & Jackson BJLC015CS; Honeywell, Charlotte, NC, United States) and analysed through LC–MS using a Q-Exactive quadrupole-Orbitrap mass spectrometer (Thermo Fisher Scientific; Waltham, MA, United States) coupled to a Dionex Ultimate 3,000 nano-UPLC system. Samples were loaded and trapped on a C18 trap column (PepMap100, 9,027,905,000, 300 μm × 5 mm × 5 μm) followed by 3 min of washing. Peptides were eluted on an analytical column after the valve was switched and chromatographically separated using Waters nanoEase (Zenfit) M/Z Peptide CSH C18 column (186,008,810, 75 μm × 25 cm × 1.7 μm). Peptides were eluted with solvent A [liquid chromatography water (Burdick and Jackson BJLC365; Honeywell, Charlotte, NC, United States), 0.1% formic acid] and solvent B (2% acetonitrile, 0.1% formic acid) and separated through a multi-step gradient at 300 nl min^−1^. In the first step, the gradient changed 98–95% in solvent A and 2–5% in solvent B over 5 min and then changed 95–82% (solvent A) and 5–18% (solvent B) over 40 min; 82–70% (solvent A) and 18–30% (solvent B) over 10 min, 70–20% (solvent A) and 30–80% (solvent B) over 2 min. After the gradient was held at 20% (solvent A) and 80% (solvent B) for 10 min, it was finally returned to 98% (solvent A) and 2% (solvent B), then the column was conditioned for 15 min. Data were obtained by operating Proxeon stainless steel emitters (Thermo Fisher TFES523; Waltham, MA, United States). The positive ion mode was used to operate the mass spectrometer at a capillary temperature of 320°C and the electrospray voltage of 1.95 kV was applied. Data from the LC–MS analysis was obtained by operating Xcalibur v4.1.31.9, Chromeleon v6.8 (SR13), Orbitrap MS v2.9 (build 2,926) and Thermo Foundations 3.1 (SP4) software.

#### Protein identification and proteomic analyses

Proteins were quantified label-free using Progenesis QI for Proteomics v2.0 (Nonlinear Dynamics, Newcastle, United Kingdom). The raw data were processed through peak picking, running alignment, and normalization through which singly charged spectra was discarded. Only valid proteins with a minimum of two unique peptides were reported and four biological replicates per condition were quantified using non-conflicting peptides. Regulated proteins were identified as those that have fold change ≥2 and q-value <0.05 where fold change is the number of times by which the protein increased (+ value) or decreased (− value) in stressed plants compared to control (phytotron chamber at day/night temperatures of 25/20°C and Eensaamheid site for the controlled environment and field studies, respectively). Databases were interrogated using Byonic software v2.6.46 (Protein Metrics, Cupertino, CA, United States) and a *V. unguiculata* database with protein accession numbers were sourced from the DOE Joint Genome Institute (Lonardi et al., 2019) and downloaded on 28/09/2018.

Protein annotations were sourced from the Phytozome database using the *V. unguiculata* v1.1 gene set for cowpea genome (Lonardi et al., 2019). The main database, Phytozome 13 (Lonardi et al., 2019) and supporting database, UniProt (The UniProt Consortium, 2019) were used to search for Gene Ontology (GO) terms Biological process, Molecular function and Cellular component of the identified proteins. Functional categories of proteins were classified using Biological processes GO terms and analysed using literature (Zhao et al., 2018; Li et al., 2019; Vessal et al., 2020; Wei et al., 2020) and UniProt (The UniProt Consortium, 2019). For proteins with no information on Biological processes GO terms, orthologues were taken from *Arabidopsis thaliana*, *Medicago truncatula* and *G. max*.

## Results

### Proteomic response of IT-96D-610 plants to heat stress in the controlled environment

There were no differentially regulated proteins from the leaves of IT-96D-610 after 2 and 24 h of heat stress in the controlled environment and, therefore, these were not reported. A total of 1,397 proteins were identified after 72 h of heat stress, and 270 were deferentially regulated, after 72 h. The comparison of heat stress-regulated proteins (HSRPs) in the heat-stressed plants relative to control plants showed that there were 78 heat upregulated proteins whereas 192 proteins were downregulated (Supplementary Table S1). The fold change of heat upregulated proteins ranged from 2.0 to 41.7 and that of downregulated proteins was −2.0 to −152.7.

**Figure 2:**
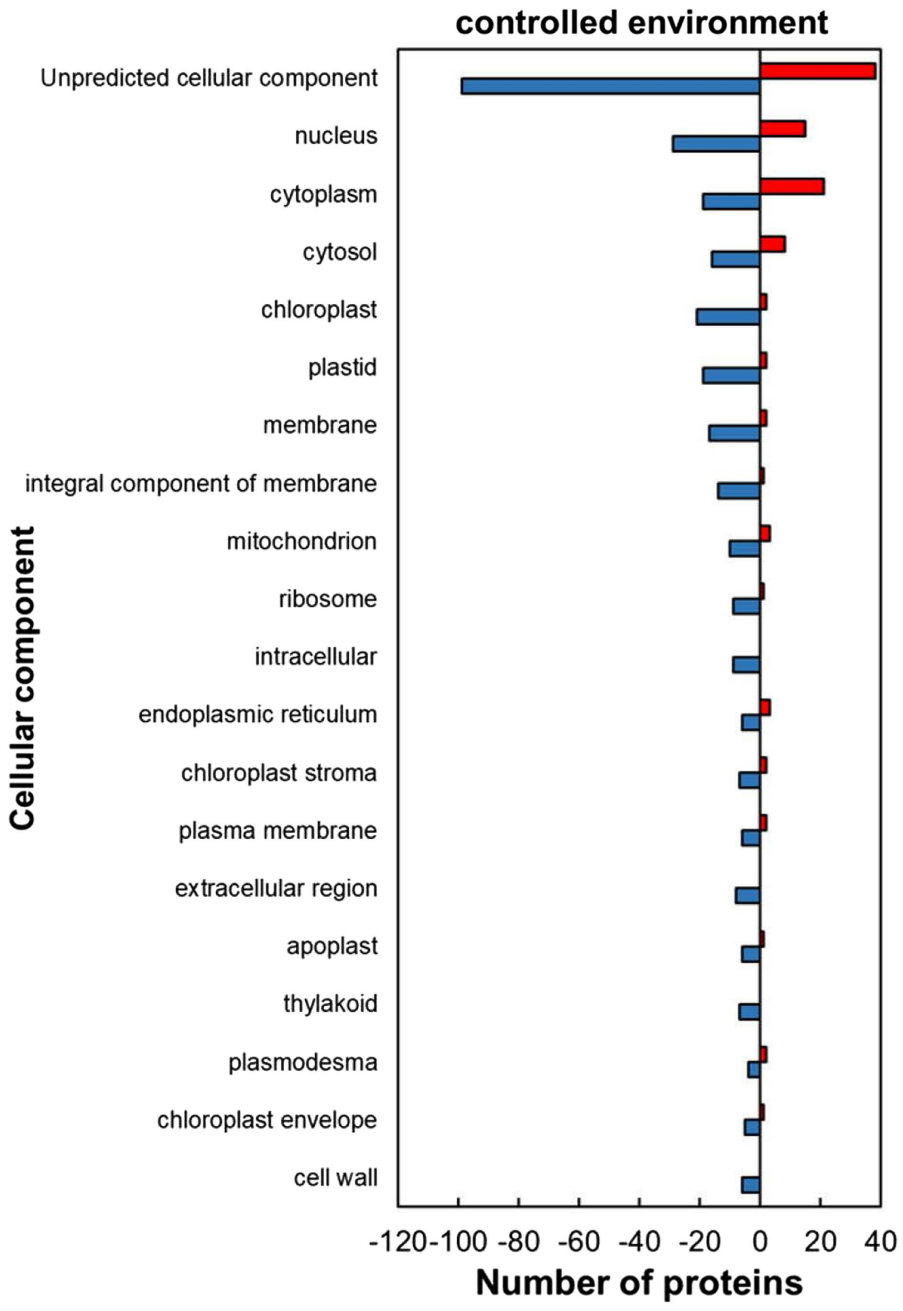
Cellular component of heat upregulated and downregulated proteins from IT-96D-610 from heat-stressed plants relative to the controls in the controlled environment based on GO annotation. Positive numbers (red) represent number of upregulated proteins and negative numbers (blue) the number of downregulated proteins.

The classification of the HSRPs using gene ontology (GO) terms to cellular components showed that almost all the intracellular components and cell boundary structures had many proteins downregulated except for the cytoplasm category which had mostly heat upregulated proteins (21; 52%) in stressed plants compared to the control plants (Figure 2). Examples of the intracellular proteins that were downregulated included those localized in the chloroplast (21; 91%), mitochondrion (10; 77%) and nucleus (29; 66%). Furthermore, HSRPs localized on the cell boundary were mostly downregulated in the integral component of membrane (14; 93%), plasma membrane (6; 75%), extracellular region (3; 100%), apoplast (6; 86%) and cell wall (6; 100%).

**Figure 3:**
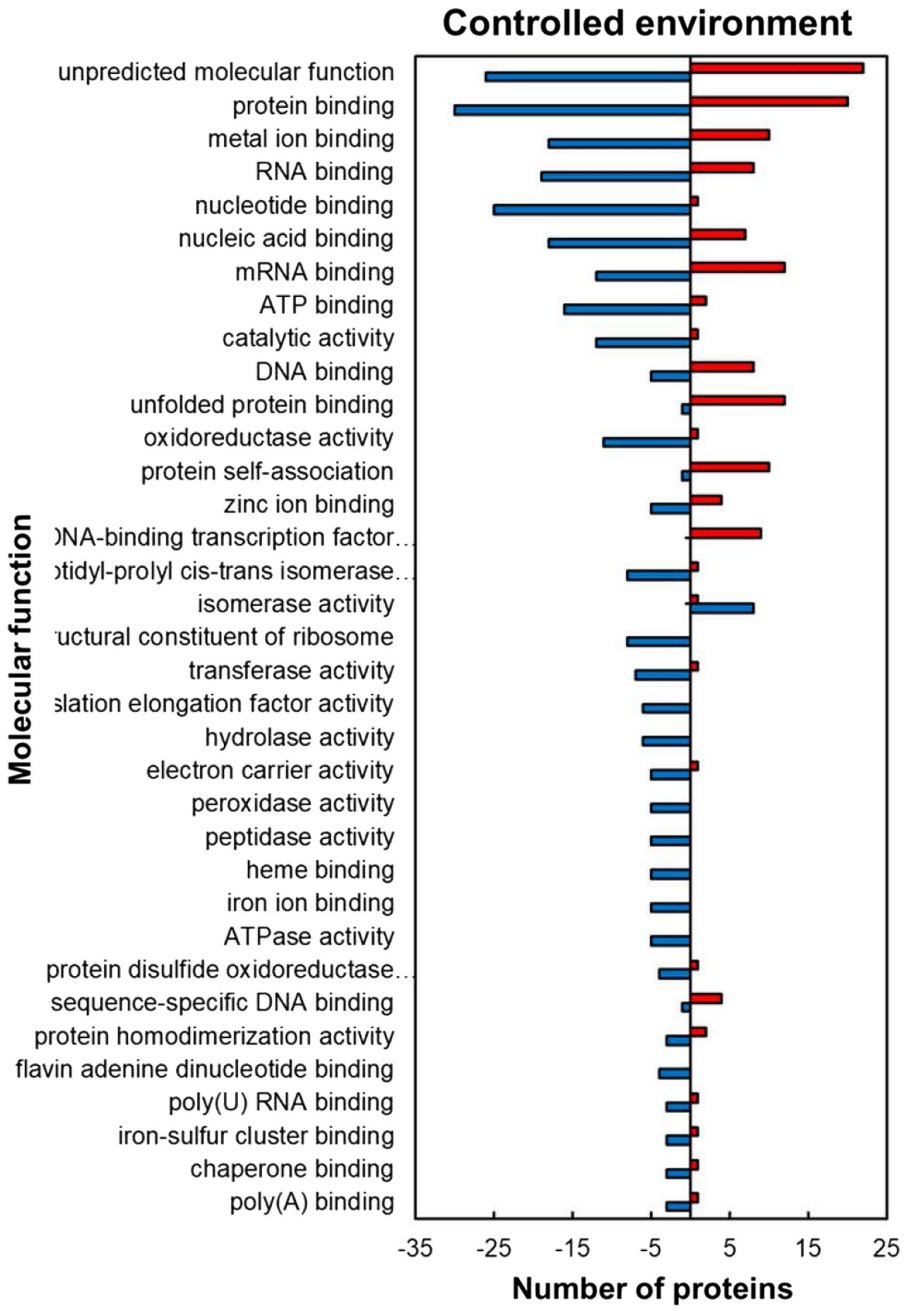
Molecular function prediction of heat upregulated and downregulated proteins from IT-96D-610 from heat-stressed plants relative to the controls in the controlled environment based on GO annotation. Positive numbers (red) represent number of upregulated proteins and negative numbers (blue) the number of downregulated proteins.

For the predicted molecular function GO category, the HSRPs with molecular function of protein and DNA-binding-related activities were heat upregulated while those with RNA and metal ion binding, and antioxidant related activities were heat downregulated in stressed compared to the control plants (Figure 3). Regulated proteins with DNA and protein binding related activities that were mostly upregulated included those with a role in protein binding (30; 60%), unfolded protein binding (12; 92%), protein self-association (10; 91%), DNA-binding transcription factor activity (9; 100%), DNA binding (8; 62%) and sequence-specific DNA binding (4; 80%; Figure 3). In contrast, some of the HSRPs were downregulated and had the function of RNA binding (19; 70%), flavin adenine dinucleotide binding (7; 100%), poly(U) RNA binding (3; 75%) and poly(A) binding (3; 75%), as well as those related to oxidative stress protection function in peroxidase activity (5; 100%). Lastly, HSRPs with metal ion related binding including zinc ion binding (5, 55%), iron ion binding (5; 100%) and iron–sulphur cluster binding (3; 75%) were also downregulated (Figure 3).

**Figure 4:**
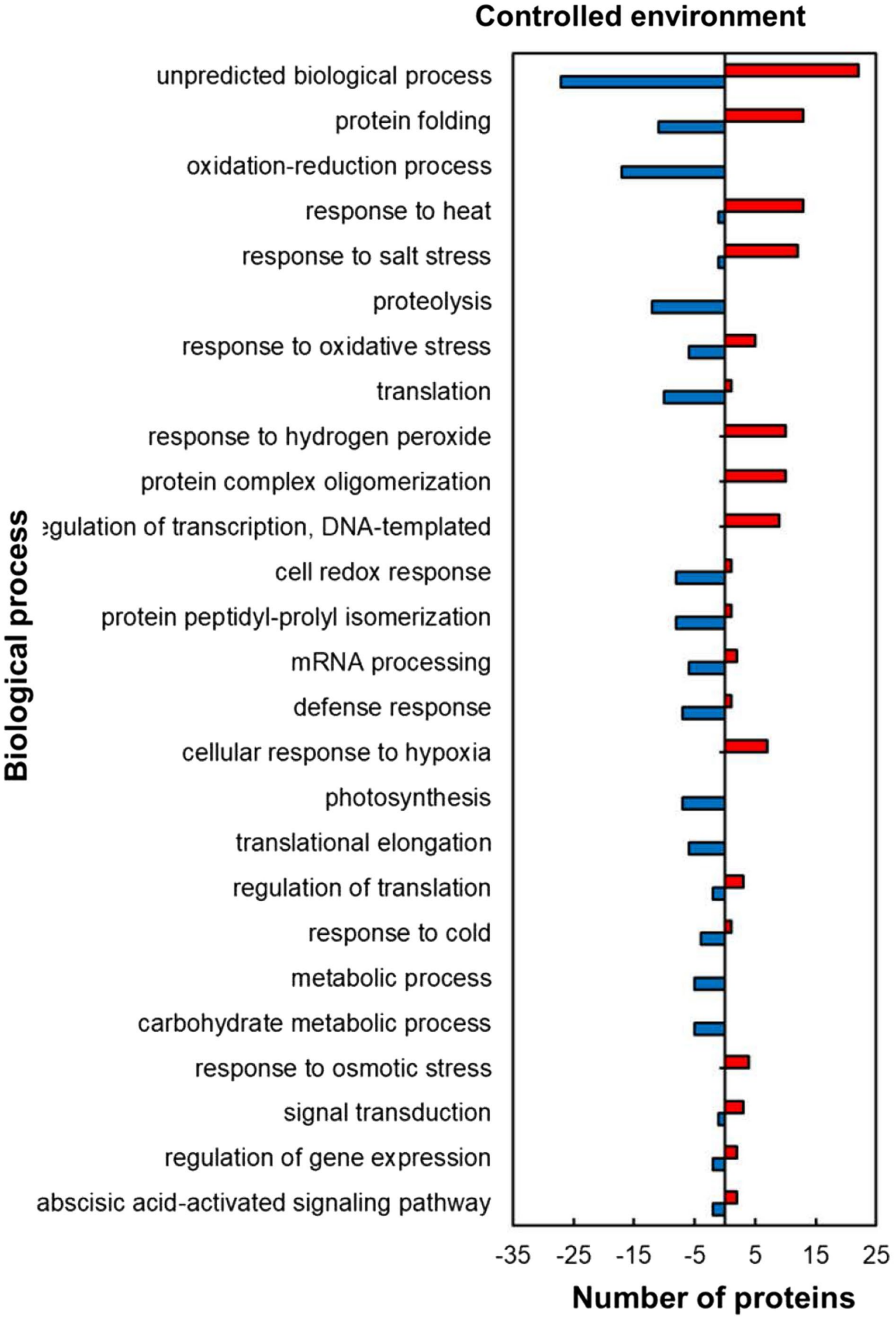
Biological process prediction of heat upregulated and downregulated proteins from IT-96D-610 from heat-stressed plants relative to controls in the controlled environment based on GO annotation. Positive numbers (red) represent number of upregulated proteins and negative numbers (blue) the number of downregulated proteins.

Within the predicted biological processes GO category, the plants responded to heat stress in the controlled environment by upregulating proteins related to stress protection and RNA synthesis while downregulating mostly those related to protein and carbohydrate metabolism pathways (Figure 4). Several stress-protective proteins were upregulated including those associated with heat stress (13; 93%), salt stress (12; 92%), response to hydrogen peroxide (10; 100%), cellular response to hypoxia (7; 100%) and response to osmotic stress (4; 100%; Figure 4). Other heat upregulated proteins related to RNA synthesis were in the regulation of transcription, DNA-templated (9; 100%) and those related to protein folding (13; 54%) and protein complex oligomerization (10; 100%). In contrast to the stress protection and RNA synthesis, proteins related to protein processing and synthesis were mostly downregulated including those involved in proteolysis (12; 100%), protein peptidyl-prolyl isomerization (8; 89%), and translation (10; 91%) during stress exposure. HSRPs with roles related to carbohydrate synthesis processes were mostly downregulated in photosynthesis (7; 100%) and carbohydrate metabolic processes (5; 100%). Other important proteins that were downregulated include the oxidative stress-protective proteins involved in the cell redox response (8; 89%) and response to oxidative stress (6; 55%).

**Figure 5:**
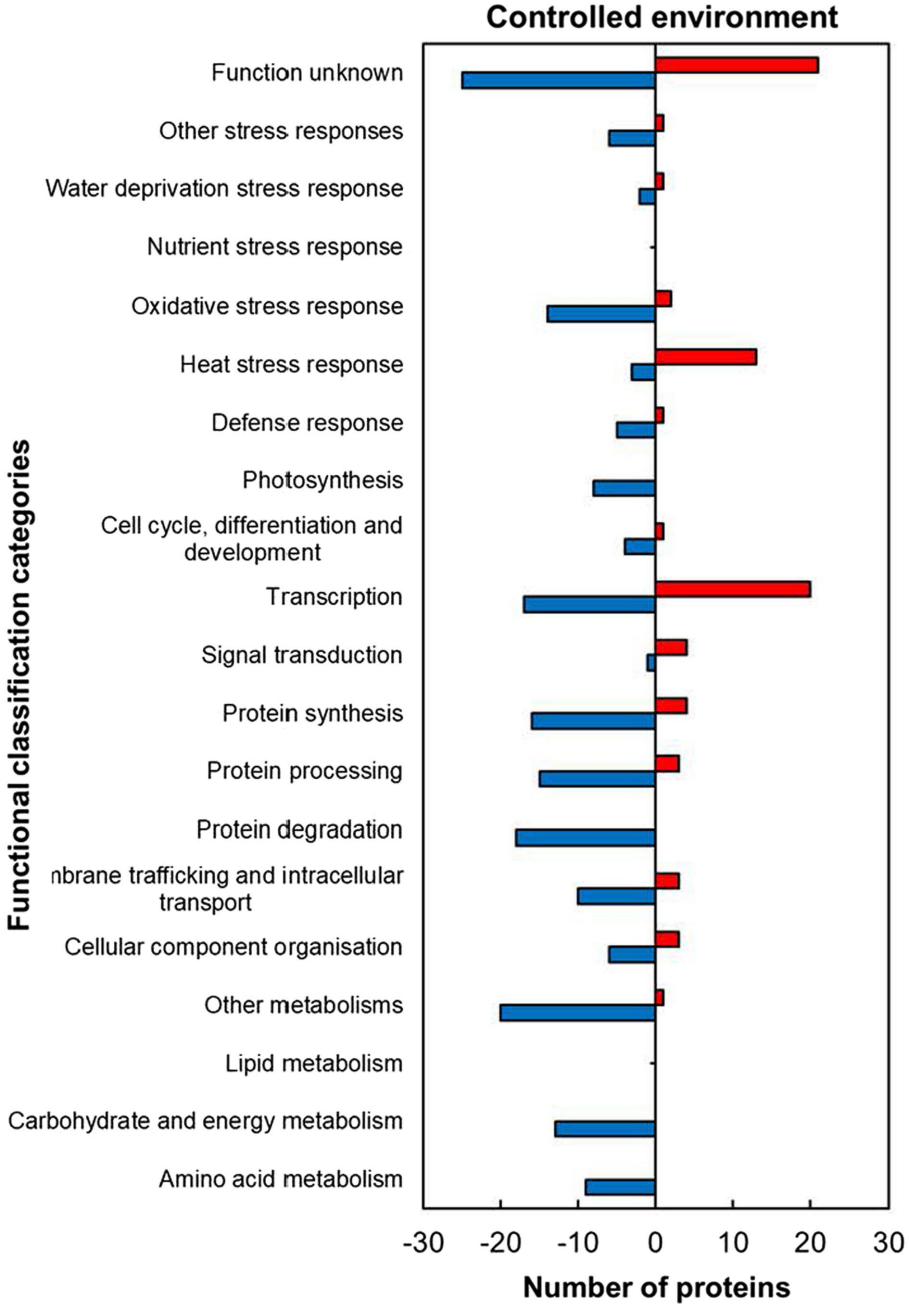
Abundance of heat upregulated and downregulated proteins from heat-stressed plants relative to controls in the controlled environment organized through functional classification from biological process GO categories. Positive numbers (red) represent number of upregulated proteins and negative numbers (blue) the number of downregulated proteins.

The analysis of functional classification categories indicated that heat stress-responsive proteins (HSRPs) mostly involved in molecular and biological processes as well as metabolic pathways were downregulated in heat-stressed compared to control plants (Figure 5). The downregulated proteins included most of those involved in molecular processes such as membrane trafficking and intracellular transport (10; 77%), cellular component organisation (6; 67%), protein synthesis (16; 80%), processing (15; 83.3%) and degradation (18; 100%). Metabolic pathways and biological processes with the majority of HSRPs downregulated included amino acid (9; 100%) and other metabolic pathways (20, 95%), and in carbohydrate and energy metabolism (13; 100%) as well as photosynthesis (8; 100%). Furthermore, the majority of HSRPs involved in one stress protection response of oxidative stress response (14; 87.5%) were also downregulated (Figure 5). However, proteins involved in heat stress response (13; 81%), transcription (20; 54%) and signal transduction (4; 80%) had the majority of the HSRPs upregulated (Figure 5). Some of the proteins in heat stress response included 17.6 kDa class II heat shock protein, 17.6 kDa class I heat shock protein 1-related and BAG family molecular chaperone regulator 6 (Supplementary Table S1).

### Proteomic response of IT-96D-610 plants to heat stress in the field

**Figure 6:**
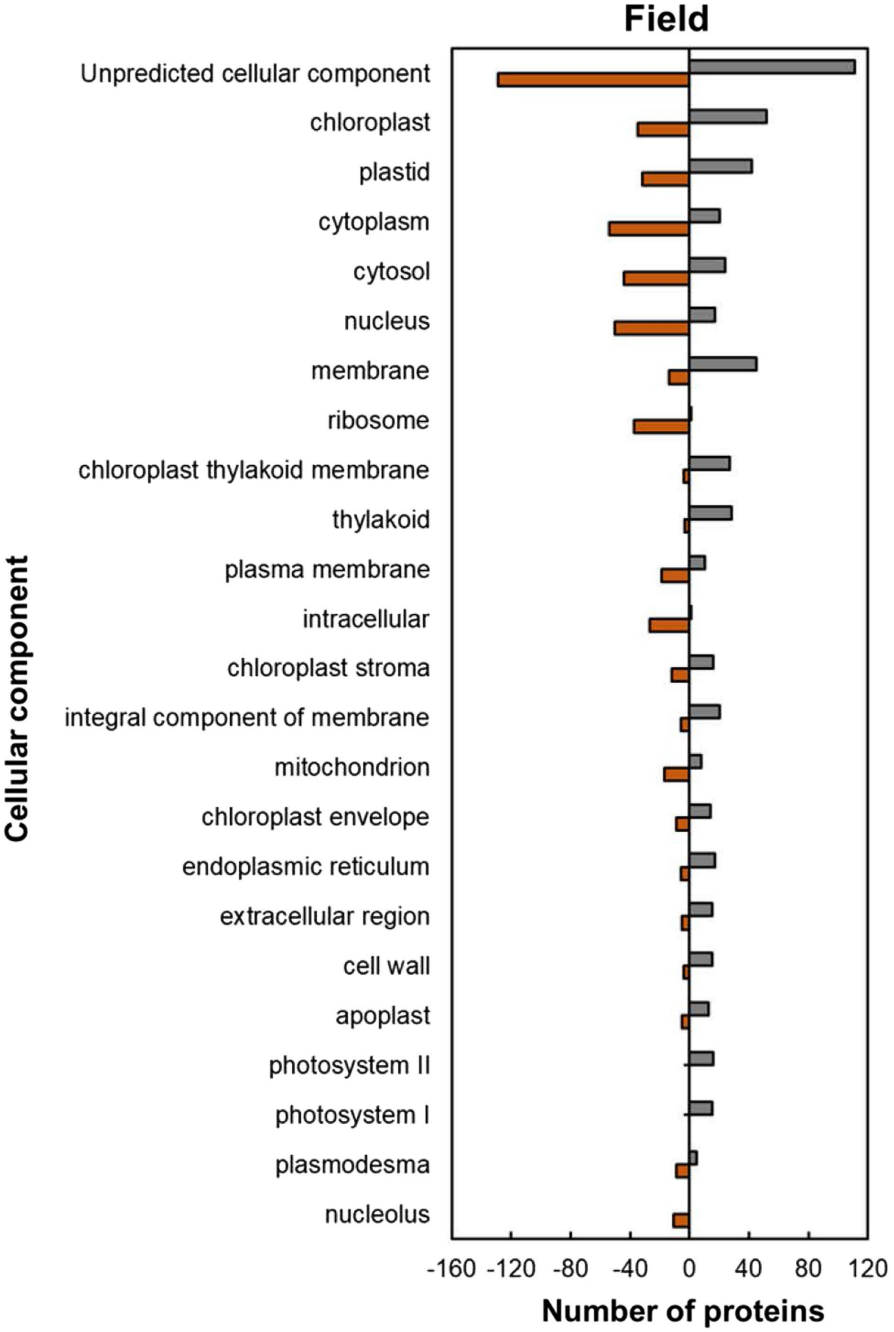
Cellular component predictions of the heat upregulated and downregulated proteins from heat-stressed plants at Marapyane relative to plants at Eensaamheid in the field based on GO annotation. Positive numbers (green) represent number of upregulated proteins and negative numbers (brown) the number of downregulated proteins.

Within the predicted cellular component GO categories, the majority of HSRPs were upregulated in the intracellular and extracellular components of heat-stressed IT-96D-610 plants at the hot Marapyane site relative to those at the cool Eensaamheid (Figure 6). On the other hand, the HSRPs were downregulated in the cellular boundary components and cellular fluids at Marapyane relative to Eensaamheid (Figure 6). The intracellular and extracellular components that were upregulated in the stressed plants included chloroplast (52; 60%), endoplasmic reticulum (17; 74%), extracellular region (15; 75%), cell wall (15; 79%) and apoplast (13; 72%) proteins. Whereas the downregulated HSRPs localized in the intracellular fluid included cytoplasm (54; 73%) and cytosol (44; 65%), while the boundary component was plasma membrane (19; 66%). Furthermore, many nucleus proteins (50; 75%) and mitochondrion proteins (17; 68%) constituted HSRPs downregulated in the intracellular components.

**Figure 7:**
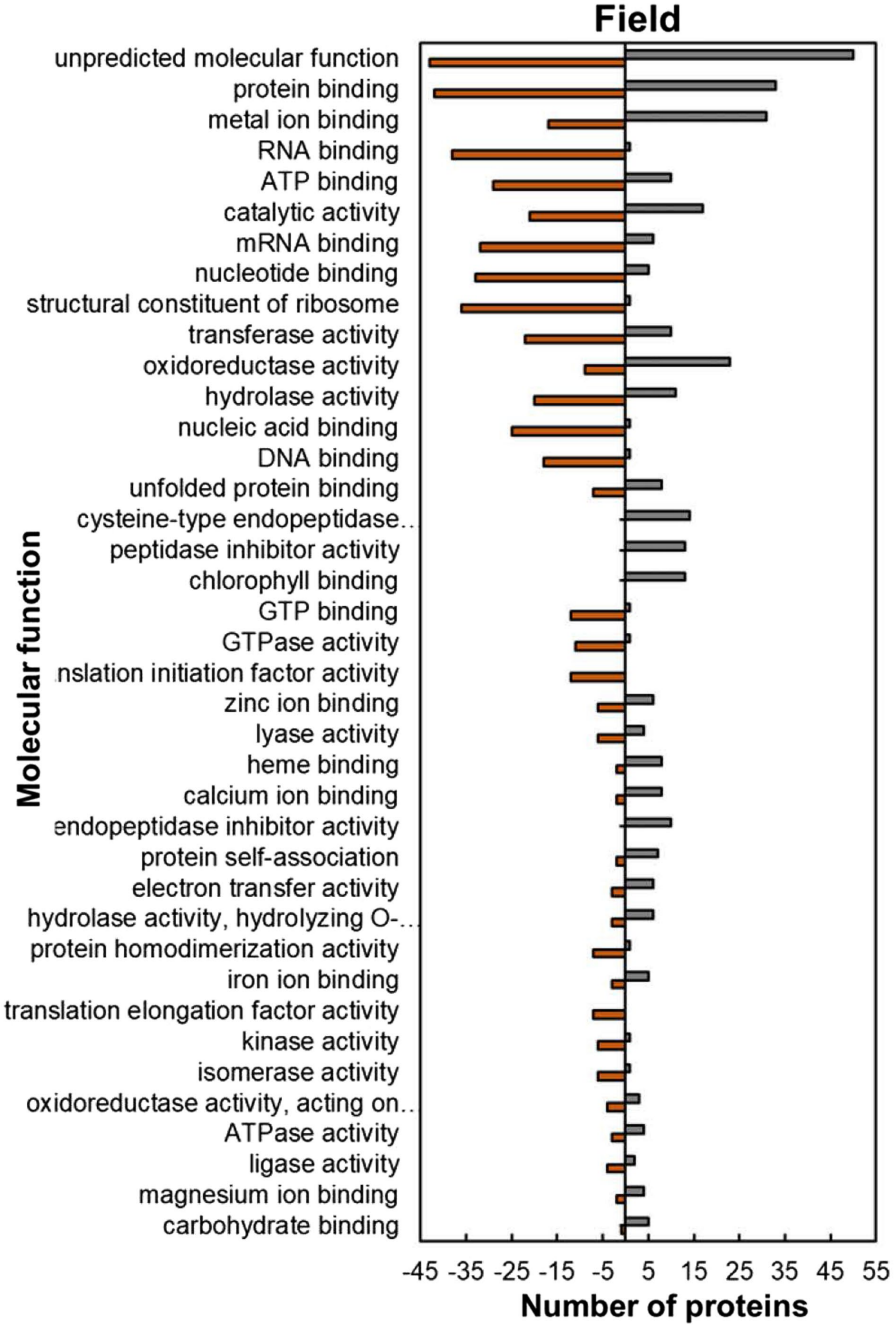
Molecular function predictions of the heat upregulated and downregulated proteins from heat-stressed plants at Marapyane relative to plants at Eensaamheid in the field based on GO annotation. Positive numbers (green) represent number of upregulated proteins and negative numbers (brown) the number of downregulated proteins.

Within the predicted molecular function GO category, most of the HSRPs which were upregulated had functions of protein and metal ion binding such as unfolded protein binding (8; 53%), protein self-association (7; 77%), iron ion binding (5; 63%), magnesium ion binding (4; 67%) and calcium ion binding (8; 80%; Figure 7). In contrast, HSRPs which were mostly downregulated had functions of nucleic acid or nucleotide-binding such as RNA binding (38; 97%), mRNA binding (32; 84%), ATP binding (29; 74%), DNA binding (18; 95%), and GTP binding (12; 92%; Figure 7).

**Figure 8:**
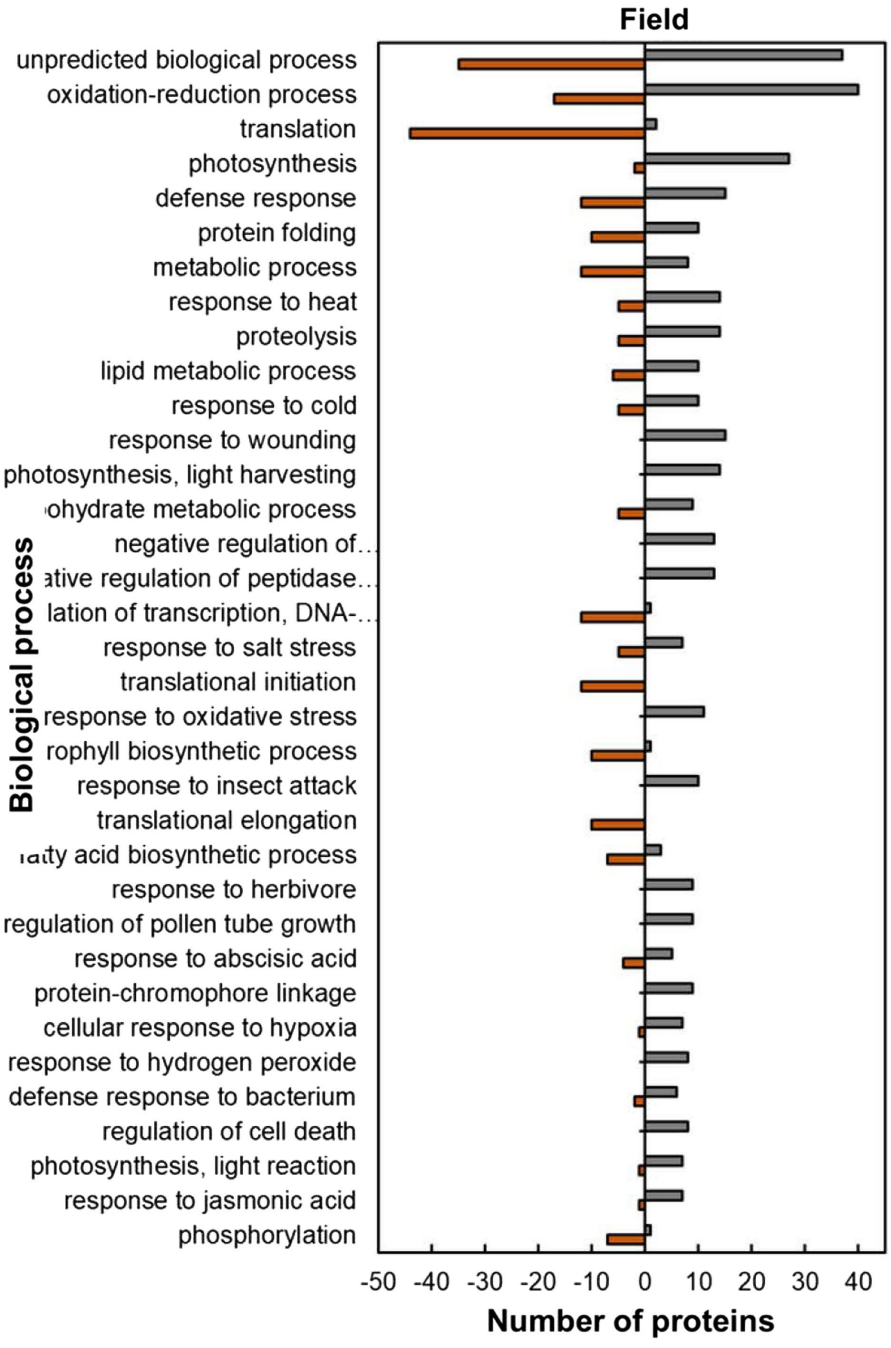
Biological process predictions of the heat upregulated and downregulated proteins from heat-stressed plants at Marapyane relative to plants at Eensaamheid in the field based on GO annotation. Positive numbers (green) represent number of upregulated proteins and negative numbers (brown) the number of downregulated proteins.

The analysis of predicted biological process GO categories showed that heat-stressed plants upregulated the stress protection response and metabolic pathways but downregulated molecular processes (Figure 8). The stress protection response proteins that were upregulated included those involved in response to heat (14; 74%), such as small heat shock proteins HSP17.6I, HSP17.6II and HSP22 and heat shock binding protein (Figure 8; Supplementary Table S2). Other stress-protective proteins that were mostly upregulated are linked to defence response (15; 56%), response to wounding (15; 100%), response to oxidative stress (11; 100%), response to insect attack (10; 100%), response to cold (10, 67%) and response to salt stress (7; 58%). Other biological process proteins that were also upregulated were involved in photosynthesis (27; 93%), proteolysis (14; 74%), lipid metabolic process (10; 63%), carbohydrate metabolic process (9; 64%) and negative regulation of endopeptidase (13; 100%). In contrast, the biological processes with majority of HSRPs downregulated were translation (44; 96%), regulation of transcription, DNA-templated (12; 92%), translation initiation (12; 100%), translation elongation (10; 100%) and the chlorophyll biosynthetic process (10; 91%).

**Figure 9:**
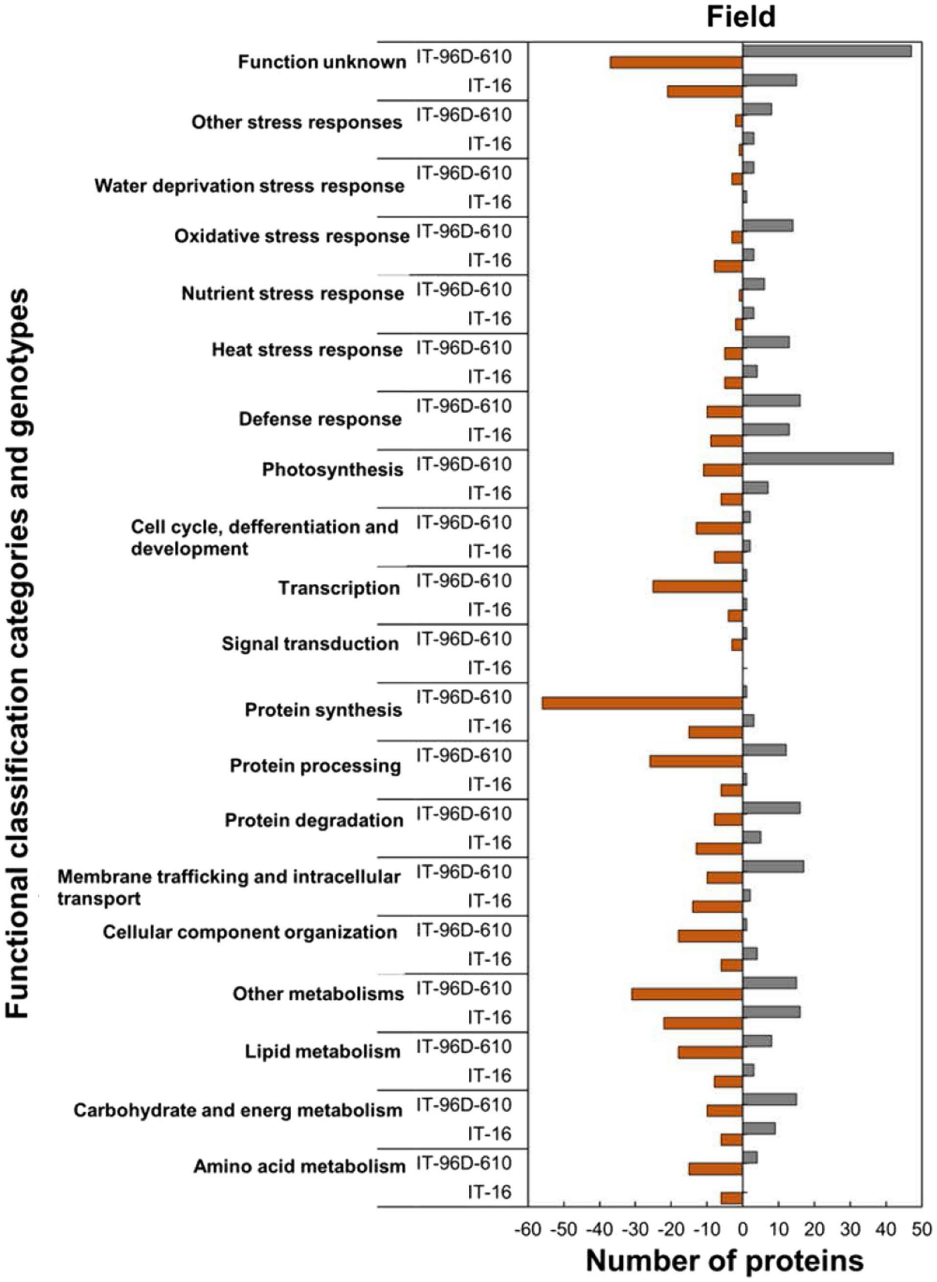
Abundance of the heat upregulated and downregulated proteins from heat-stressed plants at Marapyane relative to plants at Eensaamheid in the field in three genotypes in two IT-96D-610, and IT-16 organized through functional classification from biological process GO categories. Positive numbers (green) represent number of upregulated proteins and negative numbers (brown) the number of downregulated proteins.

Analysis of the functional classification categories showed that heat-stressed plants upregulated stress protection response while proteins for molecular processes and metabolic pathways were downregulated (Figure 9). Stress protection responses such as defence response (16; 62%), heat stress response (13; 72%), nutrient response (6; 86%) and oxidative stress response (3; 60%) had the majority of HSRPs upregulated (Figure 9). Examples of the heat stress response proteins were molecular chaperone DNAJ, heat shock protein 101 and aldehyde dehydrogenase 5F1 (Supplementary Table S2). Furthermore, molecular processes and metabolic pathways including membrane trafficking and intracellular transport (17; 63%), protein degradation (16; 67%) and carbohydrate and energy metabolism (15; 60%) had the majority of HSRPs upregulated. However, stressed plants downregulated the majority of the HSRPs involved in other molecular processes and metabolic pathways such as transcription (25; 96%) and signal transduction (3; 75%).

### Comparison of proteomic responses of IT-96D-610 with IT-16 genotypes to heat stress in the field

In the field trials using the two genotypes, a comparison of HSRPs at the hot and drier Marapyane site with the cooler and wetter site at Eensaamheid showed that IT-96D-610 had the largest number (242; 44%) of stress upregulated proteins compared to IT-16 which had (94; 37%; Supplementary Tables S2, S3). Furthermore, IT-96D-610 also had more downregulated proteins (305; 56%) than IT-16 with (161; 63%). The highest fold change range of proteins was from IT-96D-610 which had a range of 2.0 to 106.6 for heat upregulated proteins and − 2.0 to −264.8 for heat downregulated proteins. In contrast to IT-96D-610 genotype, IT-16 had a range of 2.0 to 34.4 for stress upregulated proteins and − 2.0 to 12.1 for downregulated proteins.

**Figure 10:**
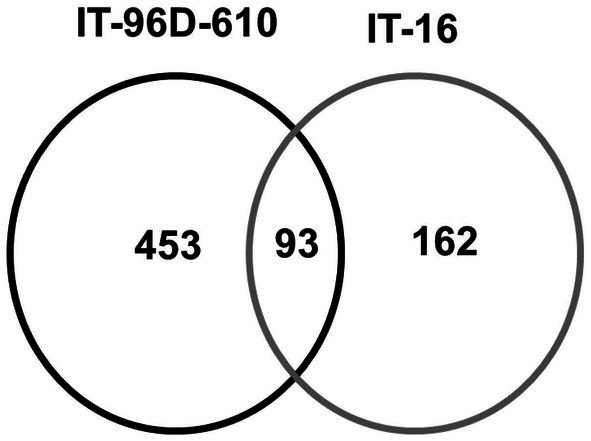
The comparison of identified heat regulated proteins in two genotypes IT-96D-610 and IT-16 from the field experiment.

A total of 3,537 proteins were identified for the two genotypes in the field and only 801 were heat-stress-regulated proteins. Among the stress-regulated proteins, IT-96D-610 recorded the largest number of 546 while IT-16 only had 255. Genotype IT-96D-610 also had the largest number (453) of unique proteins, followed by IT-16 (162; Figure 10). Proteins that were found in both genotypes were 93 and represented 17% in IT-96D-610 and 36% in IT-16.

Within functionally classified categories from the two genotypes, the stress protection responses and some molecular process proteins were upregulated in genotype IT-96D-610 in the field in heat-stressed plants relative to the control plants and these were absent in IT-16 (Figure 9). The majority of HSRPs involved in the molecular process and stress protection response such as protein degradation and heat stress response from genotype IT-96D-610 (16; 67% and 13; 72%, respectively) were mostly upregulated but were downregulated in IT-16 (13; 72% and 5; 56%). Furthermore, the majority of HSRPs involved in other molecular processes and stress protection responses such as membrane trafficking and intracellular transport and oxidative stress response from IT-96D-610 (17; 63% and 14; 82%, respectively) were upregulated while those from IT-16 (14; 88% and 8; 73%, respectively) were mostly downregulated (Figure 9).

**Figure 11:**
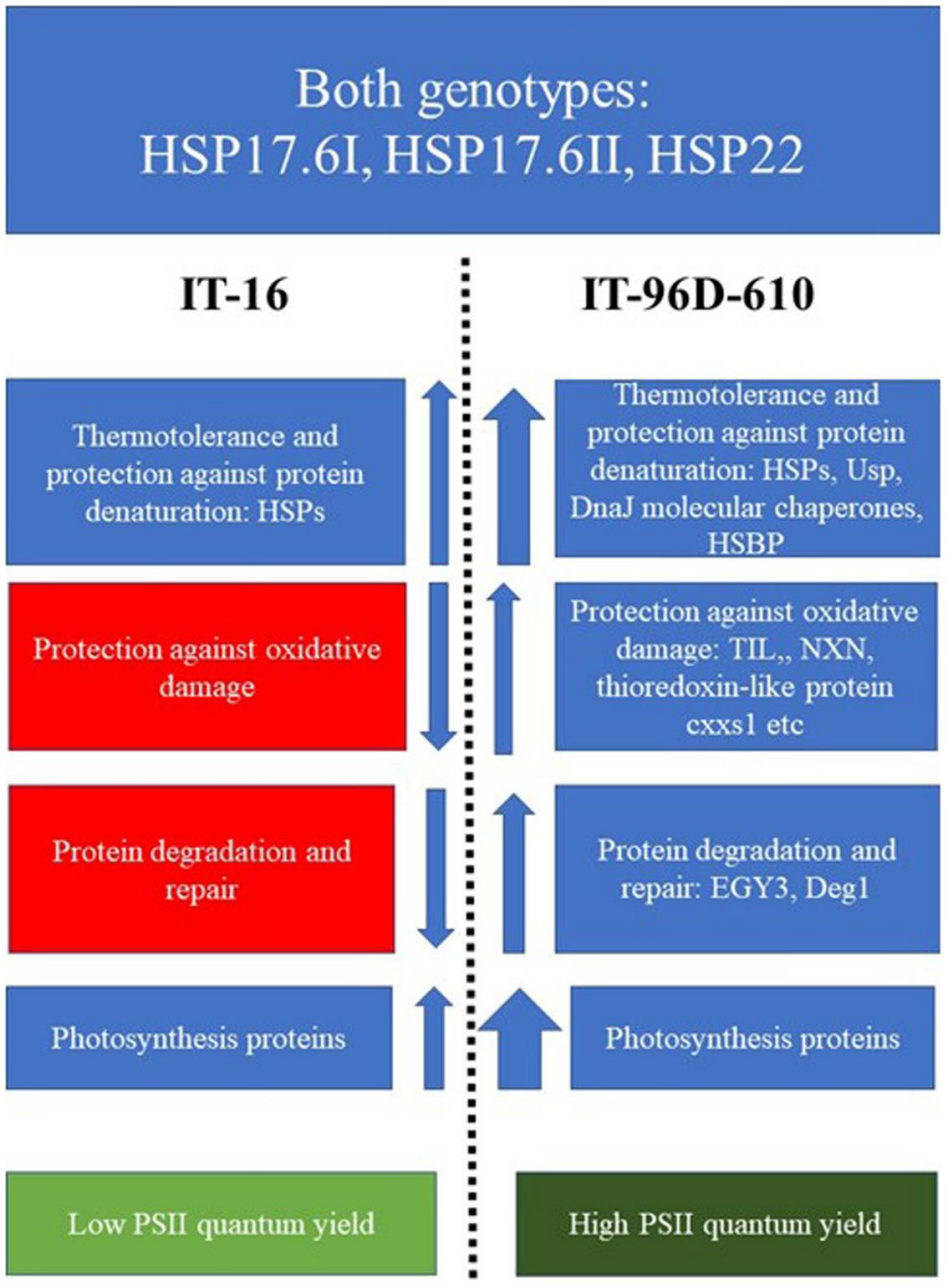
A scheme highlighting the functions and up- or down-regulation (arrows) of proteins that were expressed in IT-16 and IT-96D-610 genotypes in the field and their chlorophyll fluorescence response. Red boxes highlight the downregulated proteins, the blue highlight the upregulated proteins and green boxes highlight the chlorophyll fluorescence response. The width of the arrow depicts the strength of the response.

A schematic diagram showing the differences in the up- or down-regulation of proteins in the two genotypes exposed to heat stress in the field showed that a wider variety of proteins variety of HSPs, chaperones and photosynthetic proteins were upregulation more in IT-96D-610 than in TI-16 (Figure 11). Furthermore, proteins associated with protection against oxidative damage and protein degradation and repair were upregulated in IT-96D-610 whereas they were downregulated in TI-16.

## Discussion

### Proteome response of IT-96D-610 under controlled conditions

The heat-stressed IT-96D-610 plants in the controlled environment upregulated HSPs from the heat stress response category (Supplementary Table S1; Figure 5) including 17.6 kDa class I (HSP17.6I), 17.6 kDa class II (HSP17.6II) and HSP22 that are recognized for thermotolerance by offering protection to sensitive proteins (Dafny-Yelin et al., 2008; Sarkar et al., 2020). The high proportion of upregulated proteins in the cytoplasm (Figure 2) including HSP17.6I and HSP17.6II are involved in folding denatured or partially folded proteins and ensuring proper aggregation of heat-sensitive proteins in the cytosol (McLoughlin et al., 2016; Jiang et al., 2017). The HSP22 modulates polar auxin transport and hypocotyl elongation activated by auxin in *A. thaliana* (Li et al., 2018). Consistently, a high number of proteins with molecular functions of protein binding, protein folding as well as unfolded protein binding was upregulated (Figure 3) suggesting similar functions as HSPs.

Other proteins from the heat stress response category that were upregulated in the controlled environment had a chaperone function including BAG family molecular chaperone regulator 6 (BAG6), Multiprotein bridging factor 1C (MBF1C) and cold shock domain protein 1 (CSDP1; Supplementary Table S1). The BAG6 protein has been shown to improve basal thermotolerance response (Echevarría-Zomeño et al., 2016). It binds to calcium calmodulin and is a co-chaperone of HSP70 which refolds heat-sensitive proteins (Kang et al., 2006; Mayer, 2013; Fu et al., 2019). During heat stress, BAG6 assists HSP70 in folding the denatured proteins (Doukhanina et al., 2006; Fu et al., 2019). In addition, the MBF1C proteins activated transcriptional expression of DRE-binding protein2A, heat shock transcription factors and zinc finger proteins and were regarded as key regulators of thermotolerance response in *A. thaliana* (Suzuki et al., 2011). This is consistent with the results of the current study with the upregulation of proteins involved in transcription (Figure 5), specifically the upregulation of transcription factors (WRKY transcription factor-20-related, transcription factor POSF21-related, sterol regulatory element-binding protein // transcription factor BIM1) and zinc finger proteins (zinc finger CCCH domain-containing protein 36-related and CCCH-type zinc finger family protein) which were possible candidates for transcriptional activation by MBF1C (Supplementary Table S1). The controlled environment plants upregulated transcription proteins regulated by calcium signalling [VirE2-interacting protein 1 (VIP1)], those that are involved in stomatal closure (WRKY20 and WRKY transcription factor 20-related proteins) and possibly thermotolerance through circadian clock response (ELF5; Noh et al., 2004; Luo et al., 2013; Liu et al., 2015). Cold shock domain protein 1 (CSDP1) is an RNA chaperone that has been shown to protect *A. thaliana* plants from low-temperature stress through modulating RNA metabolism (Park et al., 2009). Its upregulation in heat-stressed plants implies that it might also be involved in heat tolerance.

Disease resistance protein (RAR1) upregulated in heat-stressed plants (Supplementary Table S1). RAR1 is a co-chaperone protein that forms a stable complex with HSP90 and SGT1 (Shirasu, 2009; Wang et al., 2016) to stabilize an auxin signalling co-receptor protein TIR at temperatures of 29°C and prevents its degradation (Wang et al., 2016). This induces auxin-dependent processes such as hypocotyl and root growth in *A. thaliana* seedlings (Wang et al., 2016; Tichá et al., 2020). RAR1 is also essential for the functioning of disease resistance and stabilizes resistance (R) proteins thereby contributing to pathogen effector recognition (Shirasu, 2009). Therefore, RAR1 may be a general responsive protein induced in response to both biotic and abiotic stresses. Two plasminogen activator inhibitor 1 RNA-binding (SERBP1) proteins (Vigun05g048200.1.p and Vigun10g009600.1.p) were also signalling proteins upregulated in heat-stressed plants (Supplementary Table S1). Both proteins are orthologues of RGG repeats nuclear RNA binding protein A (RGGA), a cytoplasmic protein that is expressed in stomata in *A. thaliana* for stomata closure (Ambrosone et al., 2015). It has been shown that RGGA is involved in ABA-dependent stomatal closure in *A. thaliana* to adapt to drought stress (Ambrosone et al., 2015). These ABA-responsive proteins in the plants were possibly upregulated in heat-stressed plants because the plants experienced drought-induced stress because of high temperatures in the controlled environment where plants were watered only once a day. This could also be the reason for the upregulation of HSRPs involved in osmotic stress response and salt stress response proteins (Figure 3).

### Cowpea proteome response under field conditions and relative to controlled environment

Similar to the controlled environment results, the heat-stressed IT-96D-610 plants in the field (hotter Marapyane site) also upregulated small heat shock proteins HSP17.6I, HSP17.6II and HSP22 (Supplementary Table S2) for the protection of sensitive proteins (McLoughlin et al., 2016; Jiang et al., 2017). The upregulation of these heat shock proteins was a general heat stress response (Echevarría-Zomeño et al., 2016) of the IT-96D-610 genotype. Therefore, the implication is that these HSPs are relevant to the thermotolerance of cowpea in the field and can be explored further for trait association with physiological traits.

In the current study, the heat-stressed IT-96D-610 genotype in the field produced more types of heat shock proteins including HSP101 than the controlled environment plants (Supplementary Table S2). Heat shock protein 101 interacts with both classes of small heat shock proteins to protect them and the proteins they interact with from irreversible aggregation during heat stress (McLoughlin et al., 2016). The universal stress protein family (Usp) was also upregulated in heat-stressed plants in the field (Supplementary Table S2). The Usp protein changes its structure during heat stress to attain a chaperone function in which it protects other proteins and RNAs from denaturation (Chi et al., 2019). Another set of proteins with a chaperon function is the DnaJ molecular chaperones (molecular chaperone DnaJ and chaperone DnaJ-domain superfamily protein) that are involved in the protection of PSII against reactive oxygen species (ROS) damage by preventing denaturation of APX and SOD proteins during heat stress (Kong et al., 2014). Furthermore, heat shock factor binding protein (HSBP), a molecular chaperone, was also upregulated in heat-stressed plants (Supplementary Table S2) and is proposed to contribute to thermotolerance. Other unique stress-protective proteins within the heat stress response category such as temperature-induced lipocalin (TIL) and Aldehyde dehydrogenase 5F1 (also referred to as succinic-semialdehyde dehydrogenase) upregulated only in the field are involved in the protection against temperature-induced oxidative stress damage (Boca et al., 2014; Tola et al., 2021). Therefore, the upregulation of TIL and succinic semialdehyde dehydrogenase in the field is part of the specialized response for plant protection against oxidative stress caused by heat stress (Echevarría-Zomeño et al., 2016). Proteins involved in the degradation of other proteins (i.e., proteases) such as Zinc metallopeptidase EGY3, chloroplastic-related and DegP protease 1 (Deg1) were upregulated in heat-stressed plants only in the field (Supplementary Tables S1, S2) to repair heat stress damaged proteins (Adamiec et al., 2020). The EGY3 protein is localized in chloroplasts and induced by HSFA1b and H_2_O_2_ signalling during thermotolerance response in *A. thaliana* (Bechtold et al., 2013; Adamiec et al., 2020). Adamiec et al. (2020) proposed that during photo-inhibition induced by irradiation, EGY3 protein levels are associated with those of Deg1 and FtsH2/8 proteases which function in the repair of photodamaged PSII complex proteins in *egy3 A. thaliana* mutants lacking EGY3 protein. This indicates that both EGY3 and Deg1 have a related response to protect against heat stress-induced photo-inhibition. Therefore, the wider variety of HSPs (e.g., HSP101), chaperones and non-chaperone proteins found in the IT-96D-610 genotype when grown at the hot Marapyane site than in the controlled environment indicate that the field environment is more conducive to upregulation of thermotolerance proteins. The reasons for this could be that the plants in the controlled environment were exposed to temperatures that were much higher for a shorter period than in the field even though both were compared at the flowering stage. It has been shown that heat stress duration has a major impact on the induction of heat stress-responsive proteins resulting in the death or survival of the *A. thaliana* plants in controlled environments (Echevarría-Zomeño et al., 2016). In agreement to this report, the current study showed that the plants in the field with moderately hot temperatures (35°C) had more differentially regulated proteins (547 proteins) than in the controlled environment (270 proteins) with high temperature (40°C; Figure 1).

Notably, the non-HSP proteins upregulated in the controlled environment (BAG6, MBF1C and CSDP1), were different to those upregulated in the field and included universal stress protein (Usp), DnaJ chaperones and heat shock factor binding protein (HSBP) indicating specialized to upregulation in the different environment (Echevarría-Zomeño et al., 2016). This suggests that the type of environmental growing conditions and the severity of the heat stress have a major impact on which heat stress-responsive proteins are induced and involved in thermotolerance. Furthermore, signalling transduction and transcription proteins in IT-96D-610 were upregulated by heat stress in the controlled environment but downregulated in the field (Figure 1). Unique proteins involved in oxidative stress response were upregulated in the field (Figure 9) to restore redox homeostasis by protecting the plant against ROS damage and inducing senescence (Serrato et al., 2008; Fernández-Pérez et al., 2015; Kapoor et al., 2015). These upregulated proteins were involved in ROS detoxification (peroxidase 52), lipid protection against ROS damage (tocopherol cyclase, chloroplastic lipocalin, glutaredoxin), repair of proteins from ROS damage [WCRKC thioredoxin 2, nucleoredoxin (NXN), thioredoxin//thioredoxin-like protein cxxs1] and induction of senescence through the effect on ROS homeostasis [thiosulfate sulfurtransferase/Thiosulfate thiotransferase (alternatively Senescence 1, SEN1); Vieira Dos Santos and Rey, 2006; Serrato et al., 2008; Fernández-Pérez et al., 2015; Kapoor et al., 2015]. The down-regulation of the ROS protective proteins in the controlled environment further highlights the distinction between short-term heat stress exposure in the controlled environment and the long-term heat stress in the field (Figures 5, 9). However, the mechanisms for the up- or down-regulation of some of the proteins in plants in the field relative to controlled conditions is unclear.

### Responses of the two genotypes to heat stress in the field

Genotype IT-96D-610 had a higher number and wider variety of upregulated proteins in the heat response category including small heat shock proteins than IT-16 (Figures 9, 11; Supplementary Tables S2, S3) suggesting that IT-96D-610 had improved protection of proteins from heat stress-induced denaturation (McLoughlin et al., 2016; Jiang et al., 2017). IT-96D-610 had many proteins that were upregulated and involved in the repair of PSII machinery (see Figures 9, 11; Supplementary Table S3) which is also reflected in the higher number of proteins involved in photosynthesis (Figure 9) compared to IT-16. DegP protease 1 is one of the proteins involved in the repair of PSII machinery and chloroplastic lipocalin is involved in protection against oxidative damage in the chloroplast (Boca et al., 2014; Malnoë et al., 2018; Adamiec et al., 2020), therefore indicating that IT-96D-610 may also have had better repair of PSII machinery and protection against oxidative stress than IT-16. This was also supported by a change in the maximum operating efficiency of PSII in IT-16 that was lower at the hotter Marapyane field site than at the cooler Eensaamheid field site while PSII efficiency of IT-96D-610 was unchanged at both sites (data not shown).

## Conclusion

Plants of genotype IT-96D-610 in the controlled environment upregulated small HSPs including 17.6 kDa class I (HSP17.6I), 17.6 kDa class II (HSP17.6II) and HSP22 and other chaperones such as BAG6, MBF1C and CSDP1 as well as signalling proteins involved in drought (RGGA) tolerance and transcription proteins. The IT-96D-610 plants in the field upregulated a wider range of small HSPs including those identified in the controlled environment and unique HSP (HSP101), chaperones (Usp protein, DnaJ) and other non-HSP proteins (TIL and succinic dehydrogenase, EGY3, Deg1 and ROS protective proteins). IT-96D-610 had better thermotolerance than IT-16 due to a more abundant and wider variety of heat shock proteins, and proteins involved in protein degradation and PSII repair, and oxidative stress response. It had higher expression of proteins involved in protection of proteins involved in photosynthesis resulting in a higher maximum operating efficiency of PSII than IT-16. The genotype also expressed two types of proteins that are involved in transcription and signalling associated with regulation of stomatal closure. This is the first study that unravels a proteomic response to heat stress in cowpea and showed thermotolerance proteomic mechanisms. It also showed the different proteomic responses that occur in the controlled and field environments which have received limited attention in heat stress response literature. The wide variety of HSPs, chaperones and non-chaperon proteins observed in this study provides insights into the proteomic response to heat stress and thermotolerance mechanisms of cowpea grown in controlled and field environments.

## Data availability statement

The data presented in this study are deposited in the ProteomeXChange database, accession number PXD035418.

## Author contributions

TS contributed to the design of the study, experimentation, acquisition of data, analysis and interpretation of data, and drafting the article. SC conceptualized the project and obtained funding. SC and MR designed and administered the experiment. SC, SM, HG, MR, AM, OC, JO, AV, C-OO, and ER contributed to supervision, methodology, presentation of results, critical revision of article, and editing. All authors contributed to the article and approved the submitted version.

## Funding

The study was funded by the University of Cape Town and the National Research Foundation—South Africa, grant no: 98862.

## Conflict of interest

The authors declare that the research was conducted in the absence of any commercial or financial relationships that could be construed as a potential conflict of interest.

## Publisher’s note

All claims expressed in this article are solely those of the authors and do not necessarily represent those of their affiliated organizations, or those of the publisher, the editors and the reviewers. Any product that may be evaluated in this article, or claim that may be made by its manufacturer, is not guaranteed or endorsed by the publisher.
