# Supplementary material for: Regulation and physiological function of proteins for heat tolerance in cowpea (Vigna unguiculata) genotypes under controlled and field conditions
Source: Front Plant Sci. 2022 Aug 22;13:954527. doi: 10.3389/fpls.2022.954527 (PMC9441852; doi:10.3389/fpls.2022.954527)
Supplement: Supplementary file 1 [file Table_1.DOCX]

Table 1: List of responsive proteins regulated in stressful temperatures in the phytotron compared to in the cooler phytotron from IT-96D-610 using the label free quantification and database searches.

| Regulation | Accession | Protein name | **Species from which the protein or orthologue was obtained** | Peptide count | Unique peptide | | Confidence score | q Value | Max fold change |
| --- | --- | --- | --- | --- | --- | --- | --- | --- | --- |
|  | **Amino acid metabolism** | |  |  | |  |  |  |  |
| Down | Vigun01g228600.6.p | phosphoribosylanthranilate isomerase 1 | *Vigna unguiculata* | 2 | | 2 | 18.95 | 0.015 | 11.559 |
| Down | Vigun06g165200.1.p | Dihydroxy-acid dehydratase | *Vigna unguiculata* | 4 | | 4 | 22.398 | 0.045 | 5.279 |
| Down | Vigun07g129400.1.p | S-adenosylmethionine synthetase 2 | *Vigna unguiculata* | 4 | | 4 | 24.396 | 0.002 | 16.864 |
| Down | Vigun07g202400.1.p | Chorismate synthase / 5-enolpyruvylshikimate-3-phosphate phospholyase | *Vigna unguiculata* | 3 | | 3 | 9.554 | 0.010 | 4.101 |
| Down | Vigun08g050500.1.p | aspartate aminotransferase 5 | *Vigna unguiculata* | 4 | | 2 | 12.551 | 0.029 | 3.4371 |
| Down | Vigun06g160500.2.p | aminomethyltransferase (gcvT, AMT) | *Vigna unguiculata* | 17 | | 15 | 130.66 | 0.029 | 5.057 |
| Down | Vigun03g009600.1.p | aspartate-semialdehyde dehydrogenase (asd) | *Vigna unguiculata* | 8 | | 5 | 40.569 | 0.010 | 4.572 |
| Down | Vigun02g156500.1.p | glycine decarboxylase complex H | *Vigna unguiculata* | 13 | | 13 | 208.887 | 0.029 | 2.014 |
| Down | Vigun01g156400.1.p | Uncharacterized protein | *Vigna unguiculata* | 2 | | 2 | 21.10 | 0.015 | 5.1112 |
|  | **Carbohydrate and energy metabolism** | |  |  | |  |  |  |  |
| Down | Vigun02g043200.1.p | Transketolase | *Arabidopsis thaliana* | 13 | | 3 | 87.410 | 0.032 | 29.732 |
| Down | Vigun07g287700.1.p | Pectate lyase / PPase-N | *Arabidopsis thaliana* | 5 | | 4 | 15.061 | 0.046 | 3.681 |
| Down | Vigun07g206600.1.p | pyruvate dehydrogenase E2 component (dihydrolipoamide acetyltransferase) (DLAT, aceF, pdhC) | *Arabidopsis thaliana* | 2 | | 2 | 14.495 | 0.010 | 12.158 |
| Down | Vigun07g072100.1.p | SNF1 PROTEIN KINASE SUBUNIT BETA-2-RELATED | *Arabidopsis thaliana* | 3 | | 3 | 14.024 | 0.029 | 5.313 |
| Down | Vigun01g053500.1.p | phosphoglycerate kinase 1 | *Vigna unguiculata* | 32 | | 27 | 272.885 | 0.026 | 3.741 |
| Down | Vigun01g171100.1.p | starch branching enzyme 2.2 | *Vigna unguiculata* | 2 | | 2 | 10.167 | 0.012 | 14.307 |
| Down | Vigun03g015200.3.p | PHOSPHOHEXOMUTASE FAMILY MEMBER // PHOSPHOGLUCOMUTASE, CYTOPLASMIC 1-RELATED | *Vigna unguiculata* | 3 | | 2 | 11.266 | 0.043 | 3.722 |
| Down | Vigun03g101100.5.p | GLUCAN ENDO-1,3-BETA-GLUCOSIDASE 4 | *Vigna unguiculata* | 3 | | 3 | 22.705 | 0.029 | 2.356 |
| Down | Vigun03g448800.1.p | ADP glucose pyrophosphorylase large subunit 1 | *Vigna unguiculata* | 6 | | 5 | 27.003 | 0.017 | 4.826 |
| Down | Vigun09g261900.1.p | glyceraldehyde-3-phosphate dehydrogenase B subunit | *Vigna unguiculata* | 22 | | 13 | 150.402 | 0.015 | 8.598 |
| Down | Vigun11g162300.5.p | hypothetical protein (K09955) | *Vigna unguiculata* | 3 | | 2 | 17.386 | 0.012 | 2.873 |
| Down | Vigun06g110700.1.p | DNA-DAMAGE-REPAIR/TOLERATION PROTEIN DRT102 | *Vigna unguiculata* | 14 | | 13 | 115.569 | 0.011 | 3.247 |
| Down | Vigun01g053400.1.p | phosphoglycerate kinase | *Vigna unguiculata* | 11 | | 8 | 69.315 | 0.023 | 5.065 |
|  | **Cell cycle, differentiation and development** | |  |  | |  |  |  |  |
| Up | Vigun03g341500.3.p | heterogeneous nuclear ribonucleoprotein A1/A3 (HNRNPA1_3) | *Arabidopsis thaliana* | 6 | | 5 | 100.456 | 0.016 | 2.670 |
| Down | Vigun10g115500.1.p | Phosphopantothenoylcysteine decarboxylase / N-((R)-4'-phosphopantothenoyl)-L-cysteine carboxy-lyase | *Arabidopsis thaliana* | 4 | | 4 | 21.594 | 0.008 | 15.113 |
| Down | Vigun09g058800.1.p | chromosome transmission fidelity protein 4 (WDHD1, CTF4) | *Arabidopsis thaliana* | 77 | | 70 | 507.622 | 0.045 | 3.141 |
| Down | Vigun06g235100.1.p | DnaJ homolog subfamily C member 2 (DNAJC2) | *Arabidopsis thaliana* | 5 | | 4 | 17.123 | 0.015 | 6.122 |
| Down | Vigun01g253300.1.p | RNA recognition motif. (a.k.a. RRM, RBD, or RNP domain) (RRM_1) | *Arabidopsis thaliana* | 6 | | 6 | 50.677 | 0.018 | 5.032 |
|  | **Cellular component organization** | |  |  | |  |  |  |  |
| Up | Vigun03g290300.4.p | NUCLEAR MATRIX CONSTITUENT PROTEIN 1-LIKE PROTEIN-RELATED | *Arabidopsis thaliana* | 10 | | 8 | 38.006 | 0.011 | 9.189 |
| Up | Vigun09g209700.1.p | TARGETING PROTEIN FOR XKLP2-LIKE PROTEIN | *Arabidopsis thaliana* | 7 | | 7 | 37.839 | 0.030 | 3.454 |
| Up | Vigun08g055200.5.p | TPX2 (targeting protein for Xklp2) protein family | *Arabidopsis thaliana* | 6 | | 6 | 32.247 | 0.0218 | 5.253 |
| Down | Vigun08g194200.1.p | STRUCTURAL MAINTENANCE OF CHROMOSOMES SMC FAMILY MEMBER | *Arabidopsis thaliana* | 11 | | 10 | 50.854 | 0.029 | 2.156 |
| Down | Vigun08g219200.4.p | FASCICLIN-LIKE ARABINOGALACTAN PROTEIN 7 | *Arabidopsis thaliana* | 2 | | 2 | 22.101 | 0.036 | 5.999 |
| Down | Vigun11g175100.1.p | adenylyl cyclase-associated protein (CAP1_2, SRV2) | *Vigna unguiculata* | 8 | | 7 | 34.208 | 0.031 | 4.373 |
| Down | Vigun07g103500.1.p | HISTONE H1/H5 | *Vigna unguiculata* | 7 | | 7 | 33.436 | 0.03 | 3.724 |
| Down | Vigun09g033900.1.p | nucleosome assembly protein 1;2 | *Vigna unguiculata* | 8 | | 6 | 59.003 | 0.034 | 5.637 |
| Down | Vigun03g393100.1.p | nucleosome assembly protein 1;2 | *Vigna unguiculata* | 6 | | 4 | 34.192 | 0.034 | 7.525 |
|  | ***Membrane trafficking and intracellular transport*** | | |  | |  |  |  |  |
| Up | Vigun01g144200.1.p | charged multivesicular body protein 2B (CHMP2B) | *Vigna unguiculata* | 4 | | 4 | 25.788 | 0.019 | 6.657 |
| Up | Vigun03g015800.1.p | delta-adaptin | *Vigna unguiculata* | 2 | | 2 | 3.4214 | 0.024 | 5.238 |
| Up | Vigun09g254900.1.p | ZINC FINGER FYVE DOMAIN CONTAINING PROTEIN | *Arabidopsis thaliana* | 7 | | 7 | 35.884 | 0.029 | 2.290 |
| Down | Vigun07g094700.1.p | non-intrinsic ABC protein 4 | *Vigna unguiculata* | 4 | | 4 | 23.651 | 0.003 | 3.936 |
| Down | Vigun10g181000.1.p | AP-4 COMPLEX ACCESSORY SUBUNIT TEPSIN | *Vigna unguiculata* | 2 | | 2 | 14.34 | 0.010 | 4.343 |
| Down | Vigun11g215700.1.p | protein transport protein SEC31 (SEC31) | *Vigna unguiculata* | 3 | | 3 | 16.555 | 0.024 | 3.260 |
| Down | Vigun08g132300.1.p | translocon at the inner envelope membrane of chloroplasts 110 | *Arabidopsis thaliana* | 5 | | 3 | 19.418 | 0.037 | 3.418 |
| Down | Vigun03g031100.5.p | non-intrinsic ABC protein 7 | *Vigna unguiculata* | 5 | | 5 | 26.227 | 0.006 | 8.725 |
| Down | Vigun03g212600.1.p | voltage dependent anion channel 1 | *Vigna unguiculata* | 2 | | 2 | 14.66 | 0.02 | 9.669 |
| Down | Vigun04g092100.1.p | translocase of outer membrane 20 kDa subunit 3 | *Vigna unguiculata* | 6 | | 5 | 30.439 | 0.0005 | 4.557 |
| Down | Vigun07g038900.1.p | copper chaperone | *Vigna unguiculata* | 4 | | 4 | 37.416 | 0.010 | 8.003 |
| Down | Vigun03g202700.4.p | NUCLEAR TRANSPORT FACTOR 2 AND RNA RECOGNITION MOTIF DOMAIN-CONTAINING PROTEIN | *Vigna unguiculata* | 4 | | 3 | 20.629 | 0.016 | 2.903 |
| Down | Vigun06g045400.1.p | Ankyrin repeat family protein | *Arabidopsis thaliana* | 6 | | 6 | 55.104 | 0.02 | 3.368 |
|  | **Photosynthesis** |  |  |  | |  |  |  |  |
| Down | Vigun07g010900.1.p | proton gradient regulation 5 | *Vigna unguiculata* | 2 | | 2 | 7.669 | 0.038 | 5.460 |
| Down | Vigun01g208300.1.p | Photosystem II reaction center PsbP family protein | *Vigna unguiculata* | 7 | | 5 | 28.591 | 0.038 | 3.758 |
| Down | VigunL062800.1.p | photosystem II reaction center protein E | *Vigna unguiculata* | 4 | | 4 | 11.532 | 0.014 | 3.959 |
| Down | VigunL063200.1.p | photosynthetic electron transfer A | *Vigna unguiculata* | 6 | | 6 | 44.392 | 0.014 | 4.479 |
| Down | VigunL063700.1.p | photosystem II reaction center protein H | *Vigna unguiculata* | 3 | | 3 | 46.652 | 0.015 | 3.697 |
| Down | Vigun02g183600.1.p | ALBINA 1 | *Vigna unguiculata* | 3 | | 3 | 15.059 | 0.006 | 35.696 |
| Down | Vigun09g064700.1.p | Heme oxygenase (biliverdin-producing) / Heme oxygenase (decyclizing) | *Vigna unguiculata* | 9 | | 9 | 49.115 | 0.014 | 2.661 |
| Down | Vigun08g177800.1.p | Geranylgeranyl diphosphate reductase / Geranylgeranyl reductase | *Vigna unguiculata* | 30 | | 28 | 248.108 | 0.010 | 14.406 |
|  | **Protein degradation** | |  |  | |  |  |  |  |
| Down | Vigun09g241100.1.p | serpin B (SERPINB) | *Arabidopsis thaliana* | 25 | | 22 | 240.644 | 0.042 | 4.259 |
| Down | Vigun09g261100.1.p | EH DOMAIN // CALCIUM-BINDING EF HAND-CONTAINING PROTEIN | *Vigna unguiculata* | 21 | | 17 | 117.71 | 0.031 | 2.351 |
| Down | Vigun01g027800.1.p | ASPARTYL PROTEASES // ASPARTYL PROTEASE FAMILY PROTEIN-RELATED | *Vigna unguiculata* | 4 | | 4 | 13.999 | 0.043 | 3.603 |
| Down | Vigun09g021700.1.p | FTSH protease 8 | *Vigna unguiculata* | 20 | | 18 | 107.046 | 0.002 | 5.086 |
| Down | Vigun10g151400.1.p | PROPROTEIN CONVERTASE SUBTILISIN/KEXIN | *Vigna unguiculata* | 2 | | 2 | 9.411 | 0.045 | 17.416 |
| Down | Vigun10g198500.1.p | regulatory particle triple-A ATPase 6A | *Vigna unguiculata* | 5 | | 5 | 15.169 | 0.034 | 2.478 |
| Down | Vigun07g275900.1.p | 20S proteasome alpha subunit E2 | *Vigna unguiculata* | 4 | | 4 | 25.958 | 0.037 | 2.221 |
| Down | Vigun03g255000.1.p | ASPARTYL PROTEASES // ASPARTYL PROTEASE FAMILY PROTEIN | *Vigna unguiculata* | 8 | | 6 | 46.316 | 0.031 | 3.11 |
| Down | Vigun03g398000.1.p | ATP-DEPENDENT CLP PROTEASE PROTEOLYTIC SUBUNIT-RELATED PROTEIN 4, CHLOROPLASTIC | *Vigna unguiculata* | 4 | | 2 | 12.997 | 0.003 | 10.587 |
| Down | Vigun09g108700.3.p | serine carboxypeptidase-like 45 | *Vigna unguiculata* | 6 | | 6 | 65.669 | 0.031 | 3.578 |
| Down | Vigun09g232600.1.p | PROPROTEIN CONVERTASE SUBTILISIN/KEXIN | *Vigna unguiculata* | 3 | | 3 | 24.037 | 0.036 | 9.244 |
| Down | Vigun11g188300.1.p | PROPROTEIN CONVERTASE SUBTILISIN/KEXIN | *Vigna unguiculata* | 29 | | 27 | 405.380 | 0.010 | 6.039 |
| Down | Vigun10g065100.1.p | cystatin B | *Glycine max* | 7 | | 3 | 48.432 | 0.011 | 4.273 |
| Down | Vigun05g055100.3.p | SERINE PROTEASE FAMILY S10 SERINE CARBOXYPEPTIDASE | *Vigna unguiculata* | 2 | | 2 | 5.906 | 0.046 | 6.340 |
| Down | Vigun02g071000.1.p | Matrilysin / Uterine metalloendopeptidase | *Vigna unguiculata* | 3 | | 3 | 24.779 | 0.012 | 3.924 |
| Down | Vigun11g012900.1.p | peptidyl-prolyl isomerase H (cyclophilin H) (PPIH, CYPH) | *Vigna unguiculata* | 9 | | 6 | 73.889 | 0.012 | 3.849 |
| Down | Vigun01g203700.1.p | rotamase CYP 4 | *Vigna unguiculata* | 6 | | 6 | 46.804 | 0.0115 | 2.780 |
| Down | Vigun09g111600.1.p | Clp ATPase | *Vigna unguiculata* | 24 | | 21 | 150.766 | 0.003 | 3.879 |
|  | **Protein processing** |  |  |  | |  |  |  |  |
| Up | Vigun01g054100.1.p | F3O9.7 PROTEIN-RELATED | *Vigna unguiculata* | 9 | | 8 | 28.692 | 0.030 | 2.009 |
| Up | Vigun11g101800.1.p | unconventional prefoldin RPB5 interactor 1 (URI1) | *Vigna unguiculata* | 4 | | 4 | 36.914 | 0.031 | 4.338 |
| Up | Vigun03g260100.1.p | peptidylprolyl cis/trans isomerase, NIMA-interacting 1 | *Arabidopsis thaliana* | 9 | | 9 | 58.184 | 0.030 | 3.291 |
| Down | Vigun09g273100.1.p | rotamase CYP 3 | *Vigna unguiculata* | 5 | | 1 | 26.581 | 0.020 | 3.061 |
| Down | Vigun09g274300.2.p | PEPTIDYL-PROLYL CIS-TRANS ISOMERASE | *Vigna unguiculata* | 12 | | 10 | 63.998 | 0.023 | 3.326 |
| Down | Vigun05g010200.3.p | PEPTIDYL-PROLYL CIS-TRANS ISOMERASE // PEPTIDYL-PROLYL CIS-TRANS ISOMERASE FKBP19, CHLOROPLASTIC | *Vigna unguiculata* | 3 | | 2 | 8.591 | 0.010 | 13.425 |
| Down | Vigun03g049900.1.p | rotamase cyclophilin 2 | *Vigna unguiculata* | 9 | | 6 | 29.64 | 0.03 | 2.724 |
| Down | Vigun06g110200.1.p | Rubredoxin (Rubredoxin) | *Arabidopsis thaliana* | 4 | | 4 | 15.747 | 0.027 | 5.802 |
| Down | Vigun07g279900.1.p | Zim17-type zinc finger protein | *Arabidopsis thaliana* | 2 | | 2 | 10.874 | 0.036 | 8.557 |
| Down | Vigun08g184100.1.p | Rhodanese-like domain (Rhodanese) // PPIC-type PPIASE domain (Rotamase_3) | *Arabidopsis thaliana* | 2 | | 2 | 4.158 | 0.04 | 33.417 |
| Down | Vigun07g206500.1.p | Tetratricopeptide repeat (TPR_12) | *Arabidopsis thaliana* | 3 | | 3 | 24.455 | 0.010 | 2.482 |
| Down | Vigun05g204300.1.p | Uncharacterized protein | *Arabidopsis thaliana* | 2 | | 2 | 9.081 | 0.024 | 11.039 |
| Down | Vigun06g220200.1.p | PEPTIDYL-PROLYL CIS-TRANS ISOMERASE // PEPTIDYL-PROLYL CIS-TRANS ISOMERASE FKBP12 | *Vigna unguiculata* | 3 | | 3 | 25.592 | 0.013 | 2.663 |
| Down | Vigun02g199300.1.p | Salt stress response/antifungal (Stress-antifung) // Protein tyrosine kinase (Pkinase_Tyr) | *Vigna unguiculata* | 3 | | 3 | 5.316 | 0.032 | 4.683 |
| Down | Vigun10g007600.4.p | FKBP-like peptidyl-prolyl cis-trans isomerase family protein | *Vigna unguiculata* | 2 | | 2 | 4.742 | 0.010 | 5.28 |
| Down | Vigun04g121600.1.p | cytosolic prostaglandin-E synthase (PTGES3) | *Glycine max* | 3 | | 3 | 12.508 | 0.026 | 2.793 |
| Down | Vigun11g106300.1.p | protein tyrosine phosphatases;protein tyrosine phosphatases | *Vigna unguiculata* | 10 | | 10 | 98.819 | 0.028 | 2.00 |
| Down | Vigun11g064000.3.p | PEPTIDYL-PROLYL CIS-TRANS ISOMERASE CYP37, CHLOROPLASTIC | *Vigna unguiculata* | 9 | | 9 | 58.021 | 0.02 | 3.482 |
|  | **Protein synthesis** |  |  |  | |  |  |  |  |
| Up | Vigun11g189700.1.p | glycine-rich protein | *Arabidopsis thaliana* | 20 | | 15 | 82.144 | 0.028 | 4.664 |
| Up | Vigun09g140400.1.p | 30S RIBOSOMAL PROTEIN-RELATED | *Arabidopsis thaliana* | 10 | | 10 | 133.878 | 0.016 | 2.911 |
| Up | Vigun03g118500.1.p | eukaryotic translation initiation factor-related | *Vigna unguiculata* | 21 | | 20 | 124.915 | 0.031 | 9.799 |
| Up | Vigun10g016200.2.p | eukaryotic translation initiation factor 4G | *Arabidopsis thaliana* | 43 | | 39 | 364.551 | 0.030 | 3.519 |
| Down | Vigun04g032600.1.p | peptide deformylase 1B | *Arabidopsis thaliana* | 4 | | 4 | 20.008 | 0.0004 | 5.007 |
| Down | Vigun06g168800.1.p | TRANSLATION FACTOR | *Vigna unguiculata* | 18 | | 5 | 128.963 | 0.010 | 5.042 |
| Down | Vigun11g223300.1.p | translation initiation factor 3 subunit I (EIF3I) | *Arabidopsis thaliana* | 4 | | 3 | 10.206 | 0.022 | 3.154 |
| Down | Vigun05g299300.2.p | small subunit ribosomal protein S12e (RP-S12e, RPS12) | *Vigna unguiculata* | 4 | | 4 | 24.178 | 0.038 | 3.491 |
| Down | Vigun03g306300.1.p | Translation elongation factor EFG/EF2 protein | *Vigna unguiculata* | 5 | | 4 | 8.924 | 0.041 | 2.700 |
| Down | Vigun04g010500.3.p | elongation factor Ts (tsf, TSFM) | *Vigna unguiculata* | 68 | | 60 | 720.539 | 0.010 | 5.819 |
| Down | Vigun06g023900.1.p | ELONGATION FACTOR 1-BETA 1-RELATED | *Vigna unguiculata* | 13 | | 7 | 111.79 | 0.008 | 2.701 |
| Down | Vigun09g102300.2.p | Translation elongation factor EF1B/ribosomal protein S6 family protein | *Vigna unguiculata* | 10 | | 4 | 83.921 | 0.045 | 6.605 |
| Down | Vigun10g029400.1.p | Ribosomal protein S6e | *Vigna unguiculata* | 3 | | 3 | 11.233 | 0.006 | 7.449 |
| Down | Vigun11g152000.2.p | ribosomal protein L23AB | *Vigna unguiculata* | 5 | | 4 | 13.258 | 0.010 | 4.811 |
| Down | Vigun11g196500.1.p | ribosomal protein L12-A | *Vigna unguiculata* | 13 | | 12 | 118.302 | 0.011 | 2.635 |
| Down | Vigun07g079000.1.p | large subunit ribosomal protein L4e (RP-L4e, RPL4) | *Vigna unguiculata* | 3 | | 3 | 12.885 | 0.01 | 4.184 |
| Down | Vigun05g086500.1.p | elongation factor P (efp) | *Vigna unguiculata* | 7 | | 5 | 50.291 | 0.029 | 4.497 |
| Down | Vigun08g161600.1.p | small subunit ribosomal protein S26e (RP-S26e, RPS26) | *Vigna unguiculata* | 3 | | 3 | 7.959 | 0.042 | 3.57 |
| Down | Vigun11g214900.1.p | large subunit ribosomal protein L1 (RP-L1, MRPL1, rplA) | *Vigna unguiculata* | 7 | | 7 | 27.75 | 0.029 | 3.087 |
| Down | Vigun03g422300.2.p | Isoleucine--tRNA ligase / Isoleucyl-tRNA synthetase | *Vigna unguiculata* | 8 | | 5 | 43.659 | 0.024 | 8.44 |
|  | **Signal transduction** |  |  |  | |  |  |  |  |
| Up | Vigun04g009800.1.p | AFP HOMOLOG 2 | *Vigna unguiculata* | 8 | | 7 | 37.178 | 0.023 | 2.685 |
| Up | Vigun07g233400.1.p | disease resistance protein (RAR1) | *Vigna unguiculata* | 4 | | 4 | 18.243 | 0.021 | 7.865 |
| Up | Vigun05g048200.1.p | plasminogen activator inhibitor 1 RNA-binding protein (SERBP1) | *Arabidopsis thaliana* | 26 | | 25 | 214.774 | 0.017 | 4.997 |
| Up | Vigun10g009600.1.p | plasminogen activator inhibitor 1 RNA-binding protein (SERBP1) | *Arabidopsis thaliana* | 22 | | 17 | 245.551 | 0.008 | 7.558 |
| Down | Vigun02g145800.3.p | INOSINE-5-MONOPHOSPHATE DEHYDROGENASE RELATED | *Vigna unguiculata* | 4 | | 4 | 5.349 | 0.016 | 3.399 |
|  | **Stress-related proteins: defense response** | |  |  | |  |  |  |  |
| Up | Vigun01g206400.3.p | G-patch domain (G-patch) // Zinc-finger double-stranded RNA-binding (zf-C2H2_jaz) | *Arabidopsis thaliana* | 2 | | 2 | 10.528 | 0.025 | 4.581 |
| Down | Vigun03g225500.1.p | ABSCISIC ACID RECEPTOR PYL1-RELATED | *Arabidopsis thaliana* | 7 | | 5 | 49.959 | 0.011 | 4.223 |
| Down | Vigun05g143700.1.p | kunitz trypsin inhibitor 1 | *Arabidopsis thaliana* | 2 | | 2 | 18.701 | 0.050 | 5.245 |
| Down | Vigun09g010000.1.p | Pathogenesis-related protein Bet v I family (Bet_v_1) | *Vigna unguiculata* | 10 | | 10 | 77.582 | 0.038 | 3.699 |
| Down | Vigun08g179200.3.p | RNA-binding (RRM/RBD/RNP motifs) family protein | *Arabidopsis thaliana* | 7 | | 6 | 25.443 | 0.041 | 2.725 |
| Down | Vigun09g278000.1.p | basic chitinase | *Vigna unguiculata* | 2 | | 2 | 12.063 | 0.036 | 4.601 |
|  | **Stress-related proteins: heat stress response** | |  |  | |  |  |  |  |
| Up | Vigun08g042300.1.p | 17.6 kDa class II heat shock protein | *Arabidopsis thaliana* | 9 | | 5 | 100.384 | 0.029 | 2.309 |
| Up | Vigun09g222500.1.p | 17.6 kDa class II heat shock protein | *Arabidopsis thaliana* | 18 | | 17 | 210.328 | 0.028 | 2.800 |
| Up | Vigun08g155100.1.p | 17.6 KDA CLASS I HEAT SHOCK PROTEIN 1-RELATED | *Arabidopsis thaliana* | 34 | | 22 | 356.769 | 0.004 | 8.246 |
| Up | Vigun03g104500.1.p | SMALL HEAT-SHOCK PROTEIN HSP20 FAMILY // 17.6 KDA CLASS I HEAT SHOCK PROTEIN 1-RELATED | *Arabidopsis thaliana* | 34 | | 13 | 400.326 | 0.010 | 7.003 |
| Up | Vigun07g167700.1.p | SMALL HEAT-SHOCK PROTEIN HSP20 FAMILY // 22.0 KDA HEAT SHOCK PROTEIN | *Arabidopsis thaliana* | 19 | | 18 | 174.301 | 0.003 | 4.912 |
| Up | Vigun08g155000.1.p | 17.6 KDA CLASS I HEAT SHOCK PROTEIN 1-RELATED | *Arabidopsis thaliana* | 17 | | 13 | 175.577 | 0.014 | 4.225 |
| Up | Vigun03g104700.1.p | HSP20-like chaperones superfamily protein | *Arabidopsis thaliana* | 32 | | 1 | 364.678 | 0.037 | 15.195 |
| Up | Vigun10g139700.1.p | BAG FAMILY MOLECULAR CHAPERONE REGULATOR 6 | *Arabidopsis thaliana* | 16 | | 14 | 60.149 | 0.037 | 4.888 |
| Up | Vigun09g120200.1.p | 17.6 KDA CLASS I HEAT SHOCK PROTEIN 1-RELATED | *Arabidopsis thaliana* | 19 | | 6 | 148.070 | 0.012 | 8.623 |
| Up | Vigun03g104800.1.p | 17.6 KDA CLASS I HEAT SHOCK PROTEIN 1-RELATED | *Arabidopsis thaliana* | 30 | | 9 | 256.267 | 0.011 | 8.884 |
| Up | Vigun04g168500.1.p | 22.0 KDA HEAT SHOCK PROTEIN | *Arabidopsis thaliana* | 15 | | 15 | 112.155 | 0.010 | 2.208 |
| Up | Vigun04g017000.1.p | multiprotein bridging factor 1C | *Arabidopsis thaliana* | 6 | | 6 | 26.261 | 0.032 | 8.170 |
| Up | Vigun09g150000.1.p | NUCLEAR MIGRATION PROTEIN NUDC | *Arabidopsis thaliana* | 14 | | 10 | 116.999 | 0.043 | 2.809 |
| Down | Vigun07g125400.1.p | CALVIN CYCLE PROTEIN CP12-1, CHLOROPLASTIC-RELATED | *Arabidopsis thaliana* | 3 | | 3 | 24.697 | 0.019 | 5.654 |
| Down | Vigun10g191100.1.p | post-illumination chlorophyll fluorescence increase | *Arabidopsis thaliana* | 6 | | 4 | 27.223 | 0.006 | 3.49 |
| Down | Vigun01g150500.1.p | heat shock cognate protein 70-1 | *Arabidopsis thaliana* | 27 | | 2 | 177.841 | 0.021 | 5.424 |
|  | ***Stress-related proteins: oxidative stress response*** | | |  | |  |  |  |  |
| Up | Vigun01g168600.1.p | PROGRAMMED CELL DEATH 4 | *Arabidopsis thaliana* | 5 | | 5 | 50.846 | 0.004 | 7.002 |
| Up | Vigun10g188100.1.p | glutaredoxin 4 | *Vigna unguiculata* | 3 | | 3 | 16.968 | 0.037 | 2.118 |
| Down | Vigun09g238200.3.p | THIOREDOXIN-LIKE 4, CHLOROPLASTIC | *Vigna unguiculata* | 6 | | 5 | 36.345 | 0.013 | 5.04 |
| Down | Vigun03g187800.1.p | THIOREDOXIN | *Vigna unguiculata* | 3 | | 3 | 10.328 | 0.005 | 5.692 |
| Down | Vigun08g002500.1.p | chloroplastic drought-induced stress protein of 32 kD | *Vigna unguiculata* | 12 | | 11 | 118.587 | 0.010 | 4.1 |
| Down | Vigun10g081500.1.p | CAX-interacting protein 2 | *Vigna unguiculata* | 4 | | 3 | 15.394 | 0.024 | 2.904 |
| Down | Vigun03g409800.1.p | mitochondrial lipoamide dehydrogenase 1 | *Vigna unguiculata* | 17 | | 16 | 139.690 | 0.045 | 3.505 |
| Down | Vigun05g248900.1.p | Thioredoxin (Thioredoxin) // Thioredoxin-like domain (Thioredoxin_6) // Thioredoxin-like (Thioredoxin_7) | *Vigna unguiculata* | 25 | | 22 | 196.533 | 0.019 | 2.519 |
| Down | Vigun09g007400.1.p | lipoamide dehydrogenase 1 | *Vigna unguiculata* | 27 | | 14 | 277.175 | 0.008 | 4.548 |
| Down | Vigun03g142200.1.p | Tetrahydroberberine oxidase / THB oxidase | *Arabidopsis thaliana* | 9 | | 7 | 36.934 | 0.017 | 3.867 |
| Down | Vigun03g143500.1.p | BERBERINE BRIDGE ENZYME-RELATED | *Arabidopsis thaliana* | 8 | | 7 | 32.608 | 0.016 | 5.656 |
| Down | Vigun01g131500.1.p | glutathione peroxidase 4 | *Vigna unguiculata* | 9 | | 8 | 50.593 | 0.01 | 3.331 |
| Down | Vigun06g141000.1.p | PEROXIDASE 22-RELATED | *Vigna unguiculata* | 5 | | 4 | 41.457 | 0.035 | 2.171 |
| Down | Vigun06g141200.1.p | PEROXIDASE 22-RELATED | *Vigna unguiculata* | 4 | | 2 | 22.416 | 0.048 | 4.168 |
| Down | Vigun08g132800.1.p | Peroxidase / Lactoperoxidase | *Vigna unguiculata* | 5 | | 5 | 25.816 | 0.030 | 3.584 |
| Down | Vigun07g185500.1.p | Fe superoxide dismutase 2 | *Vigna unguiculata* | 15 | | 14 | 152.737 | 0.031 | 3.016 |
|  | **Stress-related proteins: water deprivation stress response** | | |  | |  |  |  |  |
| Up | Vigun07g272500.1.p | heterogeneous nuclear ribonucleoprotein G (RBMX, HNRNPG) | *Arabidopsis thaliana* | 5 | | 5 | 23.344 | 0.040 | 4.849 |
| Down | Vigun07g144500.2.p | translationally controlled tumor protein | *Arabidopsis thaliana* | 8 | | 6 | 55.616 | 0.03 | 2.873 |
| Down | Vigun09g154700.1.p | THIOREDOXIN PEROXIDASE | *Arabidopsis thaliana* | 6 | | 6 | 33.562 | 0.022 | 2.309 |
|  | **Stress-related proteins: other stress responses** | | |  | |  |  |  |  |
| Up | Vigun01g058300.1.p | glutathione S-transferase TAU 19 | *Glycine max* | 12 | | 11 | 73.554 | 0.037 | 2.991 |
| Down | Vigun03g346000.1.p | calcium sensing receptor | *Arabidopsis thaliana* | 3 | | 3 | 16.399 | 0.041 | 7.242 |
| Down | Vigun03g013400.1.p | UBX domain-containing protein 6 (UBXN6, UBXD1) | *Vigna unguiculata* | 2 | | 2 | 10.881 | 0.010 | 22.919 |
| Down | Vigun09g037800.1.p | germin 3 | *Arabidopsis thaliana* | 5 | | 5 | 113.139 | 0.027 | 2.271 |
| Down | Vigun03g397300.1.p | germin 3 | *Arabidopsis thaliana* | 5 | | 5 | 83.976 | 0.031 | 2.458 |
| Down | Vigun01g122600.1.p | MATRIX-REMODELING-ASSOCIATED PROTEIN 8 | *Arabidopsis thaliana* | 3 | | 3 | 14.766 | 0.036 | 2.640 |
| Down | Vigun10g192200.1.p | Universal stress protein family (Usp) | *Vigna unguiculata* | 9 | | 6 | 72.431 | 0.034 | 2.253 |
|  | **Transcription** |  |  |  | |  |  |  |  |
| Up | Vigun06g060000.3.p | WRKY TRANSCRIPTION FACTOR 20-RELATED | *Vigna unguiculata* | 2 | | 2 | 9.365 | 0.045 | 3.567 |
| Up | Vigun04g134900.1.p | VIRE2-interacting protein 1 | *Vigna unguiculata* | 6 | | 5 | 36.406 | 0.019 | 2.852 |
| Up | Vigun11g139200.1.p | TEOSINTE BRANCHED, cycloidea and PCF (TCP) 14 | *Vigna unguiculata* | 3 | | 3 | 13.463 | 0.012 | 6.462 |
| Up | Vigun09g169400.1.p | cold shock domain protein 1 | *Vigna unguiculata* | 6 | | 5 | 67.630 | 0.019 | 2.106 |
| Up | Vigun02g108600.1.p | SWIB/MDM2 domain (SWIB) | *Arabidopsis thaliana* | 4 | | 4 | 26.830 | 0.016 | 4.135 |
| Up | >Reverse Vigun05g276200.4.p | Uncharacterized protein | *Vigna unguiculata* | 30 | | 10 | 296.130 | 0.035 | 5.556 |
| Up | Vigun05g276200.4.p | Uncharacterized protein | *Vigna unguiculata* | 10 | | 10 | 46.460 | 0.034 | 3.139 |
| Up | Vigun02g186400.1.p | far upstream element-binding protein (FUBP) | *Arabidopsis thaliana* | 20 | | 2 | 198.550 | 0.024 | 5.494 |
| Up | Vigun11g166900.2.p | TRANSCRIPTION FACTOR POSF21-RELATED | *Vigna unguiculata* | 7 | | 5 | 49.814 | 0.031 | 3.351 |
| Up | Vigun07g100900.1.p | ATP BINDING / ATPASE-RELATED | *Vigna unguiculata* | 22 | | 19 | 218.593 | 0.028 | 3.120 |
| Up | Vigun07g025900.2.p | ZINC FINGER CCCH DOMAIN-CONTAINING PROTEIN 36-RELATED | *Arabidopsis thaliana* | 7 | | 6 | 29.364 | 0.036 | 2.197 |
| Up | Vigun03g155600.1.p | CCCH-type zinc finger family protein | *Arabidopsis thaliana* | 4 | | 4 | 12.973 | 0.036 | 4.117 |
| Up | Vigun11g049200.3.p | SART-1 family | *Vigna unguiculata* | 10 | | 10 | 55.342 | 0.029 | 5.320 |
| Up | Vigun03g084500.1.p | partner of Y14 and mago (WIBG, PYM) | *Arabidopsis thaliana* | 3 | | 2 | 24.918 | 0.032 | 4.566 |
| Up | Vigun05g259600.1.p | M-phase phosphoprotein 6, animal type (MPHOSPH6, MPP6) | *Arabidopsis thaliana* | 7 | | 5 | 22.210 | 0.038 | 5.944 |
| Up | Vigun03g331500.1.p | proline-rich family protein | *Vigna unguiculata* | 5 | | 5 | 31.106 | 0.014 | 6.073 |
| Up | Vigun07g132800.1.p | Uncharacterized protein | *Arabidopsis thaliana* | 3 | | 3 | 21.931 | 0.027 | 8.994 |
| Up | Vigun05g287400.3.p | CASC3/Barentsz eIF4AIII binding | *Arabidopsis thaliana* | 7 | | 7 | 25.939 | 0.031 | 3.084 |
| Up | Vigun06g211300.3.p | DAG PROTEIN-RELATED | *Arabidopsis thaliana* | 2 | | 2 | 10.784 | 0.017 | 2.893 |
| Up | Vigun03g344300.1.p | STEROL REGULATORY ELEMENT-BINDING PROTEIN // TRANSCRIPTION FACTOR BIM1 | *Arabidopsis thaliana* | 3 | | 2 | 3.172 | 0.012 | 23.829 |
| Down | Vigun02g144600.1.p | DNA-directed DNA polymerases | *Vigna unguiculata* | 5 | | 4 | 5.625 | 0.014 | 10.328 |
| Down | Vigun03g019600.2.p | NSP (nuclear shuttle protein)-interacting GTPase | *Vigna unguiculata* | 2 | | 2 | 9.45 | 0.028 | 3.018 |
| Down | Vigun05g040300.3.p | RNA-binding KH domain-containing protein | *Arabidopsis thaliana* | 6 | | 6 | 37.510 | 0.003 | 10.471 |
| Down | Vigun06g211000.1.p | NAD(P)H dehydrogenase (quinone) (wrbA) | *Vigna unguiculata* | 8 | | 8 | 45.130 | 0.046 | 3.522 |
| Down | Vigun09g005800.1.p | RNA RECOGNITION MOTIF-CONTAINING PROTEIN | *Arabidopsis thaliana* | 3 | | 3 | 3.729 | 0.045 | 3.488 |
| Down | Vigun03g189700.1.p | poly(A) binding protein 8 | *Arabidopsis thaliana* | 25 | | 21 | 196.007 | 0.010 | 3.689 |
| Down | Vigun11g175800.1.p | RNA recognition motif. (a.k.a. RRM, RBD, or RNP domain) (RRM_1) | *Arabidopsis thaliana* | 12 | | 9 | 80.050 | 0.004 | 11.204 |
| Down | Vigun07g110100.1.p | chloroplast stem-loop binding protein of 41 kDa | *Arabidopsis thaliana* | 19 | | 19 | 152.137 | 0.04 | 3.596 |
| Down | Vigun06g147300.4.p | varicose-related | *Arabidopsis thaliana* | 4 | | 4 | 20.892 | 0.015 | 152.696 |
| Down | Vigun08g025900.1.p | polyadenylate-binding protein (PABPC) | *Arabidopsis thaliana* | 9 | | 5 | 51.58 | 0.003 | 11.684 |
| Down | Vigun03g447700.2.p | POLYADENYLATE-BINDING PROTEIN RBP45B-RELATED | *Arabidopsis thaliana* | 4 | | 4 | 12.162 | 0.0004 | 5.783 |
| Down | Vigun07g183100.1.p | polyadenylate-binding protein 2 (PABPN1, PABP2) | *Arabidopsis thaliana* | 5 | | 5 | 35.577 | 0.013 | 5.479 |
| Down | Vigun07g250100.1.p | pre-mRNA-processing protein 40B | *Arabidopsis thaliana* | 2 | | 2 | 4.33 | 0.028 | 7.299 |
| Down | Vigun02g184600.1.p | RNA-binding protein 8A (RBM8A, Y14) | *Vigna unguiculata* | 2 | | 2 | 7.91 | 0.031 | 6.626 |
| Down | Vigun07g260000.1.p | splicing factor 3A subunit 1 (SF3A1, SAP114) | *Vigna unguiculata* | 4 | | 4 | 6.436 | 0.003 | 14.724 |
| Down | Vigun09g127000.2.p | RNA-binding protein 39 (RBM39, RNPC2) | *Vigna unguiculata* | 3 | | 2 | 5.864 | 0.019 | 2.729 |
| Down | Vigun01g204800.1.p | DEAD box RNA helicase (RH3) | *Arabidopsis thaliana* | 5 | | 4 | 43.465 | 0.043 | 2.044 |
|  | **Other metabolisms** |  |  |  | |  |  |  |  |
| Up | Vigun11g207300.1.p | ALCOHOL DEHYDROGENASE RELATED | *Glycine max* | 3 | | 3 | 19.055 | 0.013 | 7.641 |
| Down | Vigun05g172400.1.p | glyoxalase II 3 | *Arabidopsis thaliana* | 2 | | 2 | 8.067 | 0.035 | 7.376 |
| Down | Vigun03g148800.1.p | urease accessory protein G | *Vigna unguiculata* | 5 | | 5 | 29.369 | 0.039 | 3.360 |
| Down | Vigun07g289700.1.p | NFU domain protein 4 | *Vigna unguiculata* | 4 | | 4 | 22.62 | 0.03 | 3.681 |
| Down | Vigun07g223300.1.p | N6-ADENOSINE-METHYLTRANSFERASE SUBUNIT METTL14 | *Vigna unguiculata* | 6 | | 6 | 34.773 | 0.04 | 3.186 |
| Down | Vigun03g036300.1.p | NUCLEOSIDE DIPHOSPHATE KINASE // NUCLEOSIDE DIPHOSPHATE KINASE-RELATED | *Vigna unguiculata* | 3 | | 2 | 28.62 | 0.024 | 4.902 |
| Down | Vigun10g052700.3.p | uracil phosphoribosyltransferase | *Vigna unguiculata* | 7 | | 6 | 31.62 | 0.043 | 3.666 |
| Down | Vigun02g001400.1.p | Uncharacterized protein | *Arabidopsis thaliana* | 7 | | 7 | 30.816 | 0.049 | 3.208 |
| Down | Vigun05g290600.1.p | CARBOXYLASE:PYRUVATE/ACETYL-COA/PROPIONYL-COA CARBOXYLASE | *Arabidopsis thaliana* | 6 | | 6 | 41.547 | 0.038 | 2.675 |
| Down | Vigun02g037300.1.p | allene oxide cyclase 3 | *Vigna unguiculata* | 6 | | 5 | 41.525 | 0.029 | 2.305 |
| Down | Vigun04g195700.1.p | sirohydrochlorin ferrochelatase B | *Arabidopsis thaliana* | 3 | | 3 | 24.615 | 0.036 | 3.136 |
| Down | Vigun05g205500.1.p | Plastocyanin-like domain (Cu_bind_like) | *Vigna unguiculata* | 2 | | 2 | 16.445 | 0.013 | 2.869 |
| Down | Vigun06g203900.1.p | Alpha-helical ferredoxin | *Arabidopsis thaliana* | 4 | | 3 | 6.196 | 0.028 | 3.110 |
| Down | Vigun06g023500.1.p | 4-(cytidine 5\'-phospho)-2-C-methyl-D-erithritol kinase | *Vigna unguiculata* | 3 | | 3 | 11.227 | 0.014 | 4.185 |
| Down | Vigun10g138900.1.p | RETICULON // RETICULON-LIKE PROTEIN B4 | *Vigna unguiculata* | 2 | | 2 | 17.873 | 0.010 | 8.139 |
| Down | Vigun01g056100.1.p | aldehyde dehydrogenase 5F1 | *Vigna unguiculata* | 4 | | 4 | 21.336 | 0.032 | 8.002 |
| Down | Vigun10g139200.1.p | chloroplast sulfur E | *Arabidopsis thaliana* | 12 | | 11 | 76.877 | 0.032 | 2.206 |
| Down | Vigun01g219300.1.p | rubisco activase | *Arabidopsis thaliana* | 33 | | 19 | 312.155 | 0.029 | 3.761 |
| Down | Vigun11g079100.1.p | early nodulin-like protein 9 | *Vigna unguiculata* | 3 | | 3 | 11.451 | 0.024 | 2.875 |
| Down | Vigun04g188000.1.p | 2-methylene-furan-3-one reductase / Enone oxidoreductase | *Vigna unguiculata* | 23 | | 21 | 224.758 | 0.031 | 2.059 |
| Down | Vigun09g124100.1.p | Zinc finger C-x8-C-x5-C-x3-H type (and similar) (zf-CCCH) | *Arabidopsis thaliana* | 3 | | 2 | 9.361 | 0.04 | 3.983 |
|  | **Function unknown** |  |  |  | |  |  |  |  |
| Up | Vigun07g078800.1.p | ZINC FINGER-CONTAINING PROTEIN P48ZNF | *Vigna unguiculata* | 11 | | 9 | 50.658 | 0.031 | 4.217 |
| Up | Vigun07g251900.1.p | cyclin-related | *Vigna unguiculata* | 2 | | 2 | 15.971 | 0.029 | 24.328 |
| Down | Vigun03g053300.1.p | MITOCHONDRIAL OUTER MEMBRANE PROTEIN 25 | *Vigna unguiculata* | 7 | | 7 | 44.591 | 0.024 | 2.902 |
| Down | Vigun09g220600.1.p | ferredoxin-related | *Vigna unguiculata* | 2 | | 2 | 20.837 | 0.013 | 12.301 |
| Up | >Reverse Vigun02g125000.1.p | PHD FINGER, SWIB/MDM2 AND GYF DOMAIN-CONTAINING PROTEIN | *Vigna unguiculata* | 61 | | 54 | 593.068 | 0.012 | 2.775 |
| Up | Vigun02g071300.1.p | SAM domain (Sterile alpha motif) (SAM_1) | *Vigna unguiculata* | 2 | | 2 | 6.5311 | 0.028 | 41.711 |
| Down | Vigun08g011200.1.p | GTP-BINDING PROTEIN 10 | *Vigna unguiculata* | 2 | | 2 | 11.119 | 0.031 | 7.826 |
| Down | Vigun07g283100.1.p | RETICULOCALBIN // IP16409P | *Vigna unguiculata* | 3 | | 3 | 18.530 | 0.004 | 32.768 |
| Up | Vigun05g061300.3.p | Uncharacterized protein | *Vigna unguiculata* | 7 | | 6 | 36.603 | 0.042 | 3.808 |
| Down | Vigun11g179500.1.p | MALIC ENZYME-RELATED | *Vigna unguiculata* | 6 | | 6 | 21.428 | 0.011 | 5.809 |
| Up | Vigun01g061300.2.p | ENDOSULFINE | *Vigna unguiculata* | 4 | | 4 | 17.025 | 0.028 | 2.978 |
| Up | Vigun10g111800.3.p | MYELOID LEUKEMIA FACTOR | *Vigna unguiculata* | 4 | | 4 | 28.714 | 0.003 | 18.920 |
| Up | Vigun01g195500.1.p | Uncharacterized protein | *Vigna unguiculata* | 5 | | 5 | 37.444 | 0.004 | 3.848 |
| Down | Vigun09g024600.1.p | HISTIDINE TRIAD HIT PROTEIN // HISTIDINE TRIAD NUCLEOTIDE-BINDING PROTEIN 1 | *Vigna unguiculata* | 4 | | 4 | 39.203 | 0.045 | 2.027 |
| Up | Vigun04g196600.1.p | Protein of unknown function (DUF1421) | *Vigna unguiculata* | 19 | | 18 | 183.098 | 0.048 | 3.474 |
| Up | Vigun08g167100.1.p | Uncharacterized protein | *Vigna unguiculata* | 3 | | 2 | 14.239 | 0.024 | 4.338 |
| Up | Vigun03g122600.1.p | Octicosapeptide/Phox/Bem1p family protein | *Vigna unguiculata* | 9 | | 7 | 59.129 | 0.016 | 6.695 |
| Up | Vigun05g068200.1.p | AT hook motif DNA-binding family protein | *Vigna unguiculata* | 5 | | 3 | 31.589 | 0.015 | 2.173 |
| Up | Vigun08g035400.1.p | Uncharacterized protein | *Vigna unguiculata* | 10 | | 10 | 69.433 | 0.037 | 2.305 |
| Down | Vigun10g141500.1.p | Uncharacterized protein | *Vigna unguiculata* | 2 | | 2 | 7.198 | 0.031 | 7.407 |
| Down | Vigun03g270400.1.p | CYSTEINE-RICH SECRETORY PROTEIN-RELATED // CAP (CYSTEINE-RICH SECRETORY PROTEINS, ANTIGEN 5, AND PATHOGENESIS-RELATED 1 PROTEIN) SUPERFAMILY PROTEIN | *Vigna unguiculata* | 2 | | 2 | 6.876 | 0.011 | 8.955 |
| Down | Vigun02g153000.1.p | BSD domain-containing protein | *Vigna unguiculata* | 2 | | 2 | 4.255 | 0.024 | 10.530 |
| Down | Vigun01g076000.3.p | translocon-associated protein beta (TRAPB) family protein | *Vigna unguiculata* | 2 | | 2 | 12.919 | 0.010 | 12.465 |
| Down | Vigun07g209700.1.p | Angio-associated migratory cell protein (contains WD40 repeats) | *Vigna unguiculata* | 2 | | 2 | 8.372 | 0.037 | 6.124 |
| Up | Vigun03g322400.1.p | modifier of snc1 | *Vigna unguiculata* | 52 | | 50 | 391.399 | 0.036 | 2.208 |
| Down | Vigun09g110600.1.p | MITOCHONDRIAL OUTER MEMBRANE PROTEIN 25 | *Vigna unguiculata* | 4 | | 4 | 25.986 | 0.010 | 5.012 |
| Down | Vigun10g088500.1.p | phenylpyruvate tautomerase (MIF) | *Vigna unguiculata* | 5 | | 5 | 89.224 | 0.029 | 2.598 |
| Down | Vigun11g197500.1.p | translocon-associated protein subunit alpha (SSR1) | *Vigna unguiculata* | 4 | | 4 | 29.406 | 0.011 | 2.776 |
| Down | Vigun01g202400.1.p | PROTEIN FAM192A | *Vigna unguiculata* | 5 | | 4 | 20.260 | 0.03 | 2.286 |
| Down | Vigun03g080200.1.p | large subunit ribosomal protein LP1 (RP-LP1, RPLP1) | *Vigna unguiculata* | 2 | | 2 | 19.27 | 0.011 | 15.401 |
| Down | Vigun09g220700.1.p | Uncharacterized protein | *Vigna unguiculata* | 3 | | 2 | 16.0006 | 0.031 | 5.469 |
| Up | Vigun03g091000.1.p | PEPTIDASE-C1 DOMAIN-CONTAINING PROTEIN | *Vigna unguiculata* | 9 | | 7 | 63.115 | 0.035 | 4.355 |
| Up | Vigun09g207300.1.p | ZINC FINGER PROTEIN | *Vigna unguiculata* | 2 | | 2 | 16.970 | 0.040 | 7.678 |
| Up | Vigun10g190100.1.p | 26.5 KDA HEAT SHOCK PROTEIN, MITOCHONDRIAL | *Vigna unguiculata* | 22 | | 20 | 234.265 | 0.019 | 7.288 |
| Up | Vigun07g143800.1.p | Dormancy/auxin associated family protein | *Vigna unguiculata* | 3 | | 3 | 22.751 | 0.012 | 38.151 |
| Down | Vigun07g287300.1.p | plastid transcriptionally active 17 | *Vigna unguiculata* | 6 | | 6 | 44.693 | 0.008 | 7.114 |
| Up | Vigun04g087600.1.p | SMALL HEAT-SHOCK PROTEIN HSP20 FAMILY | *Vigna unguiculata* | 23 | | 18 | 167.894 | 0.003 | 7.953 |
| Down | Vigun07g249000.1.p | splicing factor 3A subunit 1 (SF3A1, SAP114) | *Vigna unguiculata* | 3 | | 2 | 8.984 | 0.010 | 3.77 |
| Down | Vigun10g137900.1.p | LRR and NB-ARC domains-containing disease resistance protein | *Vigna unguiculata* | 41 | | 36 | 316.389 | 0.043 | 2.202 |
| Down | Vigun03g185300.1.p | alpha/beta-Hydrolases superfamily protein | *Vigna unguiculata* | 2 | | 2 | 5.346 | 0.010 | 51.159 |
| Up | Vigun06g052200.1.p | heat shock protein 21 | *Vigna unguiculata* | 31 | | 30 | 284.240 | 0.036 | 2.772 |
| Down | Vigun11g180100.1.p | RNA-binding protein (yhbY) | *Vigna unguiculata* | 3 | | 3 | 20.222 | 0.038 | 3.48 |
| Down | Vigun10g015200.2.p | RNA-binding (RRM/RBD/RNP motifs) family protein | *Vigna unguiculata* | 16 | | 15 | 101.701 | 0.008 | 4.371 |
| Up | Vigun08g063100.1.p | SELENOPROTEIN H | *Vigna unguiculata* | 9 | | 8 | 52.941 | 0.010 | 3.367 |
| Down | Vigun01g234400.1.p | apoptotic chromatin condensation inducer in the nucleus (ACIN1, ACINUS) | *Vigna unguiculata* | 14 | | 12 | 76.663 | 0.011 | 5.314 |
| Down | Vigun04g030400.1.p | EF-HAND CALCIUM-BINDING DOMAIN CONTAINING PROTEIN | *Vigna unguiculata* | 2 | | 2 | 10.189 | 0.029 | 5.751 |
