# Supplementary material for: Regulation and physiological function of proteins for heat tolerance in cowpea (Vigna unguiculata) genotypes under controlled and field conditions
Source: Front Plant Sci. 2022 Aug 22;13:954527. doi: 10.3389/fpls.2022.954527 (PMC9441852; doi:10.3389/fpls.2022.954527)
Supplement: Supplementary file 2 [file Table_2.DOCX]

Table 2: List of responsive proteins regulated at Marapyane compared to at Eensaamheid from IT-96D-610 using the label free quantification and database searches.

| **Regulation** | **Accession** | **Protein name** | **Species names of orthologues** | **Peptide count** | **Unique peptides** | **Confidence score** | **q Value** | **Max fold change** |
| --- | --- | --- | --- | --- | --- | --- | --- | --- |
|  | **Amine and amide metabolic process** | |  |  |  |  |  |  |
| Down | Vigun01g245000.1.p | 3-OXOACYL-[ACYL-CARRIER-PROTEIN] REDUCTASE, CHLOROPLASTIC | *Vigna unguiculata* | 5 | 3 | 18.465 | 0.002 | 2.372 |
| Down | Vigun02g076200.1.p | spermidine synthase 2 | *Vigna unguiculata* | 3 | 2 | 28.663 | 0.012 | 2.185 |
| Down | Vigun07g047500.1.p | PTERIN-4-ALPHA-CARBINOLAMINE DEHYDRATASE | *Vigna unguiculata* | 6 | 6 | 37.167 | 0.007 | 2.227 |
|  | **Amino acid metabolism** | |  |  |  |  |  |  |
| Up | Vigun02g194500.2.p | O-acetylserine (thiol) lyase (OAS-TL) isoform A1 | *Vigna unguiculata* | 8 | 3 | 34.570 | 0.025 | 3.432 |
| Up | Vigun05g298700.1.p | isopropylmalate dehydrogenase 2 | *Vigna unguiculata* | 13 | 12 | 95.011 | 0.039 | 2.477 |
| Up | Vigun07g229500.2.p | Dihydrodipicolinate reductase, bacterial/plant | *Vigna unguiculata* | 4 | 4 | 11.918 | 0.038 | 3.256 |
| Up | Vigun08g125700.1.p | D-3-phosphoglycerate dehydrogenase | *Vigna unguiculata* | 8 | 7 | 28.677 | 0.02 | 3.644 |
| Down | Vigun06g195000.1.p | D-tyrosyl-tRNA(Tyr) deacylase [EC:3.1.-.-] (dtd, DTD1) | *Vigna unguiculata* | 2 | 2 | 5.632 | 0.025 | 2.083 |
| Down | Vigun01g138500.4.p | Indole-3-glycerol-phosphate lyase / TSA | *Vigna unguiculata* | 5 | 5 | 44.364 | 0.003 | 2.184 |
| Down | Vigun01g201000.1.p | S-adenosylmethionine synthetase 2 | *Vigna unguiculata* | 15 | 4 | 82.874 | 0.003 | 8.852 |
| Down | Vigun06g047300.1.p | tryptophan synthase beta-subunit 2 | *Vigna unguiculata* | 5 | 4 | 20.814 | 0.013 | 2.242 |
| Down | Vigun06g097100.1.p | glutamate decarboxylase | *Vigna unguiculata* | 7 | 3 | 37.724 | 0.013 | 2.588 |
| Down | Vigun06g142700.1.p | Selenocysteine lyase / Selenocysteine reductase | *Vigna unguiculata* | 7 | 7 | 32.619 | 0.002 | 2.295 |
| Down | Vigun07g000300.1.p | methylenetetrahydrofolate reductase 2 | *Vigna unguiculata* | 6 | 6 | 44.975 | 0.008 | 2.347 |
| Down | Vigun07g055900.1.p | glycine cleavage system H protein (gcvH, GCSH) | *Vigna unguiculata* | 2 | 2 | 13.546 | 0.041 | 2.595 |
| Down | Vigun07g182100.1.p | isopropyl malate isomerase large subunit 1 | *Vigna unguiculata* | 6 | 5 | 26.301 | 0.004 | 3.961 |
| Down | Vigun08g102900.1.p | 3-deoxy-7-phosphoheptulonate synthase / Phospho-2-oxo-3-deoxyheptonate aldolase | *Vigna unguiculata* | 10 | 5 | 54.956 | 0.004 | 12.792 |
| Down | Vigun08g221200.2.p | PHE ammonia lyase 1 | *Vigna unguiculata* | 4 | 4 | 19.969 | 0.014 | 2.657 |
| Down | Vigun09g177800.1.p | 3-deoxy-7-phosphoheptulonate synthase / Phospho-2-oxo-3-deoxyheptonate aldolase | *Vigna unguiculata* | 12 | 7 | 51.532 | 0.002 | 2.001 |
| Down | Vigun11g078900.1.p | thiazole biosynthetic enzyme, chloroplast (ARA6) (THI1) (THI4) | *Arabidopsis thaliana* | 15 | 14 | 114.414 | 0.002 | 2.785 |
| Down | Vigun07g071800.1.p | methionine adenosyltransferase 3 | *Vigna unguiculata* | 16 | 7 | 106.754 | 0.002 | 4.137 |
| Down | Vigun04g056600.1.p | chorismate mutase 1 | *Vigna unguiculata* | 3 | 3 | 11.629 | 0.015 | 2.132 |
|  | **Carbohydrate and energy metabolism** | |  |  |  |  |  |  |
| Up | Vigun10g151700.1.p | NDP-glucose--starch glucosyltransferase / Waxy protein | *Vigna unguiculata* | 5 | 5 | 38.593 | 0.006 | 3.862 |
| Up | Vigun01g079000.2.p | ALPHA-MANNOSIDASE | *Vigna unguiculata* | 5 | 3 | 16.217 | 0.031 | 2.536 |
| Up | Vigun07g252100.1.p | PERIPLASMIC BETA-GLUCOSIDASE-RELATED | *Vigna unguiculata* | 6 | 6 | 26.077 | 0.006 | 14.459 |
| Up | Vigun08g213100.2.p | FRUCTOSE-BISPHOSPHATE ALDOLASE // FRUCTOSE-BISPHOSPHATE ALDOLASE-RELATED | *Vigna unguiculata* | 23 | 19 | 269.705 | 0.007 | 2.558 |
| Up | Vigun01g086500.1.p | NDH-dependent cyclic electron flow 1 | *Vigna unguiculata* | 9 | 9 | 63.301 | 0.006 | 2.177 |
| Up | Vigun01g100500.1.p | alpha-L-arabinofuranosidase 1 | *Vigna unguiculata* | 7 | 7 | 29.425 | 0.005 | 2.549 |
| Up | Vigun02g098200.1.p | phosphoribulokinase | *Vigna unguiculata* | 28 | 27 | 313.350 | 0.003 | 2.25 |
| Up | Vigun08g214900.1.p | ADP glucose pyrophosphorylase 1 | *Vigna unguiculata* | 23 | 21 | 116.322 | 0.018 | 2.198 |
| Up | Vigun09g109500.1.p | ALPHA-MANNOSIDASE | *Vigna unguiculata* | 7 | 5 | 31.525 | 0.005 | 2.436 |
| Up | Vigun09g260000.1.p | starch branching enzyme 2.2 | *Vigna unguiculata* | 7 | 6 | 45.436 | 0.007 | 8.887 |
| Up | Vigun11g046200.1.p | chitinase A | *Vigna unguiculata* | 6 | 6 | 34.864 | 0.024 | 3.324 |
| Up | Vigun11g176800.2.p | GLUCOSE-1-PHOSPHATE ADENYLYLTRANSFERASE LARGE SUBUNIT 3, CHLOROPLASTIC-RELATED | *Vigna unguiculata* | 25 | 24 | 154.529 | 0.001 | 2.633 |
| Up | VigunL009100.1.p | ALPHA-GALACTOSIDASE 1 | *Arabidopsis thaliana* | 4 | 2 | 14.203 | 0.002 | 7.684 |
| Down | Vigun07g255900.1.p | 1,2-alpha-L-fucosidases | *Vigna unguiculata* | 8 | 8 | 47.76 | 0.027 | 2.528 |
| Down | Vigun05g268500.1.p | pfkB-like carbohydrate kinase family protein | *Vigna unguiculata* | 9 | 4 | 53.596 | 0.002 | 3.164 |
| Down | Vigun02g000600.1.p | PYROPHOSPHATE--FRUCTOSE 6-PHOSPHATE 1-PHOSPHOTRANSFERASE SUBUNIT ALPHA 1-RELATED | *Vigna unguiculata* | 5 | 5 | 35.287 | 0.016 | 2.219 |
| Down | Vigun04g141300.1.p | plastidic pyruvate kinase beta subunit 1 | *Vigna unguiculata* | 10 | 8 | 36.332 | 0.002 | 5.373 |
| Down | Vigun07g169700.1.p | PLASTIDIAL PYRUVATE KINASE 1, CHLOROPLASTIC | *Vigna unguiculata* | 11 | 11 | 63.377 | 0.007 | 2.43 |
| Down | Vigun01g061600.1.p | glyceraldehyde-3-phosphate dehydrogenase of plastid 2 | *Vigna unguiculata* | 7 | 4 | 17.861 | 0.002 | 4.43 |
| Down | Vigun02g041200.1.p | alpha-xylosidase 1 | *Vigna unguiculata* | 16 | 14 | 72.367 | 0.029 | 2.253 |
| Down | Vigun02g124000.1.p | GDP-D-mannose 4,6-dehydratase 1 | *Vigna unguiculata* | 3 | 3 | 14.638 | 0.005 | 3.378 |
| Down | Vigun09g265100.1.p | GLUCAN ENDO-1,3-BETA-GLUCOSIDASE 7-RELATED | *Vigna unguiculata* | 2 | 2 | 3.261 | 0.016 | 4.25 |
| Down | Vigun11g037800.1.p | Glucan endo-1,3-beta-D-glucosidase / Laminarinase | *Vigna unguiculata* | 3 | 3 | 12.439 | 0.022 | 2.648 |
|  | **Cell cycle, differentiation and development** | |  |  |  |  |  |  |
| Up | Vigun05g282100.1.p | TETRAKETIDE ALPHA-PYRONE REDUCTASE 1 | *Arabidopsis thaliana* | 27 | 5 | 287.831 | 0.037 | 3.034 |
| Up | Vigun09g047500.3.p | Outer arm dynein light chain 1 protein | *Arabidopsis thaliana* | 4 | 4 | 3.849 | 0.002 | 7.936 |
| Down | Vigun03g305800.1.p | MONOCOPPER OXIDASE-LIKE PROTEIN SKU5 | *Arabidopsis thaliana* | 4 | 3 | 32.748 | 0.019 | 3.617 |
| Down | Vigun11g192700.2.p | Nuclear localization sequence binding protein | *Arabidopsis thaliana* | 12 | 11 | 65.375 | 0.003 | 3.837 |
| Down | Vigun09g017800.1.p | FASCICLIN-LIKE ARABINOGALACTAN PROTEIN 4 | *Arabidopsis thaliana* | 3 | 3 | 13.278 | 0.024 | 2.444 |
| Down | Vigun08g220400.1.p | FASCICLIN-LIKE ARABINOGALACTAN PROTEIN 1 | *Arabidopsis thaliana* | 3 | 3 | 15.704 | 0.019 | 3.002 |
| Down | Vigun04g157300.1.p | membrane-associated progesterone binding protein 3 | *Arabidopsis thaliana* | 3 | 2 | 19.277 | 0.011 | 2.601 |
| Down | Vigun02g191300.3.p | NADH-CYTOCHROME B5 REDUCTASE | *Arabidopsis thaliana* | 2 | 2 | 12.623 | 0.007 | 2.595 |
| Down | Vigun07g156400.1.p | Alba (Alba) | *Arabidopsis thaliana* | 6 | 5 | 34.415 | 0.003 | 2.221 |
| Down | Vigun07g076200.2.p | nucleolin (NCL, NSR1) | *Arabidopsis thaliana* | 7 | 3 | 92.588 | 0.008 | 2.812 |
| Down | Vigun09g096100.1.p | nucleolin (NCL, NSR1) | *Arabidopsis thaliana* | 14 | 13 | 169.607 | 0.002 | 2.305 |
| Down | Vigun01g253300.1.p | glycine-rich RNA-binding protein 2 | *Arabidopsis thaliana* | 6 | 4 | 49.502 | 0.029 | 2.081 |
| Down | Vigun06g071300.1.p | proliferating cellular nuclear antigen 1 | *Vigna unguiculata* | 8 | 8 | 29.981 | 0.004 | 3.246 |
| Down | Vigun11g134000.1.p | CDK-subunit 2 | *Vigna unguiculata* | 2 | 2 | 8.361 | 0.018 | 2.062 |
| Down | Vigun08g194200.1.p | STRUCTURAL MAINTENANCE OF CHROMOSOMES SMC FAMILY MEMBER | *Arabidopsis thaliana* | 6 | 4 | 25.457 | 0.002 | 3.292 |
| Down | Vigun07g076000.1.p | chloroplast RNA-binding protein 29 | *Arabidopsis thaliana* | 6 | 2 | 67.083 | 0.005 | 5.47 |
|  | **Cell structure and organization** | |  |  |  |  |  |  |
| Up | Vigun10g140600.1.p | PROTEIN CURVATURE THYLAKOID 1A, CHLOROPLASTIC | *Arabidopsis thaliana* | 6 | 5 | 36.340 | 0.004 | 2.011 |
| Down | Vigun02g172300.1.p | dTDP-4-dehydrorhamnose reductase / dTDP-6-deoxy-L-mannose dehydrogenase | *Arabidopsis thaliana* | 8 | 3 | 34.174 | 0.003 | 8.237 |
| Down | Vigun06g157500.1.p | plant UBX domain containing protein 4 | *Arabidopsis thaliana* | 7 | 2 | 37.093 | 0.009 | 3.018 |
| Down | Vigun06g157600.1.p | plant UBX domain containing protein 4 | *Arabidopsis thaliana* | 8 | 3 | 39.811 | 0.013 | 3.216 |
| Down | Vigun06g092600.1.p | PROTEIN TIC 40, CHLOROPLASTIC | *Arabidopsis thaliana* | 17 | 17 | 120.493 | 0.002 | 3.388 |
| Down | Vigun05g211800.1.p | FASCICLIN-like arabinogalactan 6 | *Arabidopsis thaliana* | 3 | 3 | 13.216 | 0.04 | 2.102 |
| Down | Vigun09g059800.1.p | reversibly glycosylated polypeptide 3 | *Vigna unguiculata* | 4 | 4 | 13.192 | 0.011 | 2.535 |
| Down | Vigun09g027000.1.p | villin 4 | *Vigna unguiculata* | 4 | 3 | 13.004 | 0.022 | 2.177 |
| Down | Vigun09g253800.1.p | tubulin beta-1 chain | *Vigna unguiculata* | 14 | 3 | 65.858 | 0.01 | 2.657 |
| Down | Vigun04g060800.1.p | protein SPIRAL1 and related proteins (SPR1) | *Vigna unguiculata* | 3 | 3 | 17.661 | 0.009 | 13.158 |
| Down | Vigun05g240500.2.p | histone deacetylase 2B | *Arabidopsis thaliana* | 3 | 3 | 8.169 | 0.010 | 3.741 |
| Down | Vigun05g202900.1.p | histone H2A 2 | *Arabidopsis thaliana* | 5 | 2 | 18.969 | 0.004 | 8.497 |
|  | **Chromatin organization** | |  |  |  |  |  |  |
| Down | Vigun09g260800.2.p | high mobility group B2 | *Arabidopsis thaliana* | 3 | 3 | 24.477 | 0.035 | 4.061 |
| Down | Vigun03g393100.1.p | nucleosome assembly protein 1;2 | *Vigna unguiculata* | 9 | 5 | 47.081 | 0.005 | 4.951 |
| Down | Vigun09g033900.1.p | nucleosome assembly protein 1;2 | *Vigna unguiculata* | 4 | 2 | 33.333 | 0.007 | 5.947 |
| Down | Vigun07g156200.1.p | HISTONE H1/H5 | *Vigna unguiculata* | 2 | 2 | 9.987 | 0.013 | 2.003 |
| Down | Vigun08g145000.1.p | HISTONE H1/H5 | *Vigna unguiculata* | 5 | 2 | 29.298 | 0.011 | 4.514 |
| Down | Vigun08g145400.1.p | HISTONE H1/H5 | *Vigna unguiculata* | 6 | 3 | 44.092 | 0.007 | 5.479 |
| Down | Vigun09g073800.1.p | template-activating factor I (SET, TAF1, I2PP2A) | *Vigna unguiculata* | 4 | 4 | 15.177 | 0.003 | 5.137 |
|  | **Hormone biosynthesis** | |  |  |  |  |  |  |
| Down | Vigun08g172400.1.p | OXIDOREDUCTASE, 2OG-FE II OXYGENASE FAMILY PROTEIN // 1-AMINOCYCLOPROPANE-1-CARBOXYLATE OXIDASE 3-RELATED | *Arabidopsis thaliana* | 3 | 3 | 7.397 | 0.007 | 2.085 |
|  | **Lipid metabolism** |  |  |  |  |  |  |  |
| Up | Vigun10g170100.1.p | lipoxygenase 1 | *Arabidopsis thaliana* | 16 | 16 | 79.154 | 0.002 | 4.522 |
| Up | Vigun11g163500.2.p | lipoxygenase 2 | *Arabidopsis thaliana* | 46 | 44 | 335.492 | 0.003 | 3.151 |
| Up | Vigun06g183700.1.p | GDSL-like Lipase/Acylhydrolase superfamily protein | *Arabidopsis thaliana* | 13 | 11 | 78.163 | 0.001 | 4.132 |
| Up | Vigun03g342000.2.p | glycerophosphoryl diester phosphodiesterase (E3.1.4.46, glpQ, ugpQ) | *Vigna unguiculata* | 3 | 2 | 24.261 | 0.033 | 2.431 |
| Up | Vigun06g056800.1.p | glycerophosphoryl diester phosphodiesterase (E3.1.4.46, glpQ, ugpQ) | *Vigna unguiculata* | 3 | 3 | 3.9642 | 0.044 | 2.115 |
| Up | Vigun05g241400.1.p | UDP-D-apiose/UDP-D-xylose synthase 2 | *Vigna unguiculata* | 14 | 3 | 100.993 | 0.025 | 6.492 |
| Up | Vigun02g079000.3.p | ATP-citrate lyase A-1 | *Arabidopsis thaliana* | 3 | 2 | 16.814 | 0.007 | 5.253 |
| Up | Vigun02g109800.1.p | nine-cis-epoxycarotenoid dioxygenase 4 | *Arabidopsis thaliana* | 2 | 2 | 16.556 | 0.012 | 5.411 |
| Down | Vigun03g031500.1.p | acetyl Co-enzyme a carboxylase biotin carboxylase subunit | *Vigna unguiculata* | 15 | 14 | 89.156 | 0.014 | 2.18 |
| Down | Vigun01g207200.1.p | ATP-citrate lyase A-3 | *Arabidopsis thaliana* | 5 | 3 | 14.703 | 0.009 | 3.352 |
| Down | Vigun11g127800.2.p | UDP-D-apiose/UDP-D-xylose synthase 2 | *Glycine max* | 17 | 6 | 112.334 | 0.0019 | 2.629 |
| Down | Vigun06g081500.1.p | catalytics;transferases;[acyl-carrier-protein] S-malonyltransferases;binding | *Arabidopsis thaliana* | 13 | 13 | 68.799 | 0.002 | 2.303 |
| Down | Vigun05g290600.1.p | CARBOXYLASE:PYRUVATE/ACETYL-COA/PROPIONYL-COA CARBOXYLASE | *Arabidopsis thaliana* | 4 | 4 | 35.497 | 0.002 | 4.353 |
| Down | Vigun01g231600.2.p | CARBOXYLASE:PYRUVATE/ACETYL-COA/PROPIONYL-COA CARBOXYLASE | *Arabidopsis thaliana* | 3 | 3 | 13.355 | 0.005 | 4.846 |
| Down | Vigun07g143400.1.p | alpha/beta-Hydrolases superfamily protein | *Arabidopsis thaliana* | 4 | 2 | 5.923 | 0.004 | 3.021 |
| Down | Vigun04g057800.1.p | biotin carboxyl carrier protein 2 | *Vigna unguiculata* | 6 | 5 | 10.892 | 0.019 | 3.298 |
| Down | Vigun05g134400.1.p | Acyl-ACP thioesterase | *Vigna unguiculata* | 6 | 6 | 35.029 | 0.004 | 2.253 |
| Down | Vigun10g012800.1.p | phospholipase A 2A | *Vigna unguiculata* | 7 | 7 | 71.345 | 0.031 | 2.111 |
| Down | Vigun10g189800.1.p | phospholipase D alpha 1 | *Vigna unguiculata* | 9 | 8 | 28.886 | 0.008 | 2.064 |
| Down | Vigun08g065300.1.p | rhamnose biosynthesis 1 | *Vigna unguiculata* | 9 | 3 | 24.190 | 0.014 | 2.657 |
| Down | Vigun08g205800.1.p | GLYCEROPHOSPHODIESTER PHOSPHODIESTERASE-LIKE PROTEIN-RELATED | *Vigna unguiculata* | 10 | 9 | 51.324 | 0.008 | 2.609 |
| Down | Vigun07g063300.1.p | Carotene epsilon-monooxygenase / LUT1 | *Arabidopsis thaliana* | 3 | 3 | 20.094 | 0.017 | 2.850 |
| Down | Vigun03g161100.1.p | geranylgeranyl reductase | *Vigna unguiculata* | 7 | 7 | 79.938 | 0.004 | 2.57 |
| Down | Vigun02g135600.1.p | geranylgeranyl pyrophosphate synthase 1 | *Vigna unguiculata* | 11 | 8 | 93.749 | 0.002 | 2.912 |
| Down | Vigun02g178500.1.p | 2-C-methyl-D-erythritol 2,4-cyclodiphosphate synthase / MECDP-synthase | *Vigna unguiculata* | 3 | 3 | 30.869 | 0.004 | 2.404 |
| Down | Vigun11g153800.1.p | UDP-XYL synthase 6 | *Vigna unguiculata* | 8 | 2 | 29.011 | 0.018 | 2.53 |
|  | **Membrane trafficking and intracellular transport** | | |  |  |  |  |  |
| Up | Vigun06g087100.1.p | Bifunctional inhibitor/lipid-transfer protein/seed storage 2S albumin superfamily protein | *Vigna unguiculata* | 2 | 2 | 21.27 | 0.032 | 2.569 |
| Up | Vigun03g154800.1.p | V-type H+-transporting ATPase subunit F (ATPeV1F, ATP6S14) | *Vigna unguiculata* | 2 | 2 | 10.58 | 0.003 | 2.142 |
| Up | Vigun03g313000.1.p | ATP synthase delta-subunit gene | *Vigna unguiculata* | 16 | 16 | 197.004 | 0.002 | 2.906 |
| Up | Vigun06g162100.1.p | ATPase, F1 complex, gamma subunit protein | *Vigna unguiculata* | 23 | 21 | 166.291 | 0.001 | 2.400 |
| Up | Vigun09g210600.1.p | F-type H+-transporting ATPase subunit b (ATPF0B, atpF) | *Vigna unguiculata* | 18 | 17 | 160.523 | 0.002 | 2.573 |
| Up | Vigun11g142500.1.p | plasma membrane intrinsic protein 2;4 | *Vigna unguiculata* | 2 | 2 | 12.352 | 0.002 | 4.871 |
| Up | VigunL069800.1.p | F-type H+-transporting ATPase subunit b (ATPF0B, atpF) | *Vigna unguiculata* | 15 | 13 | 79.464 | 0.001 | 2.526 |
| Up | Vigun05g258300.1.p | outer plastid envelope protein 16-1 | *Arabidopsis thaliana* | 2 | 2 | 14.992 | 0.003 | 4.006 |
| Up | Vigun08g129900.1.p | EXOCYST COMPLEX PROTEIN EXO70 | *Vigna unguiculata* | 5 | 2 | 5.275 | 0.003 | 15.628 |
| Up | Vigun08g157100.2.p | plasma membrane intrinsic protein 1;4 | *Vigna unguiculata* | 4 | 3 | 40.525 | 0.004 | 2.725 |
| Up | Vigun09g113900.1.p | PATELLIN-3-RELATED | *Vigna unguiculata* | 10 | 10 | 45.330 | 0.002 | 2.301 |
| Up | Vigun07g022400.1.p | protein transport protein SEC13 (SEC13) | *Vigna unguiculata* | 3 | 3 | 14.038 | 0.009 | 2.623 |
| Up | Vigun10g098200.1.p | lipid transfer protein 3 | *Vigna unguiculata* | 4 | 4 | 25.845 | 0.002 | 106.583 |
| Up | Vigun03g100800.1.p | IRON-SULFUR DOMAIN CONTAINING PROTEIN // PROTOCHLOROPHYLLIDE-DEPENDENT TRANSLOCON COMPONENT 52, CHLOROPLASTIC | *Arabidopsis thaliana* | 3 | 2 | 3.480 | 0.011 | 2.031 |
| Up | Vigun09g265000.1.p | coatomer protein complex, subunit gamma (COPG) | *Vigna unguiculata* | 5 | 3 | 9.386 | 0.037 | 2.359 |
| Up | Vigun07g018200.1.p | NIEMANN PICK TYPE C2 PROTEIN NPC2-RELATED // SUBFAMILY NOT NAMED | *Arabidopsis thaliana* | 3 | 3 | 26.851 | 0.023 | 2.037 |
| Up | Vigun10g183500.1.p | RAS-RELATED PROTEIN RABG3D | *Vigna unguiculata* | 5 | 2 | 26.316 | 0.007 | 3.684 |
| Down | Vigun01g242600.1.p | OXA1 // ALBINO3-LIKE PROTEIN 1, CHLOROPLASTIC | *Vigna unguiculata* | 3 | 3 | 13.665 | 0.018 | 2.064 |
| Down | Vigun09g276100.1.p | plasma membrane intrinsic protein 2;8 | *Vigna unguiculata* | 2 | 2 | 12.483 | 0.011 | 3.054 |
| Down | Vigun09g139400.1.p | nascent polypeptide-associated complex subunit alpha (EGD2, NACA) | *Arabidopsis thaliana* | 8 | 7 | 75.371 | 0.002 | 2.202 |
| Down | Vigun01g102000.1.p | nascent polypeptide-associated complex subunit alpha (EGD2, NACA) | *Arabidopsis thaliana* | 8 | 6 | 80.116 | 0.002 | 3.021 |
| Down | Vigun02g090100.1.p | MITOCHONDRIAL CARNITINE/ACYLCARNITINE CARRIER-LIKE PROTEIN | *Arabidopsis thaliana* | 4 | 2 | 10.609 | 0.026 | 2.576 |
| Down | Vigun05g256200.3.p | THO complex subunit 4 (THOC4, ALY) | *Arabidopsis thaliana* | 9 | 9 | 54.477 | 0.005 | 3.058 |
| Down | Vigun11g221900.1.p | signal recognition particle subunit SRP14 (SRP14) | *Vigna unguiculata* | 2 | 2 | 7.168 | 0.005 | 2.082 |
| Down | Vigun07g212400.1.p | Ran-binding protein 1 (RANBP1) | *Vigna unguiculata* | 6 | 5 | 24.257 | 0.006 | 3.149 |
| Down | Vigun11g169200.3.p | TRIGGER FACTOR CHAPERONE AND PEPTIDYL-PROLYL CIS/TRANS ISOMERASE | *Glycine max* | 7 | 6 | 46.923 | 0.005 | 2.619 |
| Down | Vigun11g178100.2.p | Medium subunit of clathrin adaptor complex | *Arabidopsis thaliana* | 8 | 7 | 36.67 | 0.007 | 3.008 |
|  | **Nitrogen and sulphur metabolism** | |  |  |  |  |  |  |
| Down | Vigun03g148800.1.p | urease accessory protein G | *Vigna unguiculata* | 3 | 2 | 15.164 | 0.005 | 3.99 |
| Down | Vigun04g066800.1.p | ATP SULFURYLASE 2 | *Vigna unguiculata* | 13 | 9 | 58.497 | 0.004 | 2.690 |
| Down | Vigun07g233800.1.p | ATP sulfurylase 1 | *Vigna unguiculata* | 7 | 4 | 23.474 | 0.003 | 2.227 |
| Up | Vigun10g027600.2.p | 2-oxo-4-hydroxy-4-carboxy-5-ureidoimidazoline decarboxylase / OHCU decarboxylase | *Vigna unguiculata* | 3 | 3 | 29.192 | 0.001 | 4.162 |
|  | **Nucleotide metabolism** | |  |  |  |  |  |  |
| Up | Vigun09g272400.1.p | methylthioadenosine nucleosidase 1 | *Vigna unguiculata* | 4 | 4 | 7.986 | 0.041 | 3.434 |
| Down | Vigun03g297500.1.p | ureidoglycine aminohydrolase | *Arabidopsis thaliana* | 3 | 2 | 17.580 | 0.016 | 2.938 |
| Down | Vigun07g168700.2.p | Uncharacterised conserved protein (UCP030210) | *Arabidopsis thaliana* | 5 | 5 | 25.377 | 0.001 | 2.020 |
| Down | Vigun03g064900.1.p | uridylate kinase (pyrH) | *Vigna unguiculata* | 4 | 4 | 10.658 | 0.001 | 13.019 |
| Down | Vigun07g075600.1.p | adenosine kinase 2 | *Vigna unguiculata* | 7 | 6 | 62.201 | 0.007 | 2.019 |
| Down | Vigun08g138400.1.p | dUTP diphosphatase / dUTPase | *Vigna unguiculata* | 3 | 3 | 19.886 | 0.003 | 10.772 |
| Down | Vigun11g221700.1.p | UMP/CMP kinase / Uridine monophosphate-cytidine monophosphate phosphotransferase // Nucleoside-diphosphate kinase / Nucleoside diphosphokinase | *Vigna unguiculata* | 5 | 5 | 21.255 | 0.002 | 3.45 |
|  | **Phenylpropanoid biosynthesis process** | |  |  |  |  |  |  |
| Down | Vigun05g174400.1.p | Chalcone-flavanone isomerase family protein | *Vigna unguiculata* | 5 | 5 | 17.542 | 0.011 | 2.281 |
| Down | Vigun07g288600.1.p | chalcone isomerase (E5.5.1.6) | *Vigna unguiculata* | 6 | 6 | 38.987 | 0.032 | 2.461 |
| Down | Vigun08g092000.1.p | flavonol synthase 1 | *Arabidopsis thaliana* | 5 | 4 | 12.701 | 0.002 | 2.784 |
|  | **Photosynthesis and photorespiration** | |  |  |  |  |  |  |
| Up | VigunL082400.1.p | NADH dehydrogenase subunit J | *Arabidopsis thaliana* | 3 | 3 | 15.23 | 0.002 | 4.617 |
| Up | VigunL002500.1.p | ribulose-bisphosphate carboxylases | *Arabidopsis thaliana* | 14 | 2 | 70.176 | 0.008 | 3.797 |
| Up | Vigun10g051600.2.p | rubisco activase | *Arabidopsis thaliana* | 33 | 6 | 427.912 | 0.001 | 32.726 |
| Up | Vigun01g004300.1.p | photosystem I subunit G | *Vigna unguiculata* | 7 | 6 | 49.589 | 0.008 | 8.067 |
| Up | Vigun03g145400.1.p | light-harvesting chlorophyll-protein complex II subunit B1 | *Vigna unguiculata* | 11 | 4 | 138.511 | 0.001 | 2.795 |
| Up | Vigun04g103500.1.p | photosystem I light harvesting complex gene 1 | *Vigna unguiculata* | 7 | 7 | 47.581 | 0.002 | 7.201 |
| Up | Vigun06g224500.1.p | light harvesting complex photosystem II subunit 6 | *Vigna unguiculata* | 9 | 8 | 78.429 | 0.003 | 4.784 |
| Up | Vigun05g056100.1.p | photosystem I subunit l | *Vigna unguiculata* | 5 | 5 | 80.675 | 0.008 | 3.64 |
| Up | Vigun09g075200.1.p | light-harvesting chlorophyll-protein complex I subunit A4 | *Vigna unguiculata* | 8 | 7 | 96.358 | 0.007 | 6.296 |
| Up | VigunL041500.1.p | photosystem II reaction center protein A | *Vigna unguiculata* | 12 | 12 | 125.016 | 0.003 | 4.414 |
| Up | Vigun06g030300.3.p | alanine:glyoxylate aminotransferase | *Arabidopsis thaliana* | 23 | 22 | 219.631 | 0.006 | 2.181 |
| Up | Vigun03g131500.1.p | alternative oxidase 2 | *Arabidopsis thaliana* | 2 | 2 | 8.699 | 0.005 | 3.338 |
| Up | Vigun10g152700.1.p | photosystem I P subunit | *Arabidopsis thaliana* | 3 | 3 | 10.008 | 0.011 | 2.664 |
| Up | Vigun01g226400.1.p | light harvesting complex photosystem II | *Vigna unguiculata* | 14 | 12 | 146.426 | 0.003 | 3.764 |
| Up | Vigun02g058000.1.p | PSBQ-LIKE PROTEIN 1, CHLOROPLASTIC | *Vigna unguiculata* | 7 | 7 | 44.630 | 0.001 | 2.927 |
| Up | Vigun03g133000.1.p | light harvesting complex photosystem II subunit 6 | *Vigna unguiculata* | 5 | 4 | 38.424 | 0.016 | 2.744 |
| Up | Vigun03g339900.1.p | photosystem I subunit F | *Vigna unguiculata* | 11 | 11 | 92.239 | 0.002 | 2.436 |
| Up | Vigun04g167600.1.p | light harvesting complex of photosystem II 5 | *Vigna unguiculata* | 19 | 8 | 232.785 | 0.002 | 4.258 |
| Up | Vigun05g222100.1.p | Uncharacterized protein | *Vigna unguiculata* | 5 | 4 | 31.584 | 0.002 | 2.109 |
| Up | Vigun06g094000.4.p | Oxygen evolving enhancer protein 3 (PsbQ) (PsbQ) | *Vigna unguiculata* | 2 | 2 | 9.37 | 0.017 | 3.536 |
| Up | Vigun07g168400.1.p | light harvesting complex of photosystem II 5 | *Vigna unguiculata* | 19 | 9 | 185.675 | 0.002 | 5.012 |
| Up | Vigun07g241100.1.p | photosystem I subunit D-2 | *Vigna unguiculata* | 21 | 20 | 214.249 | 0.002 | 2.03 |
| Up | Vigun07g285900.1.p | PsbP-like protein 2 | *Vigna unguiculata* | 5 | 4 | 32.459 | 0.002 | 3.122 |
| Up | Vigun08g189400.1.p | photosystem II subunit P-1 | *Vigna unguiculata* | 16 | 10 | 215.417 | 0.002 | 2.299 |
| Up | Vigun08g216300.1.p | photosystem II light harvesting complex gene 2.1 | *Vigna unguiculata* | 9 | 4 | 107.313 | 0.004 | 3.897 |
| Up | Vigun09g040000.1.p | photosystem I light harvesting complex gene 6 | *Vigna unguiculata* | 4 | 4 | 16.697 | 0.002 | 3.007 |
| Up | Vigun09g048600.1.p | photosystem I subunit K | *Vigna unguiculata* | 3 | 3 | 19.143 | 0.021 | 2.488 |
| Up | Vigun09g156000.1.p | PSBP DOMAIN-CONTAINING PROTEIN 4, CHLOROPLASTIC | *Vigna unguiculata* | 5 | 3 | 45.31 | 0.016 | 2.029 |
| Up | Vigun09g238500.1.p | light-harvesting complex I chlorophyll a/b binding protein 5 (LHCA5) | *Vigna unguiculata* | 3 | 3 | 23.222 | 0.005 | 2.092 |
| Up | Vigun10g078500.1.p | light harvesting complex photosystem II | *Vigna unguiculata* | 16 | 13 | 193.743 | 0.01 | 2.187 |
| Up | Vigun10g155200.1.p | photosystem I light harvesting complex gene 2 | *Vigna unguiculata* | 4 | 4 | 33.664 | 0.013 | 2.676 |
| Up | Vigun11g049900.1.p | light-harvesting chlorophyll B-binding protein 3 | *Vigna unguiculata* | 7 | 7 | 59.782 | 0.002 | 2.239 |
| Up | VigunL042900.1.p | photosystem II reaction center protein C | *Vigna unguiculata* | 19 | 15 | 199.003 | 0.006 | 3.008 |
| Up | VigunL043000.1.p | photosystem II reaction center protein D | *Vigna unguiculata* | 11 | 9 | 144.991 | 0.010 | 3.509 |
| Up | VigunL046800.1.p | iron-sulfur cluster binding;electron carriers;4 iron, 4 sulfur cluster binding | *Vigna unguiculata* | 4 | 3 | 29.693 | 0.005 | 3.406 |
| Up | VigunL062800.1.p | photosystem II reaction center protein E | *Vigna unguiculata* | 4 | 4 | 23.794 | 0.002 | 6.142 |
| Up | VigunL063200.1.p | photosynthetic electron transfer A | *Vigna unguiculata* | 17 | 15 | 160.548 | 0.003 | 2.125 |
| Up | VigunL063500.1.p | photosystem II reaction center protein B | *Vigna unguiculata* | 25 | 6 | 233.510 | 0.004 | 3.416 |
| Up | VigunL063700.1.p | photosystem II reaction center protein H | *Vigna unguiculata* | 6 | 5 | 76.785 | 0.011 | 8.786 |
| Up | Vigun03g096300.8.p | COMPLEX I INTERMEDIATE-ASSOCIATED PROTEIN 30 // NAD(P)-BINDING ROSSMANN-FOLD SUPERFAMILY PROTEIN | *Arabidopsis thaliana* | 9 | 8 | 44.207 | 0.002 | 2.134 |
| Up | Vigun09g160700.1.p | subunit NDH-M of NAD(P)H:plastoquinone dehydrogenase complex | *Arabidopsis thaliana* | 10 | 10 | 45.06 | 0.003 | 2.23 |
| Up | Vigun03g162000.4.p | (S)-2-hydroxy-acid oxidase / Hydroxy-acid oxidase B | *Vigna unguiculata* | 39 | 37 | 419.68 | 0.009 | 2.006 |
| Up | Vigun10g195900.2.p | NDH-dependent cyclic electron flow 1 | *Arabidopsis thaliana* | 3 | 3 | 25.887 | 0.002 | 3.236 |
| Up | VigunL079800.1.p | NAD(P)H dehydrogenase subunit H | *Arabidopsis thaliana* | 12 | 10 | 48.585 | 0.001 | 3.340 |
| Up | VigunL082300.1.p | photosystem II reaction center protein G | *Arabidopsis thaliana* | 3 | 2 | 8.477 | 0.002 | 3.784 |
| Up | Vigun07g010900.1.p | proton gradient regulation 5 | *Vigna unguiculata* | 3 | 3 | 12.124 | 0.005 | 3.280 |
| Up | Vigun07g285300.1.p | 2-oxoglutarate dehydrogenase, E1 component | *Vigna unguiculata* | 4 | 4 | 20.876 | 0.010 | 2.4182 |
| Up | VigunL082000.1.p | ribulose-bisphosphate carboxylases | *Vigna unguiculata* | 63 | 3 | 690.108 | 0.001 | 2.311 |
| Up | Vigun01g115300.1.p | magnesium chelatase subunit H (chlH, bchH) | *Vigna unguiculata* | 10 | 10 | 45.185 | 0.002 | 3.014 |
| Up | Vigun03g111900.1.p | Catechol oxidase / Tyrosinase // Tyrosinase / Tyrosine-dopa oxidase | *Vigna unguiculata* | 7 | 4 | 27.846 | 0.002 | 6.516 |
| Down | Vigun02g184700.1.p | hydroxymethylbilane synthase | *Arabidopsis thaliana* | 20 | 16 | 152.504 | 0.011 | 2.047 |
| Down | Vigun08g221600.2.p | Coproporphyrinogen III oxidase | *Arabidopsis thaliana* | 19 | 17 | 125.434 | 0.004 | 2.339 |
| Down | Vigun07g083800.1.p | Tetratricopeptide repeat (TPR)-like superfamily protein | *Arabidopsis thaliana* | 5 | 4 | 24.302 | 0.019 | 2.539 |
| Down | Vigun08g126400.1.p | proton gradient regulation 7 | *Arabidopsis thaliana* | 4 | 3 | 8.542 | 0.042 | 5.281 |
| Down | Vigun02g183600.1.p | ALBINA 1 | *Vigna unguiculata* | 8 | 7 | 40.442 | 0.008 | 2.500 |
| Down | Vigun03g173900.1.p | magnesium chelatase subunit I (chlI, bchI) | *Vigna unguiculata* | 24 | 23 | 207.038 | 0.002 | 3.026 |
| Down | Vigun06g090900.2.p | Uncharacterized protein | *Arabidopsis thaliana* | 9 | 9 | 74.934 | 0.002 | 2.204 |
| Down | Vigun09g276500.1.p | glutamate-1-semialdehyde 2,1-aminomutase 2 | *Arabidopsis thaliana* | 15 | 15 | 152.787 | 0.002 | 2.386 |
| Down | Vigun09g163800.1.p | Porphobilinogen synthase / Delta-aminolevulinic acid dehydratase | *Vigna unguiculata* | 19 | 18 | 151.017 | 0.002 | 2.091 |
| Down | Vigun11g165700.1.p | enzyme binding;tetrapyrrole binding | *Arabidopsis thaliana* | 5 | 5 | 40.659 | 0.008 | 3.892 |
| Down | Vigun01g233300.1.p | Uroporphyrinogen decarboxylase | *Arabidopsis thaliana* | 13 | 13 | 105.973 | 0.004 | 2.716 |
| Up | Vigun06g110200.1.p | Rubredoxin (Rubredoxin) | *Arabidopsis thaliana* | 3 | 3 | 20.025 | 0.048 | 2.234 |
|  | **Protein processing** | |  |  |  |  |  |  |
| Up | Vigun03g347500.1.p | PEPTIDYL-PROLYL CIS-TRANS ISOMERASE // CYCLOPHILIN-LIKE PEPTIDYL-PROLYL CIS-TRANS ISOMERASE FAMILY PROTEIN | *Vigna unguiculata* | 6 | 6 | 46.171 | 0.003 | 2.126 |
| Up | Vigun10g040700.1.p | rotamase FKBP 1 | *Vigna unguiculata* | 10 | 9 | 39.195 | 0.009 | 2.171 |
| Up | Vigun01g143800.1.p | oligosaccharyltransferase complex subunit delta (ribophorin II) (SWP1, RPN2) | *Vigna unguiculata* | 2 | 2 | 2.488 | 0.013 | 3.731 |
| Up | Vigun07g213600.1.p | one-helix protein 2 | *Arabidopsis thaliana* | 5 | 3 | 30.731 | 0.013 | 2.744 |
| Up | Vigun01g026200.1.p | RNA binding protein | *Arabidopsis thaliana* | 5 | 5 | 27.785 | 0.031 | 2.039 |
| Up | Vigun08g119200.1.p | ditrans,polycis-polyprenyl diphosphate synthase (DHDDS, RER2, SRT1) | *Arabidopsis thaliana* | 8 | 8 | 61.656 | 0.012 | 3.59 |
| Up | Vigun04g184000.2.p | Vacuolar ATPase assembly integral membrane protein VMA21-like domain | *Vigna unguiculata* | 14 | 10 | 86.432 | 0.028 | 3.103 |
| Up | Vigun07g149400.1.p | thylakoid lumen 18.3 kDa protein | *Arabidopsis thaliana* | 8 | 8 | 85.623 | 0.002 | 2.731 |
| Up | Vigun01g155500.1.p | Glutathione S-transferase family protein | *Arabidopsis thaliana* | 11 | 7 | 111.003 | 0.006 | 3.252 |
| Up | Vigun06g090400.1.p | oxidoreductases, acting on NADH or NADPH, quinone or similar compound as acceptor | *Arabidopsis thaliana* | 4 | 4 | 34.649 | 0.002 | 4.142 |
| Down | Vigun01g155700.1.p | GLUTATHIONE S-TRANSFERASE, GST, SUPERFAMILY, GST DOMAIN CONTAINING | *Arabidopsis thaliana* | 7 | 2 | 32.606 | 0.026 | 2.66 |
| Down | Vigun09g268400.1.p | small subunit ribosomal protein S10e (RP-S10e, RPS10) | *Arabidopsis thaliana* | 11 | 3 | 100.427 | 0.031 | 2.757 |
| Down | Vigun03g041700.1.p | small subunit ribosomal protein S10e (RP-S10e, RPS10) | *Arabidopsis thaliana* | 10 | 2 | 92.866 | 0.024 | 3.37 |
| Down | Vigun02g178200.1.p | small subunit ribosomal protein S10e (RP-S10e, RPS10) | *Arabidopsis thaliana* | 11 | 4 | 94.580 | 0.008 | 4.564 |
| Down | Vigun03g269000.2.p | RUB1 conjugating enzyme 1 | *Arabidopsis thaliana* | 2 | 2 | 3.518 | 0.013 | 2.488 |
| Down | Vigun10g040800.1.p | FK506-binding protein 2 | *Vigna unguiculata* | 3 | 3 | 43.149 | 0.001 | 2.907 |
| Down | Vigun06g055300.1.p | homolog of bacterial cytokinesis Z-ring protein FTSZ 1-1 | *Vigna unguiculata* | 11 | 7 | 60.887 | 0.007 | 2.847 |
| Down | Vigun03g399900.2.p | chaperonin 20 | *Vigna unguiculata* | 14 | 9 | 120.655 | 0.001 | 7.588 |
| Down | Vigun05g136800.2.p | rotamase FKBP 1 | *Vigna unguiculata* | 4 | 3 | 14.124 | 0.032 | 2.048 |
| Down | Vigun03g013100.1.p | ubiquitin-conjugating enzyme E2 variant (UBE2V) | *Arabidopsis thaliana* | 9 | 9 | 47.779 | 0.001 | 2.355 |
| Down | Vigun04g160200.1.p | ubiquiting-conjugating enzyme 2 | *Arabidopsis thaliana* | 2 | 2 | 17.458 | 0.003 | 2.191 |
| Down | Vigun01g095800.2.p | GRPE PROTEIN HOMOLOG | *Vigna unguiculata* | 15 | 13 | 138.577 | 0.008 | 2.016 |
| Down | Vigun03g196200.3.p | CHAPERONIN // CHAPERONIN 60 SUBUNIT ALPHA 2, CHLOROPLASTIC | *Vigna unguiculata* | 40 | 37 | 398.654 | 0.02 | 2.161 |
| Down | Vigun04g185200.1.p | CALCIUM HOMEOSTASIS REGULATOR-RELATED | *Arabidopsis thaliana* | 3 | 3 | 12.818 | 0.002 | 5.22 |
| Down | Vigun06g042300.1.p | CHAPERONIN 60 SUBUNIT BETA 4, CHLOROPLASTIC | *Vigna unguiculata* | 48 | 41 | 502.731 | 0.005 | 2.082 |
| Down | Vigun07g050000.1.p | heat shock protein 60 | *Vigna unguiculata* | 25 | 4 | 157.595 | 0.006 | 2.117 |
| Down | Vigun07g140400.1.p | calreticulin 1a | *Vigna unguiculata* | 13 | 12 | 85.175 | 0.012 | 3.015 |
| Down | Vigun07g166500.1.p | TCP-1/cpn60 chaperonin family protein | *Vigna unguiculata* | 3 | 3 | 16.930 | 0.036 | 2.195 |
| Down | Vigun08g206000.1.p | tubulin folding cofactor A (KIESEL) | *Vigna unguiculata* | 5 | 4 | 17.337 | 0.024 | 2.631 |
| Down | Vigun09g217400.3.p | HOPW1-1-interacting 2 | *Vigna unguiculata* | 3 | 3 | 7.806 | 0.009 | 2.067 |
| Down | Vigun10g133400.1.p | MAP kinase 4 | *Vigna unguiculata* | 4 | 2 | 3.676 | 0.03 | 2.486 |
| Down | Vigun01g086800.1.p | H/ACA ribonucleoprotein complex subunit 1 (GAR1, NOLA1) | *Vigna unguiculata* | 3 | 3 | 11.636 | 0.01 | 3.651 |
| Down | Vigun10g018700.1.p | 10 KDA HEAT SHOCK PROTEIN // CHLOROPLAST CHAPERONIN 10 | *Vigna unguiculata* | 8 | 8 | 71.6561 | 0.001 | 2.166 |
| Down | Vigun07g159300.1.p | RUBISCO ACCUMULATION FACTOR 1, CHLOROPLASTIC-RELATED | *Arabidopsis thaliana* | 11 | 9 | 74.928 | 0.004 | 2.545 |
| Down | Vigun07g061800.1.p | cell division protein FtsZ (ftsZ) | *Vigna unguiculata* | 8 | 3 | 57.338 | 0.01 | 3.291 |
|  | **Protein degradation** | |  |  |  |  |  |  |
| Up | Vigun07g035800.1.p | S-formylglutathione hydrolase | *Vigna unguiculata* | 6 | 6 | 22.833 | 0.003 | 2.958 |
| Up | Vigun07g203500.1.p | serine carboxypeptidase-like 27 | *Vigna unguiculata* | 10 | 10 | 118.318 | 0.002 | 3.118 |
| Up | Vigun02g200500.1.p | ZINC METALLOPEPTIDASE EGY3, CHLOROPLASTIC-RELATED | *Vigna unguiculata* | 7 | 5 | 30.509 | 0.002 | 6.509 |
| Up | Vigun08g158300.3.p | ZINC METALLOPROTEASE EGY2, CHLOROPLASTIC-RELATED | *Vigna unguiculata* | 3 | 3 | 16.533 | 0.038 | 2.271 |
| Up | Vigun05g182600.1.p | gamma-glutamyl hydrolase 2 | *Arabidopsis thaliana* | 2 | 2 | 19.194 | 0.004 | 2.431 |
| Up | Vigun01g204500.1.p | Cathepsin B / Cathepsin B1 | *Vigna unguiculata* | 3 | 3 | 14.466 | 0.007 | 2.024 |
| Up | Vigun04g188700.1.p | DegP protease 1 | *Vigna unguiculata* | 11 | 11 | 103.165 | 0.002 | 3.226 |
| Up | Vigun06g049100.1.p | Bleomycin hydrolase / Aminopeptidase C (Lactococcus lactis) | *Arabidopsis thaliana* | 2 | 2 | 10.145 | 0.009 | 2.074 |
| Up | Vigun07g197800.2.p | SERINE CARBOXYPEPTIDASE-LIKE 49 | *Vigna unguiculata* | 2 | 2 | 7.112 | 0.006 | 3.299 |
| Up | Vigun07g225100.1.p | tripeptidyl peptidase ii | *Vigna unguiculata* | 11 | 11 | 48.648 | 0.006 | 2.099 |
| Up | Vigun10g060300.1.p | Serine carboxypeptidases (lysosomal cathepsin A) // Serine carboxypeptidases | *Vigna unguiculata* | 2 | 2 | 15.682 | 0.002 | 2.595 |
| Up | Vigun10g151400.1.p | PROPROTEIN CONVERTASE SUBTILISIN/KEXIN | *Vigna unguiculata* | 4 | 4 | 27.511 | 0.013 | 3.561 |
| Up | Vigun10g158900.1.p | PROPROTEIN CONVERTASE SUBTILISIN/KEXIN | *Vigna unguiculata* | 17 | 15 | 152.776 | 0.002 | 2.208 |
| Up | Vigun07g135500.1.p | aspartic proteinase A1 | *Vigna unguiculata* | 3 | 2 | 17.972 | 0.009 | 3.926 |
| Up | Vigun06g023000.1.p | cystatin B | *Vigna unguiculata* | 5 | 5 | 45.869 | 0.002 | 3.326 |
| Up | Vigun10g065000.1.p | cystatin B | *Glycine max* | 4 | 2 | 24.351 | 0.004 | 8.840 |
| Down | Vigun03g371600.1.p | Leucyl aminopeptidase / Peptidase S | *Vigna unguiculata* | 11 | 6 | 60.703 | 0.017 | 2.054 |
| Down | Vigun07g045200.1.p | SERINE PROTEASE FAMILY S10 SERINE CARBOXYPEPTIDASE | *Vigna unguiculata* | 3 | 3 | 18.860 | 0.033 | 2.266 |
| Down | Vigun09g184800.1.p | ASPARTYL PROTEASE-LIKE PROTEIN | *Vigna unguiculata* | 5 | 3 | 14.565 | 0.010 | 4.492 |
| Down | Vigun08g107400.1.p | ASPARTYL PROTEASES // ASPARTYL PROTEASE-LIKE PROTEIN | *Vigna unguiculata* | 2 | 2 | 14.530 | 0.046 | 5.458 |
| Down | Vigun10g156100.1.p | regulatory particle triple-A 1A | *Vigna unguiculata* | 6 | 6 | 24.451 | 0.019 | 2.127 |
| Down | Vigun08g153700.1.p | Subtilisin-like serine endopeptidase family protein | *Vigna unguiculata* | 7 | 7 | 47.451 | 0.035 | 3.516 |
| Down | Vigun10g198500.1.p | regulatory particle triple-A ATPase 6A | *Vigna unguiculata* | 3 | 3 | 11.033 | 0.004 | 3.701 |
| Down | Vigun01g150300.1.p | regulatory particle AAA-ATPase 2A | *Vigna unguiculata* | 4 | 3 | 11.0939 | 0.014 | 2.345 |
|  | **Protein synthesis** |  |  |  |  |  |  |  |
| Up | Vigun04g202600.1.p | ribosomal protein L22 | *Vigna unguiculata* | 8 | 8 | 33.875 | 0.011 | 3.514 |
| Down | Vigun10g170700.1.p | eif4a-2 | *Arabidopsis thaliana* | 13 | 4 | 73.861 | 0.017 | 2.306 |
| Down | Vigun11g189700.1.p | glycine-rich protein | *Arabidopsis thaliana* | 9 | 9 | 26.822 | 0.016 | 5.217 |
| Down | Vigun11g156600.1.p | plastid-specific ribosomal protein 4 | *Arabidopsis thaliana* | 5 | 4 | 31.852 | 0.031 | 2.032 |
| Down | Vigun09g056100.1.p | EUKARYOTIC TRANSLATION INITIATION FACTOR 4 GAMMA // SUBFAMILY NOT NAMED | *Arabidopsis thaliana* | 15 | 12 | 71.658 | 0.005 | 2.784 |
| Down | Vigun08g151900.1.p | Mitochondrial glycoprotein family protein | *Arabidopsis thaliana* | 2 | 2 | 20.236 | 0.002 | 2.306 |
| Down | Vigun07g032300.1.p | TRANSLATION FACTOR // TYPA-LIKE TRANSLATION ELONGATION FACTOR SVR3-RELATED | *Arabidopsis thaliana* | 6 | 6 | 28.411 | 0.015 | 2.464 |
| Down | Vigun03g080100.1.p | eukaryotic translation initiation factor 4B1 | *Arabidopsis thaliana* | 20 | 19 | 87.518 | 0.028 | 3.146 |
| Down | Vigun07g217800.2.p | eukaryotic translation initiation factor 2 (eIF-2) family protein | *Vigna unguiculata* | 3 | 3 | 11.408 | 0.011 | 3.733 |
| Down | Vigun11g221300.1.p | Plant invertase/pectin methylesterase inhibitor superfamily protein | *Vigna unguiculata* | 3 | 2 | 6.726 | 0.006 | 3.274 |
| Down | Vigun01g048000.1.p | 40s ribosomal protein SA B | *Vigna unguiculata* | 8 | 3 | 48.243 | 0.012 | 2.421 |
| Down | Vigun01g062100.1.p | Translation initiation factor 2, small GTP-binding protein | *Vigna unguiculata* | 25 | 24 | 127.12 | 0.007 | 3.03 |
| Down | Vigun01g068500.1.p | 28S RIBOSOMAL PROTEIN S21, MITOCHONDRIAL | *Vigna unguiculata* | 4 | 4 | 43.414 | 0.007 | 2.037 |
| Down | Vigun01g171800.1.p | large subunit ribosomal protein LP2 (RP-LP2, RPLP2) | *Vigna unguiculata* | 2 | 2 | 23.265 | 0.010 | 6.551 |
| Down | Vigun01g192700.3.p | large subunit ribosomal protein L18e (RP-L18e, RPL18) | *Vigna unguiculata* | 9 | 3 | 47.295 | 0.015 | 2.088 |
| Down | Vigun02g054900.1.p | eukaryotic elongation factor 5A-1 | *Vigna unguiculata* | 8 | 2 | 53.089 | 0.007 | 2.333 |
| Down | Vigun02g081500.1.p | large subunit ribosomal protein L27Ae (RP-L27Ae, RPL27A) | *Vigna unguiculata* | 5 | 2 | 38.069 | 0.006 | 2.311 |
| Down | Vigun02g151500.1.p | large subunit ribosomal protein L26e (RP-L26e, RPL26) | *Vigna unguiculata* | 10 | 4 | 43.023 | 0.017 | 2.035 |
| Down | Vigun02g166000.1.p | small subunit ribosomal protein S30e (RP-S30e, RPS30) | *Vigna unguiculata* | 3 | 3 | 13.656 | 0.007 | 2.262 |
| Down | Vigun03g319600.1.p | TRANSLATION FACTOR | *Vigna unguiculata* | 27 | 8 | 226.065 | 0.003 | 3.408 |
| Down | Vigun03g320900.1.p | arge subunit ribosomal protein L27Ae (RP-L27Ae, RPL27A) | *Vigna unguiculata* | 6 | 3 | 34.982 | 0.002 | 2.107 |
| Down | Vigun03g374400.1.p | Ribosomal protein L35 | *Vigna unguiculata* | 2 | 2 | 10.818 | 0.013 | 2.022 |
| Down | Vigun04g106200.1.p | ribosomal protein S27 | *Vigna unguiculata* | 5 | 5 | 34.011 | 0.008 | 2.224 |
| Down | Vigun05g136300.1.p | small subunit ribosomal protein S20e (RP-S20e, RPS20) | *Vigna unguiculata* | 6 | 5 | 28.522 | 0.004 | 2.856 |
| Down | Vigun05g137100.1.p | small subunit ribosomal protein S8e (RP-S8e, RPS8) | *Vigna unguiculata* | 10 | 2 | 87.515 | 0.039 | 3.333 |
| Down | Vigun05g210500.2.p | large subunit ribosomal protein L37e (RP-L37e, RPL37) | *Vigna unguiculata* | 3 | 3 | 3.851 | 0.010 | 3.723 |
| Down | Vigun05g238400.1.p | 60S ACIDIC RIBOSOMAL PROTEIN FAMILY MEMBER // 60S ACIDIC RIBOSOMAL PROTEIN P2-4 | *Vigna unguiculata* | 9 | 9 | 97.943 | 0.024 | 2.172 |
| Down | Vigun05g277400.1.p | large subunit ribosomal protein L28e (RP-L28e, RPL28) | *Vigna unguiculata* | 8 | 7 | 43.559 | 0.003 | 2.462 |
| Down | Vigun05g284300.1.p | ribosomal protein S17 | *Vigna unguiculata* | 4 | 3 | 22.229 | 0.002 | 2.137 |
| Down | Vigun05g299300.2.p | small subunit ribosomal protein S12e (RP-S12e, RPS12) | *Vigna unguiculata* | 5 | 5 | 49.894 | 0.003 | 2.041 |
| Down | Vigun06g199900.1.p | Ribosomal protein S6e | *Vigna unguiculata* | 13 | 3 | 57.475 | 0.005 | 2.484 |
| Down | Vigun07g021600.1.p | small subunit ribosomal protein S28e (RP-S28e, RPS28) | *Vigna unguiculata* | 3 | 3 | 17.963 | 0.004 | 2.342 |
| Down | Vigun07g045400.1.p | plastid ribosomal protein l11 | *Vigna unguiculata* | 9 | 9 | 94.249 | 0.001 | 2.644 |
| Down | Vigun07g058200.1.p | Ribosomal S17 family protein | *Vigna unguiculata* | 10 | 5 | 75.977 | 0.005 | 2.373 |
| Down | Vigun07g058900.1.p | large subunit ribosomal protein LP2 (RP-LP2, RPLP2) | *Vigna unguiculata* | 3 | 3 | 38.155 | 0.038 | 2.069 |
| Down | Vigun07g083100.2.p | large subunit ribosomal protein L35e (RP-L35e, RPL35) | *Vigna unguiculata* | 5 | 5 | 32.283 | 0.004 | 2.788 |
| Down | Vigun07g204200.1.p | elongation factor 1-alpha (EEF1A) | *Vigna unguiculata* | 18 | 3 | 179.546 | 0.001 | 3.218 |
| Down | Vigun07g224500.1.p | large subunit ribosomal protein L36e (RP-L36e, RPL36) | *Vigna unguiculata* | 5 | 4 | 28.262 | 0.013 | 2.048 |
| Down | Vigun07g253400.1.p | small subunit ribosomal protein S29e (RP-S29e, RPS29) | *Vigna unguiculata* | 3 | 3 | 5.121 | 0.007 | 2.326 |
| Down | Vigun08g008100.1.p | ELONGATION FACTOR 1-BETA | *Vigna unguiculata* | 13 | 4 | 92.579 | 0.005 | 2.08 |
| Down | Vigun08g013400.3.p | Ribosomal protein L31 | *Vigna unguiculata* | 9 | 8 | 88.172 | 0.003 | 2.444 |
| Down | Vigun08g161500.1.p | Ribosomal protein S26e family protein | *Vigna unguiculata* | 5 | 3 | 27.948 | 0.016 | 2.016 |
| Down | Vigun09g033400.1.p | Translation initiation factor SUI1 family protein | *Vigna unguiculata* | 2 | 2 | 8.6306 | 0.004 | 3.284 |
| Down | Vigun10g009800.1.p | ribosomal protein L4 | *Vigna unguiculata* | 18 | 18 | 140.091 | 0.005 | 2.062 |
| Down | Vigun10g040600.1.p | small subunit ribosomal protein S8e (RP-S8e, RPS8) | *Vigna unguiculata* | 12 | 4 | 102.145 | 0.01 | 2.116 |
| Down | Vigun10g176500.1.p | 60S RIBOSOMAL PROTEIN L28 | *Vigna unguiculata* | 8 | 7 | 55.086 | 0.0019 | 2.904 |
| Down | Vigun10g188500.1.p | ubiquitin 6 | *Vigna unguiculata* | 9 | 2 | 81.858 | 0.043 | 2.664 |
| Down | Vigun11g012400.1.p | Ribosome recycling factor | *Vigna unguiculata* | 2 | 2 | 6.333 | 0.019 | 2.334 |
| Down | Vigun11g115300.1.p | translation initiation factor 1 (EIF1, SUI1) | *Vigna unguiculata* | 2 | 2 | 2.728 | 0.031 | 2.449 |
| Down | VigunL039500.1.p | ribosomal protein L32 | *Vigna unguiculata* | 3 | 2 | 12.281 | 0.008 | 2.047 |
| Down | Vigun08g135500.1.p | eukaryotic translation initiation factor 2 beta subunit | *Vigna unguiculata* | 4 | 4 | 17.460 | 0.014 | 2.093 |
| Down | Vigun07g133800.1.p | Glutamyl/glutaminyl-tRNA synthetase, class Ic | *Vigna unguiculata* | 3 | 2 | 19.727 | 0.009 | 2.989 |
| Down | Vigun07g144000.1.p | large subunit ribosomal protein L22e (RP-L22e, RPL22) | *Vigna unguiculata* | 6 | 2 | 29.191 | 0.002 | 3.202 |
| Down | Vigun07g011000.1.p | Eukaryotic initiation factor 4E protein | *Vigna unguiculata* | 5 | 5 | 22.967 | 0.005 | 2.431 |
| Down | Vigun06g168800.1.p | TRANSLATION FACTOR | *Vigna unguiculata* | 10 | 8 | 52.736 | 0.008 | 2.092 |
| Down | Vigun11g223300.1.p | translation initiation factor 3 subunit I (EIF3I) | *Arabidopsis thaliana* | 7 | 7 | 33.118 | 0.004 | 2.221 |
| Down | Vigun01g156600.1.p | large subunit ribosomal protein L4e (RP-L4e, RPL4) | *Vigna unguiculata* | 12 | 7 | 82.895 | 0.012 | 2.679 |
|  | **Signal transduction** | |  |  |  |  |  |  |
| Up | Vigun01g007900.1.p | Leucine-rich repeat (LRR) family protein | *Arabidopsis thaliana* | 7 | 6 | 35.471 | 0.012 | 3.746 |
| Down | Vigun09g178000.1.p | general regulatory factor 2 | *Arabidopsis thaliana* | 21 | 5 | 153.625 | 0.008 | 2.01 |
| Down | Vigun10g009700.1.p | RAS 5 | *Vigna unguiculata* | 8 | 2 | 46.154 | 0.038 | 2.773 |
| Down | Vigun08g077600.2.p | ADP-ribosylation factor family (Arf) // Ras of Complex, Roc, domain of DAPkinase (Roc) | *Vigna unguiculata* | 3 | 2 | 19.89 | 0.025 | 2.040 |
|  | **Stress, defense and homeostatic response: defense response** | | |  |  |  |  |  |
| Up | Vigun05g142900.1.p | drought-repressed 4 | *Arabidopsis thaliana* | 5 | 4 | 25.581 | 0.009 | 26.470 |
| Up | Vigun05g143200.1.p | drought-repressed 4 | *Arabidopsis thaliana* | 8 | 7 | 94.554 | 0.023 | 9.464 |
| Up | Vigun05g144000.1.p | drought-repressed 4 | *Arabidopsis thaliana* | 6 | 2 | 41.858 | 0.006 | 11.766 |
| Up | Vigun05g143700.1.p | kunitz trypsin inhibitor 1 | *Arabidopsis thaliana* | 3 | 3 | 33.625 | 0.029 | 2.680 |
| Up | Vigun05g143800.1.p | kunitz trypsin inhibitor 1 | *Arabidopsis thaliana* | 4 | 4 | 21.014 | 0.033 | 3.304 |
| Up | Vigun05g145700.1.p | kunitz trypsin inhibitor 1 | *Arabidopsis thaliana* | 7 | 5 | 89.618 | 0.003 | 4.585 |
| Up | Vigun05g146000.1.p | kunitz trypsin inhibitor 1 | *Arabidopsis thaliana* | 11 | 9 | 129.782 | 0.007 | 5.835 |
| Up | Vigun05g146100.1.p | kunitz trypsin inhibitor 1 | *Arabidopsis thaliana* | 10 | 6 | 104.922 | 0.002 | 20.902 |
| Up | Vigun05g143400.1.p | Kunitz family trypsin and protease inhibitor protein | *Arabidopsis thaliana* | 4 | 4 | 67.556 | 0.024 | 2.607 |
| Up | Vigun05g145900.1.p | Kunitz family trypsin and protease inhibitor protein | *Arabidopsis thaliana* | 13 | 10 | 168.947 | 0.033 | 9.852 |
| Up | Vigun01g031900.1.p | NADPH:quinone oxidoreductase | *Arabidopsis thaliana* | 4 | 2 | 28.367 | 0.013 | 4.573 |
| Up | Vigun09g131100.1.p | manganese superoxide dismutase 1 | *Vigna unguiculata* | 8 | 6 | 59.536 | 0.004 | 2.399 |
| Up | Vigun09g243000.1.p | allene oxide synthase | *Arabidopsis thaliana* | 13 | 13 | 61.4106 | 0.007 | 4.781 |
| Up | Vigun07g173500.1.p | Plastid-lipid associated protein PAP / PAP_fibrillin | *Arabidopsis thaliana* | 9 | 9 | 95.335 | 0.001 | 2.268 |
| Up | Vigun11g030900.1.p | Pathogenesis-related protein Bet v I family (Bet_v_1) | *Vigna unguiculata* | 5 | 3 | 39.361 | 0.003 | 13.455 |
| Up | Vigun11g030700.1.p | Pathogenesis-related protein Bet v I family (Bet_v_1) | *Vigna unguiculata* | 7 | 3 | 37.029 | 0.008 | 3.684 |
| Down | Vigun03g418300.1.p | Pathogenesis-related protein Bet v I family (Bet_v_1) | *Vigna unguiculata* | 3 | 2 | 7.067 | 0.002 | 4.453 |
| Down | Vigun09g009700.1.p | Pathogenesis-related protein Bet v I family (Bet_v_1) | *Vigna unguiculata* | 3 | 3 | 6.499 | 0.004 | 9.29 |
| Down | Vigun09g010000.1.p | Pathogenesis-related protein Bet v I family (Bet_v_1) | *Vigna unguiculata* | 19 | 18 | 145.773 | 0.005 | 4.435 |
| Down | Vigun11g032100.1.p | Pathogenesis-related protein Bet v I family (Bet_v_1) | *Vigna unguiculata* | 8 | 8 | 34.193 | 0.024 | 2.05 |
| Down | Vigun03g418400.1.p | Pathogenesis-related protein Bet v I family (Bet_v_1) | *Vigna unguiculata* | 6 | 5 | 29.61 | 0.006 | 2.361 |
| Down | Vigun08g179200.3.p | RNA-binding (RRM/RBD/RNP motifs) family protein | *Arabidopsis thaliana* | 2 | 2 | 4.27 | 0.039 | 2.249 |
| Down | Vigun03g396400.1.p | Stress responsive alpha-beta barrel domain protein | *Arabidopsis thaliana* | 5 | 5 | 47.594 | 0.023 | 2.330 |
| Down | Vigun01g215400.1.p | ALWAYS EARLY 4 | *Arabidopsis thaliana* | 6 | 6 | 40.557 | 0.02 | 2.064 |
| Down | Vigun03g152600.1.p | lysm domain GPI-anchored protein 2 precursor | *Arabidopsis thaliana* | 2 | 2 | 12.858 | 0.002 | 6.827 |
| Down | Vigun03g252000.1.p | basic chitinase |  | 2 | 2 | 13.766 | 0.007 | 3.922 |
|  | **Stress, defense and homeostatic response: heat stress response** | | |  |  |  |  |  |
| Up | Vigun03g204800.1.p | nodulin-related protein 1 | *Arabidopsis thaliana* | 8 | 7 | 55.851 | 0.039 | 4.012 |
| Up | Vigun03g077600.2.p | MOLECULAR CHAPERONE DNAJ | *Vigna unguiculata* | 7 | 2 | 27.174 | 0.012 | 2.224 |
| Up | Vigun09g068700.1.p | temperature-induced lipocalin | *Arabidopsis thaliana* | 2 | 2 | 4.612 | 0.033 | 2.125 |
| Up | Vigun09g222500.1.p | 17.6 kDa class II heat shock protein | *Arabidopsis thaliana* | 10 | 8 | 88.465 | 0.009 | 8.884 |
| Up | Vigun08g042400.1.p | 17.6 kDa class II heat shock protein | *Arabidopsis thaliana* | 7 | 6 | 64.084 | 0.029 | 7.286 |
| Up | Vigun01g190300.1.p | heat shock factor binding protein | *Arabidopsis thaliana* | 3 | 3 | 11.66 | 0.005 | 2.67 |
| Up | Vigun03g340700.2.p | heat shock protein 101 | *Vigna unguiculata* | 14 | 9 | 82.100 | 0.009 | 8.430 |
| Up | Vigun04g168600.1.p | 22.0 KDA HEAT SHOCK PROTEIN | *Arabidopsis thaliana* | 2 | 2 | 10.358 | 0.038 | 4.372 |
| Up | Vigun08g155100.1.p | 17.6 KDA CLASS I HEAT SHOCK PROTEIN 1-RELATED | *Arabidopsis thaliana* | 20 | 14 | 136.531 | 0.011 | 20.79 |
| Up | Vigun03g104500.1.p | SMALL HEAT-SHOCK PROTEIN HSP20 FAMILY // 17.6 KDA CLASS I HEAT SHOCK PROTEIN 1-RELATED | *Arabidopsis thaliana* | 5 | 3 | 30.675 | 0.023 | 5.877 |
| Up | Vigun07g167700.1.p | SMALL HEAT-SHOCK PROTEIN HSP20 FAMILY // 22.0 KDA HEAT SHOCK PROTEIN | *Arabidopsis thaliana* | 10 | 9 | 56.295 | 0.016 | 14.554 |
| Up | Vigun05g111600.2.p | Universal stress protein family (Usp) | *Arabidopsis thaliana* | 3 | 3 | 20.243 | 0.006 | 3.541 |
| Up | Vigun01g056100.1.p | aldehyde dehydrogenase 5F1 | *Arabidopsis thaliana* | 9 | 9 | 44.810 | 0.001 | 2.660 |
| Down | Vigun11g087500.1.p | multiprotein bridging factor 1B | *Arabidopsis thaliana* | 9 | 9 | 46.848 | 0.008 | 4.867 |
| Down | Vigun01g191000.2.p | TELOMERASE-BINDING PROTEIN P23 HSP90 CO-CHAPERONE | *Arabidopsis thaliana* | 10 | 10 | 66.471 | 0.005 | 2.237 |
| Down | Vigun01g237400.1.p | MOLECULAR CHAPERONE DNAJ | *Vigna unguiculata* | 9 | 5 | 52.848 | 0.002 | 2.977 |
| Down | Vigun01g150500.1.p | heat shock cognate protein 70-1 | *Arabidopsis thaliana* | 40 | 6 | 299.667 | 0.002 | 3.004 |
| Down | Vigun04g144500.1.p | suppressor of G2 allele of SKP1 (SUGT1, SGT1) | *Arabidopsis thaliana* | 11 | 10 | 39.142 | 0.02 | 2.812 |
|  | **Stress, defense and homeostatic response: oxidative stress response and redox homeostasis** | | | |  |  |  |  |
| Up | Vigun09g130300.1.p | PEROXIDASE 52 | *Vigna unguiculata* | 6 | 6 | 35.382 | 0.013 | 3.84 |
| Up | Vigun06g084600.1.p | Peroxidase / Lactoperoxidase | *Vigna unguiculata* | 3 | 2 | 18.554 | 0.017 | 2.969 |
| Up | Vigun03g411400.2.p | Peroxidase superfamily protein | *Vigna unguiculata* | 4 | 3 | 12.282 | 0.009 | 2.001 |
| Up | Vigun05g243700.2.p | copper/zinc superoxide dismutase 2 | *Vigna unguiculata* | 3 | 2 | 38.778 | 0.004 | 3.845 |
| Up | Vigun09g194000.1.p | Tocopherol cyclase | *Arabidopsis thaliana* | 3 | 3 | 17.934 | 0.001 | 3.620 |
| Up | Vigun08g196600.1.p | chloroplastic lipocalin | *Arabidopsis thaliana* | 6 | 5 | 15.342 | 0.002 | 2.233 |
| Up | Vigun10g178300.1.p | ACID PHOSPHATASE-RELATED | *Arabidopsis thaliana* | 17 | 15 | 108.543 | 0.013 | 16.902 |
| Up | Vigun07g213900.1.p | Peroxidase / Lactoperoxidase | *Vigna unguiculata* | 9 | 9 | 50.381 | 0.035 | 2.360 |
| Up | Vigun02g094800.1.p | NADH-UBIQUINONE OXIDOREDUCTASE 39 KDA SUBUNIT-RELATED | *Arabidopsis thaliana* | 13 | 13 | 90.217 | 0.001 | 2.917 |
| Up | Vigun02g117600.2.p | Thiosulfate sulfurtransferase / Thiosulfate thiotransferase | *Arabidopsis thaliana* | 7 | 6 | 57.807 | 0.039 | 2.354 |
| Up | Vigun05g150800.1.p | GLUTAREDOXIN | *Vigna unguiculata* | 9 | 8 | 119.417 | 0.023 | 2.081 |
| Up | Vigun06g212500.1.p | THIOREDOXIN // THIOREDOXIN-LIKE PROTEIN CXXS1 | *Vigna unguiculata* | 3 | 2 | 10.7342 | 0.037 | 2.822 |
| Up | Vigun07g049000.3.p | WCRKC thioredoxin 2 | *Vigna unguiculata* | 5 | 5 | 14.831 | 0.003 | 2.757 |
| Up | Vigun03g236100.1.p | nucleoredoxin [EC:1.8.1.8] (NXN) | *Arabidopsis thaliana* | 7 | 7 | 27.035 | 0.016 | 2.212 |
| Down | Vigun01g027500.1.p | Thioredoxin z | *Vigna unguiculata* | 4 | 4 | 25.051 | 0.010 | 2.186 |
| Down | Vigun05g248900.1.p | Thioredoxin (Thioredoxin) // Thioredoxin-like domain (Thioredoxin_6) // Thioredoxin-like (Thioredoxin_7) | *Vigna unguiculata* | 8 | 8 | 30.505 | 0.025 | 2.198 |
| Down | Vigun02g126300.1.p | thioredoxin H-type 1 | *Vigna unguiculata* | 3 | 3 | 9.750 | 0.002 | 2.046 |
|  | **Stress, defense and homeostatic response: nutrient stress response and homeostasis** | | | |  |  |  |  |
| Up | Vigun11g209200.2.p | mitochondrion-localized small heat shock protein 23.6 | *Arabidopsis thaliana* | 7 | 7 | 41.832 | 0.004 | 9.908 |
| Up | Vigun02g057700.1.p | P30 DBC PROTEIN // PROTEIN LST-3, ISOFORM A | *Vigna unguiculata* | 6 | 4 | 14.347 | 0.023 | 15.108 |
| Up | Vigun06g202700.3.p | allantoinase | *Vigna unguiculata* | 9 | 8 | 94.117 | 0.007 | 2.059 |
| Up | Vigun05g096200.1.p | ferretin 1 | *Vigna unguiculata* | 6 | 3 | 21.627 | 0.001 | 4.692 |
| Up | Vigun08g123500.1.p | ferritin 4 | *Vigna unguiculata* | 8 | 5 | 62.349 | 0.002 | 2.769 |
| Up | Vigun10g086100.1.p | ferretin 1 | *Vigna unguiculata* | 8 | 7 | 52.274 | 0.020 | 2.698 |
| Up | Vigun04g023800.1.p | zinc finger protein-like protein (K16276, BTS) | *Arabidopsis thaliana* | 2 | 2 | 3.597 | 0.006 | 2.529 |
| Up | Vigun11g217500.1.p | purple acid phosphatase 10 | *Arabidopsis thaliana* | 15 | 14 | 136.576 | 0.005 | 2.696 |
| Down | Vigun09g273700.1.p | Zinc knuckle (zf-CCHC) // 'Cold-shock' DNA-binding domain (CSD) | *Vigna unguiculata* | 4 | 3 | 44.161 | 0.002 | 3.733 |
| Down | Vigun07g133300.1.p | DEAD box RNA helicase (RH3) | *Arabidopsis thaliana* | 18 | 17 | 111.026 | 0.004 | 2.896 |
|  | **Stress, defense and homeostatic response: water deprivation stress response** | | |  |  |  |  |  |
| Up | Vigun06g022400.1.p | Late embryogenesis abundant protein, group 2 | *Vigna unguiculata* | 8 | 6 | 57.342 | 0.002 | 5.4131 |
| Up | Vigun04g169000.1.p | plastid movement impaired1 | *Arabidopsis thaliana* | 6 | 5 | 31.406 | 0.036 | 2.425 |
| Up | Vigun09g017100.1.p | DREPP plasma membrane polypeptide (DREPP) | *Arabidopsis thaliana* | 7 | 6 | 57.018 | 0.003 | 2.794 |
| Up | Vigun11g018900.1.p | cystatin B | *Arabidopsis thaliana* | 4 | 4 | 29.72 | 0.024 | 2.030 |
| Down | Vigun05g048200.1.p | plasminogen activator inhibitor 1 RNA-binding protein (SERBP1) | *Arabidopsis thaliana* | 9 | 9 | 64.898 | 0.01 | 5.133 |
| Down | Vigun07g294500.1.p | plasminogen activator inhibitor 1 RNA-binding protein (SERBP1) | *Arabidopsis thaliana* | 9 | 8 | 81.016 | 0.005 | 6.187 |
| Down | Vigun10g009600.1.p | plasminogen activator inhibitor 1 RNA-binding protein (SERBP1) | *Arabidopsis thaliana* | 13 | 10 | 95.505 | 0.042 | 2.844 |
|  | **Stress, defense and homeostatic response: other stress responses** | | |  |  |  |  |  |
| Up | Vigun04g130500.1.p | ABA/WDS induced protein (ABA_WDS) | *Vigna unguiculata* | 8 | 8 | 106.123 | 0.006 | 7.257 |
| Up | Vigun04g194000.1.p | Adenine nucleotide alpha hydrolases-like superfamily protein | *Vigna unguiculata* | 2 | 2 | 13.412 | 0.007 | 5.790 |
| Up | Vigun10g164800.1.p | GERMIN-LIKE PROTEIN SUBFAMILY 3 MEMBER 3 | *Arabidopsis thaliana* | 5 | 5 | 131.088 | 0.030 | 4.221 |
| Up | Vigun07g287200.1.p | dehydroascorbate reductase 2 | *Arabidopsis thaliana* | 4 | 3 | 19.138 | 0.01 | 3.565 |
| Up | Vigun01g236000.1.p | Lipase/lipooxygenase, PLAT/LH2 family protein | *Vigna unguiculata* | 3 | 3 | 19.810 | 0.002 | 3.991 |
| Up | Vigun06g157000.1.p | Plastid-lipid associated protein PAP / fibrillin family protein | *Arabidopsis thaliana* | 15 | 14 | 156.386 | 0.010 | 5.152 |
| Down | Vigun05g222200.1.p | Universal stress protein family (Usp) | *Vigna unguiculata* | 4 | 4 | 19.659 | 0.013 | 2.312 |
| Down | Vigun07g023900.1.p | HEAT SHOCK PROTEIN 90 HSP90 CO-CHAPERONE AHA-1 // AHSA1 PROTEIN | *Vigna unguiculata* | 2 | 2 | 5.777 | 0.006 | 2.716 |
|  | **Transcription** |  |  |  |  |  |  |  |
| Up | VigunL078600.1.p | heat shock protein 90.1 | *Vigna unguiculata* | 27 | 15 | 203.280 | 0.009 | 4.252 |
| Down | Vigun07g057500.1.p | Splicing factor 3b, subunit 4 | *Arabidopsis thaliana* | 7 | 5 | 45.545 | 0.007 | 2.039 |
| Down | Vigun02g009300.5.p | U2 small nuclear ribonucleoprotein A | *Arabidopsis thaliana* | 8 | 8 | 42.457 | 0.003 | 2.643 |
| Down | Vigun09g226300.1.p | KH domain-containing protein | *Arabidopsis thaliana* | 19 | 3 | 127.509 | 0.005 | 9.205 |
| Down | Vigun09g226200.2.p | far upstream element-binding protein (FUBP) | *Arabidopsis thaliana* | 18 | 2 | 126.259 | 0.011 | 2.886 |
| Down | Vigun02g186400.2.p | far upstream element-binding protein (FUBP) | *Arabidopsis thaliana* | 4 | 4 | 28.152 | 0.004 | 3.008 |
| Down | Vigun02g047300.1.p | fructokinase-like 2 | *Arabidopsis thaliana* | 2 | 2 | 6.82 | 0.014 | 5.557 |
| Down | Vigun10g069600.1.p | fructokinase-like 1 | *Arabidopsis thaliana* | 2 | 2 | 4.717 | 0.019 | 2.276 |
| Down | Vigun01g060500.1.p | ribosome biogenesis protein UTP30 (UTP30, RSL1D1) | *Arabidopsis thaliana* | 2 | 2 | 5.644 | 0.004 | 11.464 |
| Down | Vigun01g252100.1.p | S-adenosyl-L-homocysteine hydrolase | *Arabidopsis thaliana* | 24 | 22 | 159.498 | 0.008 | 2.062 |
| Down | Vigun11g175800.1.p | cold, circadian rhythm, and rna binding 2 | *Arabidopsis thaliana* | 14 | 12 | 109.068 | 0.006 | 2.917 |
| Down | Vigun05g276200.4.p | Uncharacterized protein | *Vigna unguiculata* | 2 | 2 | 5.4982 | 0.015 | 2.334 |
| Down | Vigun05g040300.4.p | RNA-binding KH domain-containing protein | *Arabidopsis thaliana* | 3 | 3 | 6.187 | 0.030 | 2.44 |
| Down | Vigun03g135500.1.p | heterogeneous nuclear ribonucleoprotein G (RBMX, HNRNPG) | *Arabidopsis thaliana* | 2 | 2 | 11.677 | 0.009 | 2.138 |
| Down | Vigun03g387800.1.p | ZINC FINGER CCCH DOMAIN-CONTAINING PROTEIN 40-RELATED | *Arabidopsis thaliana* | 5 | 5 | 28.920 | 0.003 | 2.638 |
| Down | Vigun01g181000.1.p | chloroplast RNA-binding protein 33 | *Arabidopsis thaliana* | 11 | 9 | 65.610 | 0.003 | 2.046 |
| Down | Vigun01g007100.1.p | ankyrin repeat-containing 2B | *Arabidopsis thaliana* | 19 | 17 | 114.259 | 0.006 | 2.425 |
| Down | Vigun03g212100.1.p | ssDNA-binding transcriptional regulator | *Vigna unguiculata* | 2 | 2 | 11.74 | 0.004 | 2.065 |
| Down | Vigun11g189200.2.p | cold shock domain protein 1 | *Vigna unguiculata* | 4 | 3 | 42.357 | 0.002 | 3.158 |
| Down | Vigun03g199900.1.p | N UTILIZATION SUBSTANCE PROTEIN B-RELATED // ANTITERMINATION NUSB DOMAIN-CONTAINING PROTEIN | *Vigna unguiculata* | 3 | 3 | 17.284 | 0.006 | 5.194 |
| Down | Vigun07g100900.2.p | Rho termination factor | *Vigna unguiculata* | 10 | 9 | 61.773 | 0.015 | 2.579 |
| Down | Vigun06g180900.1.p | plastid transcriptionally active 12 | *Vigna unguiculata* | 3 | 3 | 19.199 | 0.006 | 8.265 |
| Down | Vigun05g186800.1.p | cobalt ion binding | *Vigna unguiculata* | 5 | 3 | 23.709 | 0.002 | 3.179 |
| Down | Vigun07g118200.2.p | U6 snRNA-associated Sm-like protein LSm4 (LSM4) | *Vigna unguiculata* | 2 | 2 | 18.181 | 0.026 | 2.336 |
| Down | Vigun03g008800.1.p | THO2 | *Arabidopsis thaliana* | 3 | 3 | 7.222 | 0.019 | 3.993 |
|  | **Vitamin metabolism** | |  |  |  |  |  |  |
| Up | Vigun02g099400.1.p | Dihydroneopterin aldolase | *Vigna unguiculata* | 2 | 2 | 21.721 | 0.046 | 2.042 |
| Down | Vigun10g078700.1.p | pyridoxine biosynthesis 2 | *Vigna unguiculata* | 8 | 8 | 51.724 | 0.003 | 2.621 |
| Down | Vigun06g185700.1.p | pyridoxal 5'-phosphate synthase pdxS subunit (pdxS, pdx1) | *Vigna unguiculata* | 16 | 15 | 79.821 | 0.004 | 2.225 |
|  | **Other metabolisms** | |  |  |  |  |  |  |
| Up | Vigun10g075700.1.p | Uncharacterized protein | *Arabidopsis thaliana* | 2 | 2 | 7.383 | 0.015 | 2.628 |
| Up | Vigun10g009900.1.p | uclacyanin 3 | *Arabidopsis thaliana* | 2 | 2 | 2.868 | 0.011 | 2.339 |
| Down | Vigun03g413500.1.p | DNA-binding enhancer protein-related | *Vigna unguiculata* | 3 | 3 | 12.532 | 0.003 | 12.396 |
| Down | Vigun02g194800.1.p | 2Fe-2S ferredoxin-like superfamily protein | *Vigna unguiculata* | 4 | 4 | 41.865 | 0.004 | 6.324 |
| Down | Vigun04g056500.1.p | LAMIN-LIKE PROTEIN | *Vigna unguiculata* | 2 | 2 | 2.92 | 0.002 | 2.33 |
| Down | Vigun03g152100.5.p | 23S rRNA (adenine(1618)-N(6))-methyltransferase / rRNA large subunit methyltransferase F | *Arabidopsis thaliana* | 11 | 10 | 68.195 | 0.018 | 2.428 |
| Down | Vigun09g175800.1.p | Uncharacterized protein | *Arabidopsis thaliana* | 2 | 2 | 9.281 | 0.019 | 3.225 |
| Up | Vigun09g053000.1.p | S-adenosyl-L-methionine-dependent methyltransferases superfamily protein | *Arabidopsis thaliana* | 3 | 2 | 3.973 | 0.011 | 2.264 |
| Up | Vigun05g112100.1.p | glutathione S-transferase TAU 8 | *Vigna unguiculata* | 2 | 2 | 11.543 | 0.036 | 2.042 |
| Up | Vigun03g313300.2.p | purple acid phosphatase 27 | *Arabidopsis thaliana* | 4 | 4 | 24.909 | 0.001 | 8.257 |
| Up | Vigun11g130800.1.p | 2-carboxy-D-arabinitol-1-phosphatase | *Arabidopsis thaliana* | 4 | 4 | 19.842 | 0.014 | 2.041 |
| Down | Vigun06g143800.2.p | APOPTOSIS INHIBITOR 5-RELATED // PEPTIDASE-C1 DOMAIN-CONTAINING PROTEIN | *Arabidopsis thaliana* | 4 | 3 | 14.668 | 0.008 | 13.120 |
| Down | Vigun02g158100.3.p | 2-DEOXYGLUCOSE-6-PHOSPHATE PHOSPHATASE 2 // HALOACID DEHALOGENASE-LIKE HYDROLASE DOMAIN-CONTAINING PROTEIN SGPP | *Arabidopsis thaliana* | 13 | 11 | 49.640 | 0.001 | 4.146 |
| Down | Vigun06g196000.1.p | Phosphoprotein/predicted coiled-coil protein | *Vigna unguiculata* | 4 | 4 | 19.630 | 0.012 | 4.126 |
| Down | Vigun03g174300.1.p | Apoptosis inhibitory protein 5 (API5) | *Arabidopsis thaliana* | 2 | 2 | 3.022 | 0.016 | 2.937 |
| Down | Vigun03g225100.1.p | ADP-ribosylation factor GTPase-activating protein 2/3 (ARFGAP2_3) | *Vigna unguiculata* | 4 | 4 | 19.899 | 0.010 | 2.827 |
| Up | Vigun03g066400.3.p | Plastid-lipid associated protein PAP / PAP_fibrillin | *Arabidopsis thaliana* | 3 | 2 | 9.377 | 0.003 | 2.357 |
| Down | Vigun05g018000.1.p | 4-sulfomuconolactone hydrolase | *Arabidopsis thaliana* | 2 | 2 | 11.121 | 0.009 | 3.135 |
| Down | Vigun01g219500.1.p | NAD(P)-binding Rossmann-fold superfamily protein | *Vigna unguiculata* | 7 | 7 | 48.980 | 0.015 | 2.459 |
| Down | Vigun09g240300.1.p | Uroporphyrinogen-III synthase / Uroporphyrinogen-III cosynthetase | *Vigna unguiculata* | 3 | 3 | 22.084 | 0.035 | 2.387 |
|  | **Function unknown** | |  |  |  |  |  |  |
| Up | Vigun07g200200.1.p | Arsenite-transporting ATPase / Arsenite-translocating ATPase | *Vigna unguiculata* | 2 | 2 | 6.231 | 0.036 | 2.264 |
| Down | Vigun05g300200.2.p | EKC/KEOPS complex subunit CGI121/TPRKB (CGI121, TPRKB) | *Vigna unguiculata* | 2 | 2 | 15.947 | 0.002 | 2.555 |
| Down | Vigun02g091800.1.p | RNA-binding (RRM/RBD/RNP motifs) family protein | *Vigna unguiculata* | 4 | 3 | 22.465 | 0.002 | 2.17 |
| Down | Vigun01g034200.1.p | basic transcription factor 3 | *Vigna unguiculata* | 8 | 8 | 77.111 | 0.002 | 2.819 |
| Down | Vigun09g266600.1.p | small subunit ribosomal protein S25e (RP-S25e, RPS25) | *Vigna unguiculata* | 8 | 8 | 61.215 | 0.002 | 2.383 |
| Up | Vigun11g202900.1.p | NAD DEPENDENT EPIMERASE/DEHYDRATASE // ALCOHOL DEHYDROGENASE-RELATED | *Vigna unguiculata* | 28 | 6 | 306.046 | 0.034 | 2.102 |
| Up | Vigun01g037600.1.p | Cinnamyl-alcohol dehydrogenase / CAD | *Vigna unguiculata* | 17 | 5 | 128.507 | 0.013 | 9.642 |
| Up | Vigun04g037800.3.p | Methylecgonone reductase | *Vigna unguiculata* | 3 | 3 | 7.517 | 0.015 | 2.841 |
| Up | Vigun06g080600.1.p | interferon, gamma-inducible protein 30 (IFI30, GILT) | *Vigna unguiculata* | 3 | 3 | 8.232 | 0.016 | 2.146 |
| Up | Vigun06g207000.1.p | QUINONE OXIDOREDUCTASE PIG3 | *Vigna unguiculata* | 3 | 3 | 5.070 | 0.04 | 2.029 |
| Up | Vigun07g224000.2.p | NAD(P)-binding Rossmann-fold superfamily protein | *Vigna unguiculata* | 11 | 11 | 55.675 | 0.003 | 2.333 |
| Up | Vigun07g262500.2.p | FAD/NAD(P)-binding oxidoreductase | *Vigna unguiculata* | 15 | 15 | 162.631 | 0.009 | 2.507 |
| Up | Vigun03g118200.1.p | Uncharacterized protein | *Vigna unguiculata* | 5 | 2 | 36.585 | 0.004 | 21.486 |
| Up | Vigun10g050400.1.p | ALDO-KETO REDUCTASE FAMILY 4 MEMBER C10 | *Vigna unguiculata* | 18 | 7 | 100.988 | 0.006 | 3.453 |
| Up | Vigun10g050700.2.p | ALDO-KETO REDUCTASE FAMILY 4 MEMBER C10 | *Vigna unguiculata* | 14 | 4 | 68.570 | 0.044 | 2.815 |
| Up | Vigun10g104500.1.p | CYTOCHROME P450 71B21-RELATED | *Vigna unguiculata* | 6 | 6 | 32.065 | 0.014 | 2.251 |
| Down | Vigun05g185700.1.p | general regulatory factor 9 | *Vigna unguiculata* | 4 | 2 | 10.461 | 0.004 | 3.098 |
| Up | Vigun11g224200.1.p | ALCOHOL DEHYDROGENASE RELATED // QUINONE-OXIDOREDUCTASE HOMOLOG, CHLOROPLASTIC-RELATED | *Vigna unguiculata* | 3 | 2 | 28.464 | 0.002 | 4.244 |
| Up | Vigun10g123400.2.p | LEUCINE-RICH REPEAT-CONTAINING PROTEIN | *Vigna unguiculata* | 17 | 17 | 193.953 | 0.005 | 2.538 |
| Down | Vigun01g236600.1.p | DNA oxidative demethylase / Alkylated DNA repair protein | *Vigna unguiculata* | 2 | 2 | 11.4253 | 0.016 | 264.789 |
| Down | Vigun07g096900.1.p | RNA POLYMERASE SIGMA FACTOR RPO | *Vigna unguiculata* | 2 | 2 | 3.052 | 0.027 | 6.236 |
| Down | Vigun10g145000.1.p | RNA HELICASE // DEAD-BOX ATP-DEPENDENT RNA HELICASE 25-RELATED | *Vigna unguiculata* | 5 | 5 | 17.17 | 0.013 | 2.524 |
| Down | Vigun11g214700.1.p | Nucleic acid-binding, OB-fold-like protein | *Vigna unguiculata* | 4 | 4 | 19.681 | 0.002 | 2.159 |
| Down | Vigun06g081000.1.p | RNA-binding protein FUS (TLS, FUS) | *Vigna unguiculata* | 2 | 2 | 6.349 | 0.013 | 2.001 |
| Up | Vigun09g162400.1.p | DPP6 N-terminal domain-like protein | *Vigna unguiculata* | 20 | 19 | 179.154 | 0.003 | 2.181 |
| Down | Vigun09g133400.1.p | aminoacyl tRNA synthase complex-interacting multifunctional protein 1 (AIMP1, ARC1) | *Vigna unguiculata* | 3 | 3 | 10.956 | 0.003 | 2.171 |
| Down | Vigun06g006300.1.p | RNA-binding (RRM/RBD/RNP motifs) family protein | *Vigna unguiculata* | 3 | 2 | 16.534 | 0.011 | 2.061 |
| Down | Vigun10g015200.2.p | RNA-binding (RRM/RBD/RNP motifs) family protein | *Vigna unguiculata* | 5 | 5 | 12.776 | 0.016 | 2.275 |
| Down | Vigun11g180100.1.p | RNA-binding protein (yhbY) | *Vigna unguiculata* | 5 | 3 | 28.509 | 0.005 | 13.243 |
| Down | Vigun10g191900.4.p | TETRATRICOPEPTIDE REPEAT-CONTAINING PROTEIN | *Vigna unguiculata* | 6 | 2 | 9.478 | 0.027 | 4.996 |
| Up | Vigun03g164800.1.p | amidase (E3.5.1.4, amiE) | *Vigna unguiculata* | 3 | 3 | 4.936 | 0.044 | 2.026 |
| Down | Vigun09g207200.3.p | lysophospholipase II (LYPLA2) | *Vigna unguiculata* | 4 | 4 | 12.413 | 0.006 | 2.067 |
| Down | Vigun09g259500.1.p | Uncharacterized protein | *Vigna unguiculata* | 6 | 5 | 14.59 | 0.001 | 5.366 |
| Down | Vigun09g262000.1.p | CHLOROPLAST INNER MEMBRANE LOCALIZED PROTEIN | *Vigna unguiculata* | 2 | 2 | 12.347 | 0.022 | 2.488 |
| Down | Vigun07g164100.1.p | Protein O-GlcNAc transferase / OGTase | *Vigna unguiculata* | 2 | 2 | 10.396 | 0.031 | 3.123 |
| Down | Vigun07g186100.1.p | GLUCOSYL/GLUCURONOSYL TRANSFERASES | *Vigna unguiculata* | 4 | 4 | 20.402 | 0.003 | 3.399 |
| Down | Vigun08g063100.1.p | SELENOPROTEIN H | *Vigna unguiculata* | 2 | 2 | 13.582 | 0.005 | 3.842 |
| Down | Vigun05g078600.1.p | FASCICLIN-like arabinogalactan-protein 10 | *Vigna unguiculata* | 8 | 8 | 39.278 | 0.002 | 3.440 |
| Down | Vigun06g177200.1.p | Protein of unknown function, DUF642 | *Vigna unguiculata* | 2 | 2 | 6.398 | 0.009 | 3.037 |
| Down | Vigun07g052600.1.p | AMMECR1 family protein | *Vigna unguiculata* | 2 | 2 | 4.613 | 0.011 | 4.0916 |
| Down | Vigun03g277100.3.p | Uncharacterized protein | *Vigna unguiculata* | 3 | 3 | 8.24 | 0.018 | 4.264 |
| Down | Vigun03g349600.1.p | Uncharacterized protein | *Vigna unguiculata* | 3 | 2 | 17.876 | 0.03 | 13.771 |
| Down | Vigun04g440400.1.p | Uncharacterized protein | *Vigna unguiculata* | 3 | 3 | 12.060 | 0.003 | 2.376 |
| Down | Vigun04g061000.1.p | Uncharacterized protein | *Vigna unguiculata* | 6 | 5 | 37.679 | 0.027 | 2.001 |
| Up | Vigun10g126600.1.p | Remorin, C-terminal region (Remorin_C) // Remorin, N-terminal region (Remorin_N) | *Vigna unguiculata* | 4 | 2 | 13.074 | 0.035 | 2.857 |
| Up | Vigun10g137000.2.p | Protein of unknown function (DUF674) | *Vigna unguiculata* | 2 | 2 | 3.277 | 0.005 | 3.309 |
| Up | Vigun10g193100.2.p | CIRCADIAN PROTEIN CLOCK/ARNT/BMAL/PAS | *Vigna unguiculata* | 13 | 6 | 136.422 | 0.018 | 2.196 |
| Up | Vigun10g198600.4.p | Plastid-lipid associated protein PAP / fPAP_fibrillin | *Vigna unguiculata* | 2 | 2 | 16.254 | 0.002 | 3.92 |
| Up | Vigun07g292300.1.p | Plastid-lipid associated protein PAP / PAP_fibrillin | *Vigna unguiculata* | 4 | 4 | 22.396 | 0.004 | 2.36 |
| Up | Vigun08g220600.1.p | Uncharacterized protein | *Vigna unguiculata* | 3 | 3 | 20.852 | 0.002 | 2.236 |
| Up | Vigun09g145200.1.p | Rhodanese/Cell cycle control phosphatase superfamily protein | *Vigna unguiculata* | 5 | 5 | 55.195 | 0.029 | 2.339 |
| Up | Vigun05g055000.1.p | Uncharacterized conserved protein (DUF2358) | *Vigna unguiculata* | 2 | 2 | 6.065 | 0.002 | 3.387 |
| Up | Vigun05g093700.1.p | NUCLEOPORIN-RELATED // DIRIGENT PROTEIN 19 | *Vigna unguiculata* | 3 | 2 | 10.147 | 0.019 | 2.247 |
| Up | Vigun07g005600.1.p | Hemopexin (Hemopexin) | *Vigna unguiculata* | 4 | 2 | 12.828 | 0.015 | 6.997 |
| Up | Vigun07g025500.1.p | Plastid-lipid associated protein PAP / PAP_fibrillin | *Vigna unguiculata* | 12 | 12 | 64.268 | 0.002 | 2.107 |
| Up | Vigun07g146900.1.p | Domain of unknown function (DUF1995) | *Vigna unguiculata* | 4 | 4 | 19.694 | 0.018 | 9.170 |
| Up | Vigun07g159900.1.p | Uncharacterized protein | *Vigna unguiculata* | 2 | 2 | 12.345 | 0.01 | 3.3157 |
| Up | Vigun03g355500.2.p | Protein of unknown function (DUF3119) (DUF3119) | *Vigna unguiculata* | 2 | 2 | 17.950 | 0.002 | 2.689 |
| Up | Vigun04g030400.1.p | Calcium-binding EF-hand family protein | *Vigna unguiculata* | 2 | 2 | 7.838 | 0.01 | 2.534 |
| Up | Vigun03g253300.1.p | Uncharacterized protein | *Vigna unguiculata* | 4 | 4 | 18.6 | 0.001 | 3.348 |
| Up | Vigun10g191800.1.p | alpha/beta-Hydrolases superfamily protein | *Vigna unguiculata* | 5 | 5 | 33.865 | 0.001 | 2.382 |
| Down | Vigun05g140600.1.p | Lactoylglutathione lyase / glyoxalase I family protein | *Vigna unguiculata* | 3 | 3 | 31.752 | 0.007 | 4.079 |
| Up | Vigun10g053000.1.p | ATP-DEPENDENT CLP PROTEASE | *Vigna unguiculata* | 39 | 14 | 213.105 | 0.003 | 6.596 |
| Up | Vigun07g296000.1.p | terpene synthase 03 | *Vigna unguiculata* | 2 | 2 | 4.9216 | 0.004 | 2.801 |
| Up | Vigun01g243900.1.p | heat shock protein 21 | *Vigna unguiculata* | 13 | 12 | 62.892 | 0.002 | 20.834 |
| Up | Vigun06g052200.1.p | heat shock protein 21 | *Vigna unguiculata* | 20 | 20 | 158.351 | 0.003 | 29.459 |
| Down | Vigun07g227900.1.p | MEMBRANE-ASSOCIATED PROGESTERONE RECEPTOR COMPONENT-RELATED // MEMBRANE STEROID-BINDING PROTEIN 1-RELATED | *Vigna unguiculata* | 8 | 7 | 65.294 | 0.014 | 3.291 |
| Down | Vigun01g234400.1.p | apoptotic chromatin condensation inducer in the nucleus (ACIN1, ACINUS) | *Vigna unguiculata* | 7 | 7 | 33.838 | 0.011 | 2.722 |
| Down | Vigun02g202800.1.p | SWIB/MDM2 domain superfamily protein | *Vigna unguiculata* | 5 | 3 | 15.622 | 0.01 | 4.475 |
| Up | Vigun03g255200.2.p | Uncharacterized protein | *Vigna unguiculata* | 4 | 4 | 8.010 | 0.002 | 2.895 |
| Down | Vigun11g040500.1.p | GTP-BINDING PROTEIN-RELATED | *Vigna unguiculata* | 6 | 6 | 34.859 | 0.018 | 2.159 |
| Up | Vigun07g048400.1.p | Concanavalin A-like lectin protein kinase family protein | *Vigna unguiculata* | 17 | 14 | 179.854 | 0.034 | 2.7 |
| Up | Vigun07g226600.1.p | Concanavalin A-like lectin protein kinase family protein | *Vigna unguiculata* | 6 | 4 | 38.455 | 0.019 | 3.183 |
| Down | Vigun06g012000.2.p | NUCLEAR INHIBITOR OF PROTEIN PHOSPHATASE-1 | *Vigna unguiculata* | 9 | 8 | 108.626 | 0.004 | 2.622 |
| Up | Vigun08g182100.1.p | cystatin B | *Vigna unguiculata* | 11 | 9 | 114.531 | 0.001 | 12.311 |
| Down | Vigun08g003400.1.p | RIBONUCLEASE P SUBUNIT P25 | *Vigna unguiculata* | 4 | 4 | 26.606 | 0.008 | 4.545 |
| Up | Vigun09g256300.1.p | Thioredoxin (Thioredoxin_4) | *Vigna unguiculata* | 7 | 7 | 52.755 | 0.013 | 3.366 |
| Down | Vigun08g211700.1.p | APOPTOSIS INHIBITOR 5-RELATED // GBF-INTERACTING PROTEIN 1 | *Vigna unguiculata* | 3 | 3 | 12.952 | 0.024 | 5.891 |
| Down | Vigun09g169800.1.p | PPR repeat (PPR) // Ubiquitin fold modifier 1 protein (Ufm1) // PPR repeat family (PPR_2) | *Vigna unguiculata* | 3 | 3 | 14.548 | 0.012 | 3.497 |
| Up | Vigun09g221300.1.p | Chaperone DnaJ-domain superfamily protein | *Vigna unguiculata* | 6 | 6 | 39.745 | 0.001 | 3.193 |
| Up | Vigun02g094000.1.p | PLASTID-LIPID-ASSOCIATED PROTEIN 3, CHLOROPLASTIC-RELATED | *Vigna unguiculata* | 11 | 10 | 103.969 | 0.002 | 2.204 |
| Up | Vigun01g253400.2.p | Rhodanese/Cell cycle control phosphatase superfamily protein | *Vigna unguiculata* | 15 | 13 | 85.013 | 0.004 | 2.268 |
| Up | Vigun03g148600.1.p | Predicted dehydrogenase | *Vigna unguiculata* | 7 | 7 | 43.550 | 0.002 | 2.077 |
