# Supplementary material for: Regulation and physiological function of proteins for heat tolerance in cowpea (Vigna unguiculata) genotypes under controlled and field conditions
Source: Front Plant Sci. 2022 Aug 22;13:954527. doi: 10.3389/fpls.2022.954527 (PMC9441852; doi:10.3389/fpls.2022.954527)
Supplement: Supplementary file 3 [file Table_3.DOCX]

Table 3: List of responsive proteins regulated at Marapyane compared to at Eensaamheid from IT-16 using the label free quantification and database searches.

| **Regulation** | **Accession** | **Protein name** | **Species name from which protein or orthologue was obtained** | **Peptide count** | **Unique peptides** | **Confidence score** | **q Value** | **Max fold change** |
| --- | --- | --- | --- | --- | --- | --- | --- | --- |
|  | **Amino acid metabolism** | |  |  |  |  |  |  |
| Down | Vigun02g156500.1.p | glycine decarboxylase complex H | *Vigna unguiculata* | 9 | 7 | 161.772 | 0.017 | 3.197 |
| Down | Vigun04g056600.1.p | chorismate mutase 1 | *Vigna unguiculata* | 3 | 3 | 14.041 | 0.014 | 2.635 |
| Down | Vigun05g063500.3.p | cysteine synthase C1 | *Vigna unguiculata* | 12 | 10 | 78.116 | 0.006 | 2.981 |
| Down | Vigun06g236000.1.p | ATP phosphoribosyl transferase 2 | *Vigna unguiculata* | 3 | 3 | 5.231 | 0.011 | 2.196 |
| Down | Vigun07g071800.1.p | methionine adenosyltransferase 3 | *Vigna unguiculata* | 11 | 4 | 58.087 | 0.011 | 3.933 |
| Down | Vigun11g078900.1.p | thiamine thiazole synthase (THI4, THI1) | *Arabidopsis thaliana* | 11 | 9 | 56.483 | 0.008 | 2.702 |
|  | **Carbohydrate and energy metabolism** | |  |  |  |  |  |  |
| Up | Vigun08g057100.1.p | Protein of unknown function, DUF642 | *Arabidopsis thaliana* | 3 | 2 | 14.494 | 0.023 | 3.722 |
| Up | Vigun01g079000.2.p | ALPHA-MANNOSIDASE | *Vigna unguiculata* | 6 | 3 | 16.436 | 0.046 | 8.024 |
| Up | Vigun05g084800.2.p | alpha-galactosidase 1 | *Vigna unguiculata* | 5 | 4 | 37.394 | 0.010 | 3.828 |
| Up | Vigun07g252100.1.p | PERIPLASMIC BETA-GLUCOSIDASE-RELATED | *Vigna unguiculata* | 4 | 3 | 22.03 | 0.003 | 8.849 |
| Up | Vigun11g046000.1.p | chitinase A | *Vigna unguiculata* | 2 | 2 | 10.949 | 0.035 | 3.654 |
| Up | Vigun11g072700.5.p | beta glucosidase 41 | *Vigna unguiculata* | 10 | 10 | 78.536 | 0.009 | 2.437 |
| Up | Vigun03g379900.1.p | Xylose isomerase / D-xylose ketoisomerase | *Vigna unguiculata* | 6 | 6 | 16.925 | 0.031 | 2.178 |
| Up | Vigun10g151700.1.p | NDP-glucose--starch glucosyltransferase / Waxy protein | *Vigna unguiculata* | 6 | 6 | 42.206 | 0.01 | 2.115 |
| Up | Vigun11g166500.1.p | pfkB-like carbohydrate kinase family protein | *Vigna unguiculata* | 9 | 5 | 59.271 | 0.008 | 4.660 |
| Down | Vigun07g075700.1.p | isocitrate dehydrogenase V | *Vigna unguiculata* | 6 | 6 | 28.398 | 0.003 | 2.805 |
| Down | Vigun08g200600.1.p | Dihydrolipoamide succinyltransferase | *Vigna unguiculata* | 9 | 9 | 58.618 | 0.047 | 2.09 |
| Down | Vigun06g119900.1.p | chitinase A | *Vigna unguiculata* | 2 | 2 | 12.787 | 0.021 | 2.475 |
| Down | Vigun05g003900.1.p | transaldolase (E2.2.1.2, talA, talB) | *Vigna unguiculata* | 9 | 9 | 48.227 | 0.017 | 2.087 |
| Down | Vigun08g057300.1.p | Cytochrome bd ubiquinol oxidase, 14kDa subunit | *Vigna unguiculata* | 8 | 8 | 61.744 | 0.043 | 2.178 |
| Down | Vigun03g446600.1.p | starch synthase 3 | *Arabidopsis thaliana* | 7 | 6 | 31.238 | 0.044 | 2.073 |
|  | **Cell cycle, defferentiation and development** | |  |  |  |  |  |  |
| Up | Vigun05g282100.1.p | TETRAKETIDE ALPHA-PYRONE REDUCTASE 1 | *Arabidopsis thaliana* | 29 | 5 | 265.039 | 0.008 | 5.704 |
| Up | Vigun08g112900.1.p | CHAPERONE-ACTIVITY OF BC1 COMPLEX CABC1 -RELATED | *Arabidopsis thaliana* | 3 | 3 | 5.014 | 0.035 | 2.601 |
| Down | Vigun06g185000.1.p | Dynamin related protein 4C | *Arabidopsis thaliana* | 8 | 6 | 23.743 | 0.006 | 3.112 |
| Down | Vigun11g155100.1.p | actin 3 | *Arabidopsis thaliana* | 17 | 2 | 123.945 | 0.021 | 2.333 |
| Down | Vigun06g218000.1.p | dynamin-related protein 3A | *Arabidopsis thaliana* | 4 | 2 | 14.843 | 0.012 | 8.204 |
| Down | Vigun08g194200.1.p | STRUCTURAL MAINTENANCE OF CHROMOSOMES SMC FAMILY MEMBER | *Arabidopsis thaliana* | 5 | 4 | 24.674 | 0.006 | 4.131 |
| Down | Vigun03g260100.1.p | B-box type zinc finger family protein | *Arabidopsis thaliana* | 7 | 6 | 40.299 | 0.017 | 2.583 |
| Down | Vigun07g076000.1.p | chloroplast RNA-binding protein 29 | *Arabidopsis thaliana* | 5 | 2 | 54.603 | 0.021 | 2.653 |
| Down | Vigun11g192700.1.p | Nuclear localization sequence binding protein | *Arabidopsis thaliana* | 10 | 10 | 53.538 | 0.009 | 3.424 |
| Down | Vigun11g181800.1.p | Dihydrokaempferol 4-reductase / NADPH-dihydromyricetin reductase // Farnesol dehydrogenase (NAD(+)) / NAD(+)-farnesol dehydrogenase | *Arabidopsis thaliana* | 11 | 9 | 67.878 | 0.040 | 2.31 |
|  | **Cellular component organization** | |  |  |  |  |  |  |
| Up | Vigun02g198300.1.p | methylesterase PCR A | *Vigna unguiculata* | 2 | 2 | 5.573 | 0.012 | 3.054 |
| Up | Vigun10g157000.1.p | pectin methylesterase 3 | *Vigna unguiculata* | 6 | 3 | 32.388 | 0.008 | 2.118 |
| Up | Vigun11g209500.1.p | peroxin 11B | *Vigna unguiculata* | 2 | 2 | 4.0763 | 0.045 | 2.037 |
| Up | Vigun02g198200.1.p | methylesterase PCR A | *Vigna unguiculata* | 4 | 4 | 24.740 | 0.009 | 4.126 |
| Down | Vigun10g071200.1.p | tubulin beta chain 2 | *Vigna unguiculata* | 11 | 11 | 49.734 | 0.034 | 2.059 |
| Down | Vigun09g059800.1.p | reversibly glycosylated polypeptide 3 | *Vigna unguiculata* | 5 | 5 | 19.389 | 0.002 | 3.990 |
| Down | Vigun07g105500.1.p | histone H2A 11 | *Arabidopsis thaliana* | 4 | 3 | 18.643 | 0.038 | 2.752 |
| Down | Vigun03g393100.1.p | nucleosome assembly protein 1;2 | *Vigna unguiculata* | 4 | 2 | 20.471 | 0.016 | 2.402 |
| Down | Vigun05g240500.2.p | histone deacetylase 2B | *Arabidopsis thaliana* | 2 | 2 | 6.650 | 0.022 | 4.281 |
| Down | Vigun09g033900.1.p | nucleosome assembly protein 1;2 | *Vigna unguiculata* | 4 | 3 | 23.533 | 0.006 | 3.722 |
|  | **Lipid metabolism** |  |  |  |  |  |  |  |
| Up | Vigun03g036400.1.p | CDP-diacylglycerol--inositol 3-phosphatidyltransferase / Phosphatidylinositol synthase // Long-chain-fatty-acid--CoA ligase / Lignoceroyl-CoA synthase | *Arabidopsis thaliana* | 6 | 6 | 14.375 | 0.013 | 2.704 |
| Up | Vigun06g183700.1.p | GDSL-like Lipase/Acylhydrolase superfamily protein | *Arabidopsis thaliana* | 10 | 9 | 72.872 | 0.003 | 5.004 |
| Up | Vigun06g217400.1.p | UDP-glucosyl transferase 78D2 | *Medicago truncatula* | 3 | 3 | 20.660 | 0.013 | 2.780 |
| Down | Vigun07g051400.1.p | peroxisomal 3-ketoacyl-CoA thiolase 3 | *Arabidopsis thaliana* | 12 | 12 | 98.234 | 0.030 | 2.239 |
| Down | Vigun05g089100.1.p | 3-ketoacyl-acyl carrier protein synthase III | *Vigna unguiculata* | 2 | 2 | 12.376 | 0.006 | 3.101 |
| Down | Vigun08g205800.1.p | GLYCEROPHOSPHODIESTER PHOSPHODIESTERASE-LIKE PROTEIN-RELATED | *Vigna unguiculata* | 9 | 9 | 60.899 | 0.009 | 3.418 |
| Down | Vigun08g065300.1.p | rhamnose biosynthesis 1 | *Vigna unguiculata* | 2 | 2 | 9.678 | 0.023 | 2.896 |
| Down | Vigun02g135600.1.p | geranylgeranyl pyrophosphate synthase 1 | *Vigna unguiculata* | 9 | 8 | 75.601 | 0.009 | 2.588 |
| Down | Vigun02g178500.1.p | 2-C-methyl-D-erythritol 2,4-cyclodiphosphate synthase / MECDP-synthase | *Vigna unguiculata* | 4 | 4 | 34.044 | 0.021 | 5.029 |
| Down | Vigun03g161100.2.p | geranylgeranyl reductase | *Vigna unguiculata* | 6 | 6 | 63.093 | 0.019 | 2.113 |
| Down | Vigun05g174200.1.p | solanesyl diphosphate synthase 2 | *Vigna unguiculata* | 9 | 9 | 60.33 | 0.025 | 2.039 |
|  | **Membrane trafficking and intracellular transport** | |  |  |  |  |  |  |
| Up | Vigun08g157100.1.p | plasma membrane intrinsic protein 1;4 | *Vigna unguiculata* | 4 | 3 | 41.879 | 0.005 | 2.570 |
| Up | Vigun10g098200.1.p | lipid transfer protein 3 | *Vigna unguiculata* | 5 | 5 | 27.106 | 0.007 | 21.449 |
| Down | Vigun11g171500.1.p | Globin (Globin) | *Vigna unguiculata* | 6 | 6 | 19.813 | 0.034 | 9.395 |
| Down | Vigun03g033400.1.p | importin subunit beta-1 (KPNB1) | *Vigna unguiculata* | 3 | 3 | 10.349 | 0.017 | 2.040 |
| Down | Vigun09g206900.1.p | RAN GTPase 3 | *Vigna unguiculata* | 5 | 5 | 25.283 | 0.012 | 2.055 |
| Down | Vigun01g006000.1.p | NUCLEOID-ASSOCIATED PROTEIN YBAB | *Arabidopsis thaliana* | 10 | 9 | 78.374 | 0.019 | 2.128 |
| Down | Vigun03g110400.1.p | Clathrin light chain protein | *Vigna unguiculata* | 8 | 6 | 37.876 | 0.033 | 2.475 |
| Down | Vigun07g015200.1.p | syntaxin of plants 121 | *Vigna unguiculata* | 3 | 3 | 14.621 | 0.010 | 2.222 |
| Down | Vigun11g178100.1.p | Medium subunit of clathrin adaptor complex | *Vigna unguiculata* | 2 | 2 | 12.108 | 0.018 | 2.127 |
| Down | Vigun09g064800.2.p | DnaJ/Hsp40 cysteine-rich domain superfamily protein | *Arabidopsis thaliana* | 2 | 2 | 24.332 | 0.003 | 4.479 |
| Down | Vigun01g154600.1.p | endomembrane-type CA-ATPase 4 | *Arabidopsis thaliana* | 2 | 2 | 6.134 | 0.021 | 2.947 |
| Down | Vigun11g155500.1.p | mitochondrial import inner membrane translocase subunit TIM9 (TIM9) | *Arabidopsis thaliana* | 8 | 8 | 46.261 | 0.006 | 2.819 |
| Down | Vigun09g201300.1.p | H(+)-ATPase 5 | *Vigna unguiculata* | 8 | 3 | 30.260 | 0.016 | 3.114 |
| Down | Vigun07g204600.2.p | ATP synthase D chain, mitochondrial | *Vigna unguiculata* | 23 | 23 | 132.742 | 0.018 | 2.823 |
| Down | Vigun07g038900.1.p | copper chaperone | *Vigna unguiculata* | 3 | 3 | 16.778 | 0.011 | 2.878 |
| Down | Vigun07g194100.2.p | copper chaperone (ATOX1, ATX1, copZ) | *Vigna unguiculata* | 2 | 2 | 16.437 | 0.021 | 2.391 |
|  | **Photosynthesis** |  |  |  |  |  |  |  |
| Up | Vigun01g004300.1.p | photosystem I subunit G | *Vigna unguiculata* | 7 | 6 | 54.572 | 0.022 | 3.803 |
| Up | Vigun03g145400.1.p | light-harvesting chlorophyll-protein complex II subunit B1 | *Vigna unguiculata* | 13 | 3 | 129.512 | 0.017 | 2.023 |
| Up | Vigun04g103500.1.p | photosystem I light harvesting complex gene 1 | *Vigna unguiculata* | 7 | 7 | 46.577 | 0.045 | 2.392 |
| Up | Vigun05g056100.1.p | photosystem I subunit l | *Vigna unguiculata* | 5 | 5 | 70.956 | 0.01 | 2.215 |
| Up | Vigun06g224500.1.p | light harvesting complex photosystem II subunit 6 | *Vigna unguiculata* | 8 | 7 | 76.164 | 0.004 | 2.238 |
| Up | Vigun09g075200.1.p | light-harvesting chlorophyll-protein complex I subunit A4 | *Vigna unguiculata* | 8 | 8 | 84.17 | 0.017 | 2.26 |
| Up | VigunL041500.1.p | photosystem II reaction center protein A | *Vigna unguiculata* | 14 | 14 | 132.553 | 0.019 | 2.226 |
| Down | Vigun11g216900.1.p | Photosystem II 5 kD protein | *Arabidopsis thaliana* | 4 | 4 | 33.392 | 0.027 | 4.076 |
| Down | Vigun05g083700.1.p | nuclear shuttle interacting | *Arabidopsis thaliana* | 3 | 3 | 13.715 | 0.029 | 2.098 |
| Down | VigunL002500.1.p | ribulose-bisphosphate carboxylases | *Arabidopsis thaliana* | 16 | 4 | 84.886 | 0.035 | 5.969 |
| Down | Vigun07g083800.1.p | Tetratricopeptide repeat (TPR)-like superfamily protein | *Arabidopsis thaliana* | 4 | 4 | 19.341 | 0.020 | 2.886 |
| Down | Vigun11g165700.1.p | enzyme binding;tetrapyrrole binding | *Arabidopsis thaliana* | 3 | 3 | 12.653 | 0.036 | 2.035 |
| Down | Vigun01g115300.1.p | magnesium-chelatase subunit chlH, chloroplast, putative / Mg-protoporphyrin IX chelatase, putative (CHLH) | *Vigna unguiculata* | 9 | 9 | 58.299 | 0.023 | 3.487 |
|  | **Protein processing** |  |  |  |  |  |  |  |
| Up | Vigun10g040700.1.p | rotamase FKBP 1 | *Vigna unguiculata* | 9 | 8 | 40.946 | 0.009 | 3.536 |
| Down | Vigun07g159300.1.p | RUBISCO ACCUMULATION FACTOR 1, CHLOROPLASTIC-RELATED | *Arabidopsis thaliana* | 9 | 8 | 64.571 | 0.032 | 2.108 |
| Down | Vigun08g212400.1.p | HEAT SHOCK PROTEIN 81.4 | *Vigna unguiculata* | 47 | 28 | 349.227 | 0.017 | 2.307 |
| Down | Vigun03g011200.1.p | prefoldin 6 | *Vigna unguiculata* | 5 | 5 | 33.983 | 0.022 | 2.759 |
| Down | Vigun03g122700.1.p | mitochondrial HSO70 2 | *Vigna unguiculata* | 27 | 4 | 176.457 | 0.009 | 2.6737 |
| Down | Vigun05g054000.1.p | calreticulin 3 | *Vigna unguiculata* | 4 | 4 | 16.291 | 0.006 | 5.886 |
| Down | Vigun05g152000.2.p | like SEX4 1 | *Vigna unguiculata* | 2 | 2 | 5.737 | 0.013 | 5.075 |
|  | **Protein degradation** |  |  |  |  |  |  |  |
| Up | Vigun02g200500.1.p | ZINC METALLOPEPTIDASE EGY3, CHLOROPLASTIC-RELATED | *Arabidopsis thaliana* | 9 | 7 | 38.011 | 0.003 | 16.859 |
| Up | Vigun08g158300.3.p | ZINC METALLOPROTEASE EGY2, CHLOROPLASTIC-RELATED | *Vigna unguiculata* | 2 | 2 | 12.377 | 0.010 | 2.155 |
| Up | Vigun01g027800.1.p | ASPARTYL PROTEASES // ASPARTYL PROTEASE FAMILY PROTEIN-RELATED | *Vigna unguiculata* | 5 | 5 | 32.69 | 0.009 | 4.179 |
| Up | Vigun07g203500.1.p | serine carboxypeptidase-like 27 | *Vigna unguiculata* | 9 | 9 | 78.2182 | 0.005 | 4.046 |
| Up | Vigun03g031900.2.p | regulatory particle non-ATPase 13 | *Arabidopsis thaliana* | 2 | 2 | 4.057 | 0.028 | 2.474 |
| Down | Vigun03g343800.1.p | Beta-aspartyl-peptidase | *Arabidopsis thaliana* | 4 | 4 | 22.811 | 0.012 | 3.386 |
| Down | Vigun08g012200.1.p | regulatory particle triple-A ATPase 4A | *Vigna unguiculata* | 7 | 5 | 25.527 | 0.003 | 2.254 |
| Down | Vigun08g153700.1.p | Subtilisin-like serine endopeptidase family protein | *Vigna unguiculata* | 14 | 12 | 136.314 | 0.006 | 9.443 |
| Down | Vigun09g021700.1.p | FTSH protease 8 | *Vigna unguiculata* | 22 | 6 | 142.055 | 0.038 | 2.289 |
| Down | Vigun10g156100.1.p | regulatory particle triple-A 1A | *Vigna unguiculata* | 9 | 8 | 30.603 | 0.011 | 2.258 |
| Down | Vigun10g198500.1.p | regulatory particle triple-A ATPase 6A | *Vigna unguiculata* | 4 | 3 | 19.844 | 0.025 | 2.454 |
| Down | Vigun01g150300.1.p | regulatory particle AAA-ATPase 2A | *Vigna unguiculata* | 5 | 4 | 16.223 | 0.034 | 2.089 |
| Down | Vigun06g192100.1.p | Cysteine proteinase Cathepsin F // Cysteine proteinase Cathepsin L | *Vigna unguiculata* | 5 | 5 | 32.180 | 0.006 | 7.221 |
| Down | Vigun07g045200.2.p | SERINE PROTEASE FAMILY S10 SERINE CARBOXYPEPTIDASE | *Vigna unguiculata* | 4 | 4 | 26.201 | 0.019 | 2.035 |
| Down | Vigun07g111800.1.p | ASPARTYL PROTEASES // ASPARTYL PROTEASE-LIKE PROTEIN | *Vigna unguiculata* | 4 | 4 | 31.267 | 0.034 | 3.352 |
| Down | Vigun07g162200.2.p | Peptidase C12, ubiquitin carboxyl-terminal hydrolase 1 | *Vigna unguiculata* | 3 | 3 | 13.506 | 0.021 | 2.166 |
| Down | Vigun08g003500.1.p | RIBONUCLEASE P SUBUNIT P25 | *Vigna unguiculata* | 4 | 4 | 18.335 | 0.011 | 3.721 |
| Down | Vigun01g187500.1.p | Cysteine proteinases superfamily protein | *Arabidopsis thaliana* | 3 | 2 | 16.021 | 0.008 | 2.99997 |
|  | **Protein synthesis** |  |  |  |  |  |  |  |
| Up | Vigun05g021200.1.p | large subunit ribosomal protein L30e (RP-L30e, RPL30) | *Vigna unguiculata* | 2 | 2 | 4.21 | 0.023 | 2.139 |
| Up | Vigun05g182500.1.p | gamma-glutamyl hydrolase 1 | *Vigna unguiculata* | 17 | 15 | 163.781 | 0.010 | 7.391 |
| Up | Vigun05g182600.1.p | gamma-glutamyl hydrolase 2 | *Vigna unguiculata* | 4 | 4 | 34.707 | 0.021 | 2.021 |
| Down | Vigun03g316800.2.p | MA3 domain-containing protein | *Vigna unguiculata* | 3 | 3 | 18.698 | 0.009 | 2.436 |
| Down | Vigun09g140400.1.p | 30S ribosomal protein, putative | *Arabidopsis thaliana* | 8 | 7 | 94.203 | 0.048 | 2.016 |
| Down | Vigun06g200300.1.p | Eukaryotic translation initiation factor 3 subunit 7 (eIF-3) | *Vigna unguiculata* | 3 | 3 | 4.596 | 0.011 | 2.130 |
| Down | Vigun11g223300.1.p | translation initiation factor 3 subunit I (EIF3I) | *Arabidopsis thaliana* | 8 | 7 | 29.683 | 0.017 | 2.221 |
| Down | Vigun01g156600.1.p | large subunit ribosomal protein L4e (RP-L4e, RPL4) | *Vigna unguiculata* | 11 | 7 | 63.571 | 0.007 | 2.268 |
| Down | Vigun01g188000.1.p | Peptide chain release factor 1 | *Vigna unguiculata* | 4 | 3 | 14.593 | 0.012 | 2.299 |
| Down | Vigun02g164500.1.p | ATP binding;leucine-tRNA ligases;aminoacyl-tRNA ligases;nucleotide binding;ATP binding;aminoacyl-tRNA ligases | *Vigna unguiculata* | 7 | 7 | 21.722 | 0.004 | 3.407 |
| Down | Vigun04g109100.3.p | eukaryotic release factor 1-3 | *Vigna unguiculata* | 4 | 4 | 9.715 | 0.010 | 2.247 |
| Down | Vigun05g301500.1.p | threonyl-tRNA synthetase, putative / threonine--tRNA ligase, putative | *Vigna unguiculata* | 3 | 2 | 4.279 | 0.042 | 2.164 |
| Down | Vigun06g168800.1.p | TRANSLATION FACTOR | *Vigna unguiculata* | 13 | 12 | 74.812 | 0.002 | 2.31 |
| Down | Vigun07g011000.1.p | Eukaryotic initiation factor 4E protein | *Vigna unguiculata* | 4 | 4 | 14.957 | 0.013 | 2.101 |
| Down | Vigun07g133800.1.p | Glutamyl/glutaminyl-tRNA synthetase, class Ic | *Vigna unguiculata* | 3 | 2 | 18.875 | 0.007 | 2.08 |
| Down | Vigun07g144000.1.p | large subunit ribosomal protein L22e (RP-L22e, RPL22) | *Vigna unguiculata* | 4 | 2 | 22.452 | 0.020 | 2.445 |
| Down | Vigun08g135500.1.p | eukaryotic translation initiation factor 2 beta subunit | *Vigna unguiculata* | 2 | 2 | 3.999 | 0.009 | 2.867 |
| Down | Vigun09g026100.5.p | lysyl-tRNA synthetase 1 | *Vigna unguiculata* | 5 | 5 | 14.820 | 0.020 | 2.437 |
|  | **Stress-related proteins: defense response** | |  |  |  |  |  |  |
| Up | Vigun05g143200.1.p | drought-repressed 4 | *Arabidopsis thaliana* | 6 | 5 | 50.779 | 0.015 | 8.696 |
| Up | Vigun05g143600.1.p | drought-repressed 4 | *Arabidopsis thaliana* | 3 | 3 | 8.328 | 0.010 | 5.149 |
| Up | Vigun05g143700.1.p | kunitz trypsin inhibitor 1 | *Arabidopsis thaliana* | 4 | 3 | 30.806 | 0.024 | 2.4159 |
| Up | Vigun05g143800.1.p | kunitz trypsin inhibitor 1 | *Arabidopsis thaliana* | 5 | 5 | 20.659 | 0.004 | 4.026 |
| Up | Vigun05g144000.1.p | drought-repressed 4 | *Arabidopsis thaliana* | 5 | 2 | 38.166 | 0.004 | 26.053 |
| Up | Vigun05g145700.1.p | kunitz trypsin inhibitor 1 | *Arabidopsis thaliana* | 8 | 5 | 112.762 | 0.009 | 30.411 |
| Up | Vigun05g146000.1.p | kunitz trypsin inhibitor 1 | *Arabidopsis thaliana* | 9 | 7 | 108.790 | 0.023 | 23.447 |
| Up | Vigun05g146100.1.p | kunitz trypsin inhibitor 1 | *Arabidopsis thaliana* | 11 | 7 | 159.4 | 0.019 | 32.951 |
| Up | Vigun05g143400.1.p | Kunitz family trypsin and protease inhibitor protein | *Arabidopsis thaliana* | 5 | 5 | 63.993 | 0.03 | 5.021 |
| Up | Vigun05g145900.1.p | Kunitz family trypsin and protease inhibitor protein | *Arabidopsis thaliana* | 14 | 12 | 150.274 | 0.006 | 31.726 |
| Up | Vigun10g170100.1.p | lipoxygenase 1 | *Arabidopsis thaliana* | 17 | 16 | 65.854 | 0.009 | 6.181 |
| Up | Vigun11g030900.1.p | Pathogenesis-related protein Bet v I family (Bet_v_1) | *Vigna unguiculata* | 4 | 3 | 23.909 | 0.008 | 6.745 |
| Up | Vigun09g243000.1.p | allene oxide synthase | *Arabidopsis thaliana* | 12 | 11 | 50.474 | 0.046 | 2.611 |
| Down | Vigun06g213100.1.p | basic pathogenesis-related protein 1 | *Vigna unguiculata* | 8 | 7 | 74.646 | 0.024 | 12.093 |
| Down | Vigun01g215400.1.p | ALWAYS EARLY 4 | *Arabidopsis thaliana* | 3 | 3 | 19.933 | 0.039 | 3.072 |
| Down | Vigun10g125300.1.p | (+)-neomenthol dehydrogenase (E1.1.1.208) | *Arabidopsis thaliana* | 6 | 4 | 24.449 | 0.025 | 2.204 |
| Down | Vigun06g113300.1.p | CHITINASE-RELATED // HEVEIN-LIKE PREPROPROTEIN | *Vigna unguiculata* | 3 | 3 | 22.207 | 0.009 | 3.918 |
| Down | Vigun03g131200.1.p | Pathogenesis-related protein Bet v I family (Bet_v_1) | *Vigna unguiculata* | 6 | 2 | 28.287 | 0.014 | 4.91 |
| Down | Vigun03g418100.1.p | Pathogenesis-related protein Bet v I family (Bet_v_1) | *Vigna unguiculata* | 10 | 2 | 55.752 | 0.003 | 5.49 |
| Down | Vigun03g418400.1.p | Pathogenesis-related protein Bet v I family (Bet_v_1) | *Vigna unguiculata* | 15 | 14 | 90.698 | 0.007 | 6.22 |
| Down | Vigun03g252000.1.p | basic chitinase | *Vigna unguiculata* | 2 | 2 | 12.263 | 0.003 | 5.955 |
| Down | Vigun09g278000.1.p | basic chitinase | *Vigna unguiculata* | 2 | 2 | 18.905 | 0.024 | 3.558 |
|  | **Stress-related proteins: heat stress response** | |  |  |  |  |  |  |
| Up | Vigun11g007700.1.p | annexin 8 | *Arabidopsis thaliana* | 17 | 17 | 117.845 | 0.004 | 3.099 |
| Up | Vigun04g168600.1.p | 22.0 KDA HEAT SHOCK PROTEIN | *Arabidopsis thaliana* | 5 | 5 | 14.563 | 0.036 | 5.879 |
| Up | Vigun03g104500.1.p | 17.6 KDA CLASS I HEAT SHOCK PROTEIN 1-RELATED | *Arabidopsis thaliana* | 5 | 3 | 22.289 | 0.038 | 10.17 |
| Up | Vigun07g167700.1.p | 22.0 KDA HEAT SHOCK PROTEIN | *Arabidopsis thaliana* | 10 | 9 | 67.201 | 0.029 | 15.36 |
| Down | Vigun04g144500.1.p | phosphatase-related | *Arabidopsis thaliana* | 6 | 5 | 21.477 | 0.013 | 2.523 |
| Down | Vigun07g183900.1.p | plastid transcriptionally active 5 | *Arabidopsis thaliana* | 5 | 5 | 7.686 | 0.015 | 2.313 |
| Down | Vigun01g237400.2.p | MOLECULAR CHAPERONE DNAJ | *Vigna unguiculata* | 7 | 3 | 46.016 | 0.006 | 2.339 |
| Down | Vigun05g230700.1.p | DNAJ homologue 2 | *Vigna unguiculata* | 5 | 5 | 30.127 | 0.013 | 2.096 |
| Down | Vigun07g086100.1.p | heat shock cognate protein 70-1 | *Arabidopsis thaliana* | 38 | 4 | 276.358 | 0.045 | 2.067 |
|  | **Stress-related proteins: oxidative stress response and redox homeostasis** | |  |  |  |  |  |  |
| Up | Vigun10g178300.1.p | HAD superfamily, subfamily IIIB acid phosphatase | *Arabidopsis thaliana* | 14 | 14 | 100.982 | 0.012 | 10.054 |
| Up | Vigun10g096100.1.p | Peroxidase / Lactoperoxidase | *Vigna unguiculata* | 6 | 5 | 24.631 | 0.007 | 2.999 |
| Up | Vigun03g306600.1.p | copper chaperone for SOD1 | *Vigna unguiculata* | 4 | 4 | 14.239 | 0.033 | 2.272 |
| Down | Vigun09g097000.1.p | peptidemethionine sulfoxide reductase 1 | *Vigna unguiculata* | 6 | 5 | 35.211 | 0.009 | 2.722 |
| Down | Vigun11g012800.1.p | NADH dehydrogenase (ubiquinone) 1 alpha subcomplex subunit 6 (NDUFA6) | *Arabidopsis thaliana* | 5 | 5 | 18.44 | 0.044 | 2.079 |
| Down | Vigun07g213900.1.p | Peroxidase / Lactoperoxidase | *Vigna unguiculata* | 14 | 14 | 83.368 | 0.018 | 2.393 |
| Down | Vigun02g189400.1.p | NADH dehydrogenase (ubiquinone) 1 alpha subcomplex subunit 12 (NDUFA12) | *Arabidopsis thaliana* | 5 | 5 | 25.222 | 0.048 | 2.038 |
| Down | Vigun01g027500.1.p | Thioredoxin z | *Vigna unguiculata* | 5 | 3 | 24.780 | 0.025 | 2.198 |
| Down | Vigun03g236100.1.p | nucleoredoxin [EC:1.8.1.8] (NXN) | *Arabidopsis thaliana* | 10 | 9 | 49.605 | 0.035 | 2.591 |
| Down | Vigun09g238200.3.p | THIOREDOXIN // THIOREDOXIN-LIKE 4, CHLOROPLASTIC | *Vigna unguiculata* | 7 | 7 | 37.876 | 0.025 | 2.019 |
| Down | Vigun10g188100.1.p | glutaredoxin 4 | *Vigna unguiculata* | 3 | 3 | 16.229 | 0.027 | 2.501 |
|  | **Stress-related proteins: nutrient stress response and homeostasis** | |  |  |  |  |  |  |
| Up | Vigun06g118300.1.p | Li-tolerant lipase 1 | *Arabidopsis thaliana* | 2 | 2 | 12.322 | 0.015 | 3.113 |
| Up | Vigun11g209200.2.p | mitochondrion-localized small heat shock protein 23.6 | *Arabidopsis thaliana* | 9 | 9 | 43.640 | 0.016 | 10.499 |
| Up | Vigun03g000700.1.p | D-cysteine desulfhydrase | *Arabidopsis thaliana* | 7 | 7 | 31.565 | 0.027 | 2.241 |
| Down | Vigun07g133300.1.p | DEAD box RNA helicase (RH3) | *Arabidopsis thaliana* | 14 | 13 | 87.049 | 0.006 | 2.031 |
| Down | Vigun04g165700.1.p | Iron-binding zinc finger CDGSH type (zf-CDGSH) | *Arabidopsis thaliana* | 5 | 4 | 62.306 | 0.023 | 3.993 |
|  | **Stress-related proteins: water deprivation stress response** | |  |  |  |  |  |  |
| Down | Vigun10g050500.1.p | ALDO-KETO REDUCTASE FAMILY 4 MEMBER C10 | *Arabidopsis thaliana* | 17 | 3 | 123.374 | 0.023 | 2.502 |
|  | **Stress-related proteins: other stress responses** | |  |  |  |  |  |  |
| Up | Vigun04g130500.1.p | ABA/WDS induced protein (ABA_WDS) | *Vigna unguiculata* | 11 | 11 | 141.332 | 0.044 | 4.359 |
| Up | VigunL078600.1.p | heat shock protein 90.1 | *Vigna unguiculata* | 32 | 18 | 202.506 | 0.009 | 4.331 |
| Up | Vigun03g397300.1.p | germin 3 | *Arabidopsis thaliana* | 3 | 3 | 49.578 | 0.020 | 3.201 |
| Down | Vigun06g003600.1.p | hypoxia up-regulated 1 (HYOU1) | *Arabidopsis thaliana* | 2 | 2 | 3.677 | 0.006 | 4.246 |
|  | **Transcription** |  |  |  |  |  |  |  |
| Up | Vigun04g040700.1.p | Stabilizer of iron transporter SufD / Polynucleotidyl transferase | *Vigna unguiculata* | 4 | 4 | 13.863 | 0.009 | 4.387 |
| Down | Vigun09g273700.1.p | cold shock domain protein 1 | *Vigna unguiculata* | 4 | 3 | 42.029 | 0.033 | 2.406 |
| Down | Vigun01g139900.1.p | RNA-binding KH domain-containing protein | *Arabidopsis thaliana* | 3 | 3 | 15.651 | 0.012 | 2.286 |
| Down | Vigun09g169400.1.p | cold shock domain protein 1 | *Vigna unguiculata* | 2 | 2 | 11.81 | 0.033 | 2.549 |
| Down | Vigun05g256200.3.p | THO complex subunit 4 (THOC4, ALY) | *Vigna unguiculata* | 7 | 7 | 36.221 | 0.020 | 2.441 |
|  | **Other metabolisms** |  |  |  |  |  |  |  |
| Up | Vigun10g051600.2.p | rubisco activase | *Arabidopsis thaliana* | 33 | 7 | 437.04 | 0.010 | 4.622 |
| Up | Vigun01g050400.1.p | glutathione S-transferase TAU 25 | *Medicago truncatula* | 5 | 3 | 11.122 | 0.044 | 3.054 |
| Up | Vigun03g111900.1.p | Catechol oxidase / Tyrosinase // Tyrosinase / Tyrosine-dopa oxidase | *Vigna unguiculata* | 9 | 5 | 48.020 | 0.034 | 2.444 |
| Up | Vigun01g025400.1.p | Copper amine oxidase family protein | *Vigna unguiculata* | 8 | 2 | 24.827 | 0.008 | 2.863 |
| Up | Vigun11g212700.1.p | cytokinin oxidase/dehydrogenase 6 | *Vigna unguiculata* | 15 | 14 | 185.156 | 0.034 | 2.701 |
| Up | Vigun06g103200.1.p | GLUCOSE AND RIBITOL DEHYDROGENASE HOMOLOG 1-RELATED | *Vigna unguiculata* | 3 | 3 | 22.745 | 0.006 | 3.114 |
| Up | Vigun03g313300.2.p | purple acid phosphatase 27 | *Arabidopsis thaliana* | 4 | 4 | 38.223 | 0.016 | 2.0742 |
| Up | Vigun05g280600.1.p | GAMMA-BUTYROBETAINE HYDROXYLASE-RELATED | *Vigna unguiculata* | 3 | 3 | 14.439 | 0.001 | 7.776 |
| Up | Vigun08g068600.1.p | alcohol dehydrogenase 1 | *Vigna unguiculata* | 3 | 2 | 10.616 | 0.017 | 12.244 |
| Up | Vigun11g202900.1.p | NAD DEPENDENT EPIMERASE/DEHYDRATASE // ALCOHOL DEHYDROGENASE-RELATED | *Arabidopsis thaliana* | 28 | 5 | 269.061 | 0.009 | 2.361 |
| Up | Vigun03g445700.2.p | LACTOYLGLUTATHIONE LYASE GLYOXALASE I // GLYOXALASE I HOMOLOG | *Arabidopsis thaliana* | 2 | 2 | 9.331 | 0.006 | 2.181 |
| Up | Vigun07g006600.1.p | cyanase | *Vigna unguiculata* | 8 | 6 | 50.096 | 0.044 | 2.843 |
| Up | Vigun03g228000.1.p | elicitor-activated gene 3-2 | *Arabidopsis thaliana* | 5 | 5 | 21.600 | 0.009 | 3.781 |
| Up | Vigun04g016500.2.p | aldehyde dehydrogenase 2C4 | *Arabidopsis thaliana* | 9 | 6 | 33.981 | 0.013 | 3.715 |
| Up | Vigun11g202800.1.p | Cinnamyl-alcohol dehydrogenase / CAD | *Arabidopsis thaliana* | 9 | 2 | 37.952 | 0.010 | 15.094 |
| Up | Vigun03g340700.2.p | heat shock protein 101 | *Vigna unguiculata* | 13 | 10 | 62.31 | 0.004 | 3.864 |
| Down | Vigun06g221300.1.p | GTP cyclohydrolase II | *Vigna unguiculata* | 2 | 2 | 8.493 | 0.016 | 2.578 |
| Down | Vigun03g303000.1.p | peroxin 19-1 | *Vigna unguiculata* | 2 | 2 | 10.518 | 0.021 | 2.331 |
| Down | Vigun04g187300.1.p | formate dehydrogenase | *Vigna unguiculata* | 6 | 6 | 16.736 | 0.009 | 3.373 |
| Down | Vigun01g108000.1.p | Trans-aconitate 3-methyltransferase | *Vigna unguiculata* | 6 | 6 | 61.957 | 0.009 | 3.135 |
| Down | Vigun01g199100.1.p | NFU domain protein 1 | *Vigna unguiculata* | 2 | 2 | 20.401 | 0.029 | 3.042 |
| Down | Vigun03g150700.1.p | 3'(2'), 5'-bisphosphate nucleotidase / inositol polyphosphate 1-phosphatase (SAL) | *Vigna unguiculata* | 2 | 2 | 9.992 | 0.045 | 2.010 |
| Down | Vigun11g191600.1.p | Cytokine-induced anti-apoptosis inhibitor 1, Fe-S biogenesis | *Vigna unguiculata* | 2 | 2 | 12.998 | 0.017 | 5.058 |
| Down | Vigun10g052700.3.p | uracil phosphoribosyltransferase | *Vigna unguiculata* | 10 | 10 | 80.473 | 0.039 | 2.011 |
| Down | Vigun11g176100.1.p | NAD DEPENDENT EPIMERASE/DEHYDRATASE | *Arabidopsis thaliana* | 17 | 15 | 184.949 | 0.006 | 2.133 |
| Down | Vigun02g129000.1.p | 2'-hydroxyisoflavone reductase | *Arabidopsis thaliana* | 5 | 2 | 22.862 | 0.005 | 6.852 |
| Down | Vigun04g016900.1.p | aldehyde dehydrogenase 2C4 | *Arabidopsis thaliana* | 15 | 12 | 72.571 | 0.016 | 2.170 |
| Down | Vigun08g172400.1.p | OXIDOREDUCTASE, 2OG-FE II OXYGENASE FAMILY PROTEIN // 1-AMINOCYCLOPROPANE-1-CARBOXYLATE OXIDASE 3-RELATED | *Arabidopsis thaliana* | 5 | 5 | 21.095 | 0.029 | 2.039 |
| Down | Vigun02g194800.1.p | NADH-CYTOCHROME B5 REDUCTASE | *Vigna unguiculata* | 3 | 3 | 25.503 | 0.009 | 5.652 |
| Down | Vigun11g123700.1.p | Secoisolariciresinol dehydrogenase | *Vigna unguiculata* | 4 | 3 | 21.624 | 0.029 | 2.935 |
| Down | Vigun05g115000.1.p | KR domain (KR) // Enoyl-(Acyl carrier protein) reductase (adh_short_C2) | *Vigna unguiculata* | 4 | 2 | 22.223 | 0.007 | 3.445 |
| Down | Vigun02g094300.1.p | SHIKIMATE KINASE // INACTIVE SHIKIMATE KINASE LIKE 2, CHLOROPLASTIC-RELATED | *Arabidopsis thaliana* | 5 | 5 | 32.915 | 0.005 | 2.116 |
| Down | Vigun03g233700.1.p | Alpha-glucan, water dikinase / Starch-related R1 protein | *Vigna unguiculata* | 26 | 25 | 157.626 | 0.047 | 2.316 |
| Down | Vigun03g041300.1.p | monodehydroascorbate reductase 6 | *Vigna unguiculata* | 8 | 8 | 39.579 | 0.042 | 2.009 |
| Down | Vigun03g287600.1.p | NADH-ubiquinone oxidoreductase-related | *Vigna unguiculata* | 3 | 2 | 4.15 | 0.009 | 2.292 |
| Down | Vigun03g413500.1.p | DNA-binding enhancer protein-related | *Vigna unguiculata* | 3 | 3 | 2.496 | 0.034 | 3.415 |
| Down | Vigun05g259800.1.p | Haloacid dehalogenase-like hydrolase (HAD) superfamily protein | *Vigna unguiculata* | 2 | 2 | 5.396 | 0.017 | 6.663 |
| Down | Vigun09g245700.1.p | Leucine carboxyl methyltransferase | *Vigna unguiculata* | 2 | 2 | 9.925 | 0.021 | 2.387 |
|  | **Function unknown** |  |  |  |  |  |  |  |
| Up | Vigun01g038000.1.p | Cinnamyl-alcohol dehydrogenase / CAD | *Vigna unguiculata* | 10 | 2 | 44.191 | 0.012 | 2.779 |
| Up | Vigun03g085900.2.p | Cupin (Cupin_1) | *Vigna unguiculata* | 5 | 5 | 22.175 | 0.028 | 6.575 |
| Up | Vigun01g243900.1.p | heat shock protein 21 | *Vigna unguiculata* | 11 | 11 | 59.075 | 0.006 | 6.006 |
| Up | Vigun06g052200.1.p | heat shock protein 21 | *Vigna unguiculata* | 21 | 21 | 146.279 | 0.008 | 34.401 |
| Up | Vigun09g194100.1.p | heat stable protein 1 | *Vigna unguiculata* | 6 | 6 | 47.107 | 0.014 | 4.17 |
| Up | Vigun07g048400.1.p | Concanavalin A-like lectin protein kinase family protein | *Vigna unguiculata* | 16 | 14 | 152.766 | 0.003 | 8.18 |
| Up | Vigun07g226500.1.p | Concanavalin A-like lectin protein kinase family protein | *Vigna unguiculata* | 5 | 4 | 51.18 | 0.025 | 2.747 |
| Up | Vigun07g226600.1.p | Concanavalin A-like lectin protein kinase family protein | *Vigna unguiculata* | 3 | 2 | 29.092 | 0.027 | 6.116 |
| Up | Vigun08g182100.1.p | cystatin B | *Vigna unguiculata* | 13 | 10 | 104.997 | 0.006 | 6.809 |
| Up | Vigun10g064900.1.p | cystatin B | *Vigna unguiculata* | 6 | 2 | 35.863 | 0.016 | 2.199 |
| Up | Vigun11g041400.1.p | SOUL heme-binding family protein | *Vigna unguiculata* | 12 | 12 | 103.223 | 0.006 | 2.267 |
| Up | Vigun03g255200.2.p | Uncharacterized protein | *Vigna unguiculata* | 3 | 3 | 10.042 | 0.009 | 2.002 |
| Up | Vigun06g009100.1.p | Uncharacterized protein | *Vigna unguiculata* | 2 | 2 | 2.4833 | 0.008 | 7.427 |
| Up | Vigun04g134800.1.p | Alliin lyase / L-cysteine sulfoxide lyase | *Vigna unguiculata* | 22 | 22 | 153.989 | 0.013 | 4.067 |
| Up | Vigun10g053000.1.p | CLPC homologue 1 | *Vigna unguiculata* | 38 | 13 | 215.16 | 0.005 | 4.6775 |
| Down | Vigun03g250900.1.p | Disease resistance-responsive (dirigent-like protein) family protein | *Vigna unguiculata* | 3 | 3 | 21.672 | 0.046 | 2.246 |
| Down | Vigun01g186100.1.p | Uncharacterized protein | *Vigna unguiculata* | 3 | 3 | 23.537 | 0.015 | 2.234 |
| Down | Vigun01g234400.1.p | apoptotic chromatin condensation inducer in the nucleus (ACIN1, ACINUS) | *Vigna unguiculata* | 7 | 5 | 22.441 | 0.05 | 2.649 |
| Down | Vigun02g202800.1.p | SWIB/MDM2 domain superfamily protein | *Vigna unguiculata* | 4 | 4 | 14.38 | 0.034 | 2.138 |
| Down | Vigun03g059500.1.p | CBS / octicosapeptide/Phox/Bemp1 (PB1) domains-containing protein | *Vigna unguiculata* | 6 | 6 | 35.585 | 0.016 | 2.009 |
| Down | Vigun05g148500.1.p | Predicted phosphoglycerate mutase | *Vigna unguiculata* | 3 | 2 | 5.268 | 0.004 | 2.274 |
| Down | Vigun05g183600.1.p | pathogenesis-related family protein | *Vigna unguiculata* | 3 | 3 | 4.205 | 0.016 | 2.308 |
| Down | Vigun06g089200.1.p | CT120 PROTEIN // SUBFAMILY NOT NAMED | *Vigna unguiculata* | 7 | 5 | 29.523 | 0.016 | 2.678 |
| Down | Vigun07g189600.2.p | Galactose oxidase/kelch repeat superfamily protein | *Vigna unguiculata* | 3 | 3 | 14.736 | 0.008 | 7.598 |
| Down | Vigun11g177200.1.p | small subunit ribosomal protein S25e (RP-S25e, RPS25) | *Vigna unguiculata* | 8 | 8 | 62.169 | 0.021 | 2.101 |
| Down | Vigun07g192700.1.p | Uncharacterized protein | *Vigna unguiculata* | 2 | 2 | 5.5433 | 0.019 | 3.939 |
| Down | Vigun10g173900.1.p | Uncharacterized protein | *Vigna unguiculata* | 3 | 3 | 13.925 | 0.035 | 2.681 |
| Down | Vigun11g033800.1.p | Calcium-binding EF-hand family protein | *Vigna unguiculata* | 2 | 2 | 26.894 | 0.013 | 2.202 |
| Down | Vigun11g040500.1.p | GTP-binding protein-related | *Vigna unguiculata* | 7 | 7 | 31.116 | 0.004 | 2.693 |
| Down | Vigun03g185300.1.p | alpha/beta-Hydrolases superfamily protein | *Vigna unguiculata* | 5 | 5 | 33.764 | 0.007 | 2.143 |
| Down | Vigun06g122300.1.p | GLUCOSYL/GLUCURONOSYL TRANSFERASES // UDP-GLYCOSYLTRANSFERASE 72B2-RELATED | *Vigna unguiculata* | 2 | 2 | 6.572 | 0.009 | 2.238 |
| Down | Vigun06g012000.2.p | NUCLEAR INHIBITOR OF PROTEIN PHOSPHATASE-1 | *Vigna unguiculata* | 11 | 10 | 144.199 | 0.013 | 2.013 |
| Down | Vigun07g227900.1.p | membrane-associated progesterone binding protein 3 | *Vigna unguiculata* | 4 | 3 | 21.752 | 0.006 | 3.672 |
| Down | Vigun08g003400.1.p | RIBONUCLEASE P SUBUNIT P25 | *Vigna unguiculata* | 3 | 3 | 18.445 | 0.006 | 4.659 |
| Down | Vigun02g066100.1.p | glycine-rich protein | *Vigna unguiculata* | 4 | 4 | 31.398 | 0.01 | 3.178 |
| Down | Vigun02g018300.1.p | Sec14p-like phosphatidylinositol transfer family protein | *Vigna unguiculata* | 2 | 2 | 10.024 | 0.023 | 2.242 |
